# Phylogenetic classification of bony fishes

**DOI:** 10.1186/s12862-017-0958-3

**Published:** 2017-07-06

**Authors:** Ricardo Betancur-R, Edward O. Wiley, Gloria Arratia, Arturo Acero, Nicolas Bailly, Masaki Miya, Guillaume Lecointre, Guillermo Ortí

**Affiliations:** 1 Department of Biology, University of Puerto Rico, Río Piedras, P.O. Box 23360, San Juan, PR 00931 USA; 2 Department of Vertebrate Zoology, National Museum of Natural History, Smithsonian Institution, Washington, DC USA; 3 Biodiversity Institute and Department of Ecology & Evolutionary Biology, University of Kansas, Lawrence, KS USA; 4 Sam Houston State Natural History Collections, Sam Houston State University, Huntsville, Texas USA; 5 Universidad Nacional de Colombia sede Caribe, Cecimar, El Rodadero, Santa Marta, Magdalena Colombia; 6 FishBase Information and Research Group, Los Baños, Philippines; 7 Department Ecology and Environmental Sciences, Natural History Museum and Institute, Chiba, Japan; 8 Institut de Systématique, Evolution, Biodiversité (ISYEB), Muséum National d’Histoire Naturelle, Paris, France; 9 Department of Biology, The George Washington University, Washington, DC USA

## Abstract

**Background:**

Fish classifications, as those of most other taxonomic groups, are being transformed drastically as new molecular phylogenies provide support for natural groups that were unanticipated by previous studies. A brief review of the main criteria used by ichthyologists to define their classifications during the last 50 years, however, reveals slow progress towards using an explicit phylogenetic framework. Instead, the trend has been to rely, in varying degrees, on deep-rooted anatomical concepts and authority, often mixing taxa with explicit phylogenetic support with arbitrary groupings. Two leading sources in ichthyology frequently used for fish classifications (JS Nelson’s volumes of *Fishes of the World* and W. Eschmeyer’s *Catalog of Fishes*) fail to adopt a global phylogenetic framework despite much recent progress made towards the resolution of the fish Tree of Life. The first explicit phylogenetic classification of bony fishes was published in 2013, based on a comprehensive molecular phylogeny (www.deepfin.org). We here update the first version of that classification by incorporating the most recent phylogenetic results.

**Results:**

The updated classification presented here is based on phylogenies inferred using molecular and genomic data for nearly 2000 fishes. A total of 72 orders (and 79 suborders) are recognized in this version, compared with 66 orders in version 1. The phylogeny resolves placement of 410 families, or ~80% of the total of 514 families of bony fishes currently recognized. The ordinal status of 30 percomorph families included in this study, however, remains uncertain (*incertae sedis* in the series Carangaria, Ovalentaria, or Eupercaria). Comments to support taxonomic decisions and comparisons with conflicting taxonomic groups proposed by others are presented. We also highlight cases were morphological support exist for the groups being classified.

**Conclusions:**

This version of the phylogenetic classification of bony fishes is substantially improved, providing resolution for more taxa than previous versions, based on more densely sampled phylogenetic trees. The classification presented in this study represents, unlike any other, the most up-to-date hypothesis of the Tree of Life of fishes.

**Electronic supplementary material:**

The online version of this article (doi:10.1186/s12862-017-0958-3) contains supplementary material, which is available to authorized users.

“*Characterem non constituero Genus, sed Genus Characterem*” – C Linnaeus [1].

“*Such expressions as that famous one of Linnaeus* [1] *... that the characters do not make the genus, but that the genus gives the characters, seem to imply that something more is included in our classifications, than mere resemblance. I believe that something more is included; and that propinquity of descent – the only known cause of the similarity of organic beings – is the bond, hidden as it is by various degrees of modification, which is partially revealed to us by our classifications.*”

− CR Darwin [2].

“*These guys knew what they were talking about! It is kind of amazing that Linnaeus* [1] *made the first statement, even though he did not yet fully understand evolutionary relationships (propinquity of descent in Darwin’s words* [2]*) as the underlying basis of those higher taxa. It is a shame that this basic and important principle of life is still not understood by the majority of people... even many practicing biologists! Characters do not “define” taxa; taxa are “defined” by their common ancestry (just like other historical groups, like human families). Because taxa share a common ancestry, they often share many characters, which we may use to recognize them. But if one of the species in a taxon lacks one of those characters (but is still clearly part of the group), it is still part of the taxon. It is one of the simplest and most fundamental ideas in biology, and yet so many people (even biologists) seem not to understand this simple concept*.”

– D Hillis [3].

*“Since taxonomy tends, ideally, not toward just any type of convenient classification of living forms… but toward a phyletic classification, and since the comparison of the structure of homologous informational macro-molecules allows the establishment of phylogenetic relationships, studies of chemical paleogenetics have a bearing on taxonomy.”*

– E Zuckerkandl and L Pauling [4].

“*The conflict between these two approaches, the former which could be called phenetic (or typological), and the latter which could be called phyletic (or evolutionary), is not a new conflict, but to the uninitiated it gives the ichthyological literature something of a chaotic aspect. The situation is not improved by authors who are neither strictly phenetic nor phyletic in approach… In the writer’s opinion, we ultimately will have a purely phyletic classification, and this will be achieved in relation to our progress in unraveling the phyletic interrelationships of the Recent fishes*… *There is little doubt that the methods of comparative biology are adequate for revealing ancestral conditions, even without knowledge of ancestor-descendant relationships among organisms of the past. Such knowledge will never be available to us, for only in the genetics laboratory, and for organisms of the present, is such knowledge possible.*”

– G Nelson [5].

## Background

Classification is an integral part of all sciences. The basis for classifications differs between disciplines but the basic principles are the same— in all cases we seek to understand something fundamental about the things classified. For astronomers, it is understanding the mass-luminosity relationships that lead to unraveling stellar evolution. For chemists, it is understanding how the atomic structure of elements leads to knowing how reactions occur. For systematists, it is understanding the relationships of organisms in the Tree of Life. The meaning of “relationship” in systematics has changed over time, but today it unquestionably means the genealogical affinities produced by the history of evolutionary descent. Notions of grades or levels of organization (shades of Lamarck or the *Scala Naturae*) are displaced by understanding that if a classification is organized strictly according to our best estimate of the Tree of Life, the organization of organisms becomes more predictive and straightforward, just as knowing the mass-luminosity relationships of a star will predict its future evolution or knowing that since the orbitals of a helium atom are full it is likely to not react with an atom of oxygen. Beyond doubt, the principles of phylogenetic systematics are now accepted as a rule; the most useful classification of organisms is that advocated, though never achieved, by Darwin.

The “modern era” classification of fishes is considered by many to begin in 1966 with the publication of a provisional classification of teleosts based on “phyletic thinking” [6]. Prior to this work, the most general classification in use had been proposed by LS Berg [7], from which the endings of modern orders (“-formes”) were retained. PH Greenwood, DE Rosen, SH Weitzman and GS Myers [6] turned the attention of systematic ichthyologists of the day toward classifications that reflected the perceived evolutionary histories of fishes. Many modern clades were not only recognized, they were coupled with explicit characterizations. Many of these characterizations turned out to be synapomorphies supporting many of the clades still recognized today. The work stands as the last pre-cladistic general classification of fishes, revolutionary in that there was explicit phyletic thinking, and yet arranged more along the lines of Simpson’s classification of mammals with its reliance on grades of organization and ancestral groups than on the concepts of strict monophyly and sister-group relationships we recognize today. But, importantly to subsequent developments, PH Greenwood, DE Rosen, SH Weitzman and GS Myers [6] rejected two things, phenetics (group taxa based solely on apparent similarity) and the central role of fossils to classification of recent fishes. Today, fossils are important, of course, not only because they allow estimating divergence times via molecular clock calibrations [8–14], but also because it is becoming increasingly clear that integrating paleontological and neontological data improves our understanding of the Tree of Life of fishes [15–25] and their macroevolutionary history [26–30].

The first explicitly phylogenetic classification of fishes was published by G Nelson [5] together with a clear discussion of the principles of phylogenetic systematics. Although at the time “phyletic interrelationships” among the included species and higher taxa were quite controversial, G Nelson [5] presented simple cladograms based on earlier views of vertebrate evolution (e.g., [31]) to justify his classification. His proposal discarded the use of grades and ancestral groups and rejected the idea that “gaps,” rates of change, or any other criterion previously accepted by evolutionary systematists [32], could be used to justify elevating the rank of a particular group higher than that of its closest relative. Thus, birds are classified with crocodiles in Archosauria and the entire clade of tetrapods is found within Sarcopterygii. The revolution had begun, spurred on by publication of the multi-authored *Interrelationship of Fishes* [33]. It is not our place to detail this revolution, it happened slowly as investigators learned how to infer phylogenies and translate their findings into explicit phylogenetic classifications [34]. Many of these changes to fish classifications in general and phylogenetic classification in particular are summarized in DE Rosen [35], GV Lauder and KF Liem [36], and M Stiassny, L Parenti and G Johnson [37]. They are reflected to a greater or lesser degree in various editions of JS Nelson’s *Fishes of the World* [38–42]. Of particular interest is the observation that much of the work on teleosts began at the base and worked upward rather than from the crown and downward (but see [43]). One of the initial concerns was establishing the monophyly of teleosts (see [17, 44–46]), and another was sorting out the relationships among early-branching teleost groups (i.e., osteoglossomorphs, elopomorphs, and clupeocephalans [17, 25, 45, 47, 48]), working upward through the euteleosts [49] and establishing the sequential relationships of lineages leading to the percomorphs [50–53]. By 1989, G Nelson famously summarized these efforts with the observation that although much progress to resolve the early branching patterns of the Tree of Life of fishes had been achieved, the major challenge was to resolve the problematic relationships among percomorphs: “the bush at the top” problem [54].

GD Johnson and C Patterson [51] presented an influential study with new evidence to address the percomorph problem using a then customary “exemplar” approach to survey variation and propose putative synapomorphies, rather than the standard matrix-based analyses with dense taxon sampling to optimize character states required nowadays. It is important to note that many studies addressing high-order relationships and delineation of major lineages of percomorphs based on morphological data were not based on explicit phylogenetic analyses, and hence relied mostly on authoritative summaries and synthesis of patterns of variation [55, 56]. The empirical evidence underpinning these advances was eventually compiled by EO Wiley and GD Johnson [57] through a detailed survey of the literature, producing a list of putative morphological synapomorphies for groups down to the subordinal level. They presented a classification for Actinopterygii justifying groups by evidence presented by others to support their monophyly. In doing so, EO Wiley and GD Johnson [57] “flattened” the higher teleost classification into a series of orders principally because there was no morphological evidence supporting hypotheses of relationships among those orders (the exception was the “Smegmamorpharia”, a group no longer considered monophyletic). That it is “flat” for percomorphs with a polytomy of orders is a naked acknowledgement that they lacked evidence for the relationships among these groups. The Perciformes – the largest vertebrate order, long regarded as a polyphyletic taxonomic wastebasket (e.g., [41, 42, 50, 51, 57–59]) – was circumscribed to include families not placed in other orders and tagged as a group without synapomorphies. EO Wiley and GD Johnson [57] could not create structure where no anatomical evidence for structure existed.

Starting around the mid-1970s (and before the era of internet), the most influential source for fish classification has been JS Nelson’s *Fishes of the World* [38–42], receiving more than 9300 citations (Google Scholar, as of March 2017). Another monumental effort that synthesizes knowledge on systematic ichthyology is Eschmeyer’s *Catalog of Fishes* [60], an authoritative reference for taxonomic fish names, featuring a searchable on-line database (http://www.calacademy.org/scientists/projects/catalog-of-fishes), with a print version published in 1998 [61] and a recent list of family-level names [62]. This database also indicates carefully curated valid names and their synonyms under the classification of JS Nelson’s *Fishes of the World* with modifications. It has been constantly updated since the 1980s and gradually became another obligatory reference facilitated by the pervasive influence of the internet. Only JS Nelson’s *Fishes of the World* uses explicit criteria to justify the taxonomic arrangements, while Eschmeyer’s *Catalog of Fishes* is mostly intended for nomenclatural purposes. The phylogenetic criteria used by JS Nelson to update his classifications, however, have been based mostly on his personal views of the value of morphological evidence to define phylogenetic hypotheses [63], resulting in often poorly justified combinations of previous hypotheses in order to achieve a perceived “community consensus” view of phylogeny. This tendency, to “use restraint in revising classifications and incorporate a judicious mix of the old and the new” (see foreword by L. Parent in [42]), continues in the current edition [42], featuring an eclectic mix of new molecular hypotheses and traditionally accepted yet unsupported clades (e.g., Perciformes) without explicit criteria. As noted by G Nelson [5] almost half a century ago (quoted above), ambiguous approaches in systematics are not likely to improve clarity in the ichthyological literature.

The contribution of molecular characters to establish high-order phylogenetic relationships among fishes started in the 1990s – although the importance of molecular characters was anticipated much earlier; see above quote by E Zuckerkandl and L Pauling [4] – with analyses of 28S rRNA sequences obtained via reverse transcription [64]. A significant result of these early molecular studies, summarized by G Lecointre and G Nelson [65], suggested affinities between clupeomorphs and ostariophysans (see also G Arratia [66] and GD Johnson and C Patterson [49] for morphological support). Analyses of complete mitochondrial genome sequences, starting in 1999, contributed extensively to reveal additional unanticipated affinities among lineages of fishes [67], resulting in more than 83 papers (e.g., [68–72]) reporting phylogenetic analyses of more than 1340 mitogenomic sequences between 1999 and 2014 (see also [73]). Prompted by the advent of genomics, larger sets of nuclear gene markers became available at the beginning of this century [74], opening a new window for inference of multilocus phylogenetic trees (e.g., [75–87]). Steady progress towards acquisition of larger molecular datasets via PCR and Sanger-sequencing technology in subsequent years rapidly produced multigene phylogenies (up to 20 gene fragments) that significantly improved our knowledge of fish relationships. The most recent large-scale analyses included hundreds to thousands of species across the Tree of Life of fishes [8, 10, 11, 88–90], many of which contributed to the resolution of the percomorph bush [54] into nine well-supported supra-ordinal clades (see below) [8, 27, 91, 92]. These large-scale studies also provided, for the first time, a monophyletic definition of Perciformes. Most recently, massively parallel (“next generation”) sequencing technologies, in combination with efficient methods to capture thousands of markers in a single reaction (e.g., target enrichment [93, 94]), has ushered in a promising future to tackle difficult phylogenetic questions by analyzing hundreds or thousands of gene fragments [95]. However, genome-scale comparisons among fishes based on hundreds of loci have been limited so far to studies including a few dozen [96–100] or a couple hundred fish taxa [101–103], largely supporting previous studies based on smaller number of genes (but see [101]). Compilation of genome-scale databases to enable large-scale phylogenomic studies of fishes is actively underway [104–108].

Here, we present a revised phylogenetic classification for bony fishes based on multi-locus trees inferred for nearly 2000 species. The classification is an update of the three previous versions (including two online updates posted on www.deepfin.org), originally published by R Betancur-R., RE Broughton, EO Wiley, K Carpenter, JA Lopez, C Li, NI Holcroft, D Arcila, M Sanciangco, J Cureton, et al. [8] and built on the Linnean scheme proposed by EO Wiley and GD Johnson [57]. Our phylogenetic classification has been adopted by several public databases and documentation resources, including NCBI (www.ncbi.nlm.nih.gov/Taxonomy), the Paleobiology Database (www.paleobiodb.org), FishBase (www.fishbase.org), Catalogue of Life (www.catalogueoflife.org [109]), and OneZoom (www.onezoom.org). The new version presented here incorporates phylogenetic results from recent studies and fixes involuntary errors and omissions. We also highlight and comment all cases where taxonomic decisions made by JS Nelson, T Grande and MVH Wilson [42] are in conflict with current phylogenetic hypotheses supporting this classification, as well as the differences with WN Eschmeyer [60] and R Van Der Laan, WN Eschmeyer and R Fricke [62].

## Construction and content

The phylogenetic framework for this version of the classification (version 4) is based on a recent update of the fish Tree of Life [27] with the addition of four clades obtained by large-scale phylogenetic studies: cypriniforms [102], non-cypriniform otophysans (i.e., Characiformes, Siluriformes and Gymnotiformes; [101]), percomorphs [92], and syngnatharians [103]. Input subtrees were time-scaled using the R [110] package Ape (“chronos” function [111]) and grafted to the backbone tree using custom R code (see Additional files 1 and 2) based on secondary age calibrations and functions implemented in the R package phytools [112]. The secondary calibrations were obtained from a Bayesian analysis of a subset of 201 taxa with 61 fossil age constraints (primary calibrations). Further details on phylogenetic inference, fossil calibrations, and divergence time estimates are given in the original study [8]. This study does not intend to provide a new time scale for fish evolution; instead, it provides a synthesis of our current knowledge of fish divergence times into the extended phylogenetic tree assembled herein. Shallow-level relationships and ages for many specific groups should be taken cautiously.

**Fig. 1:**
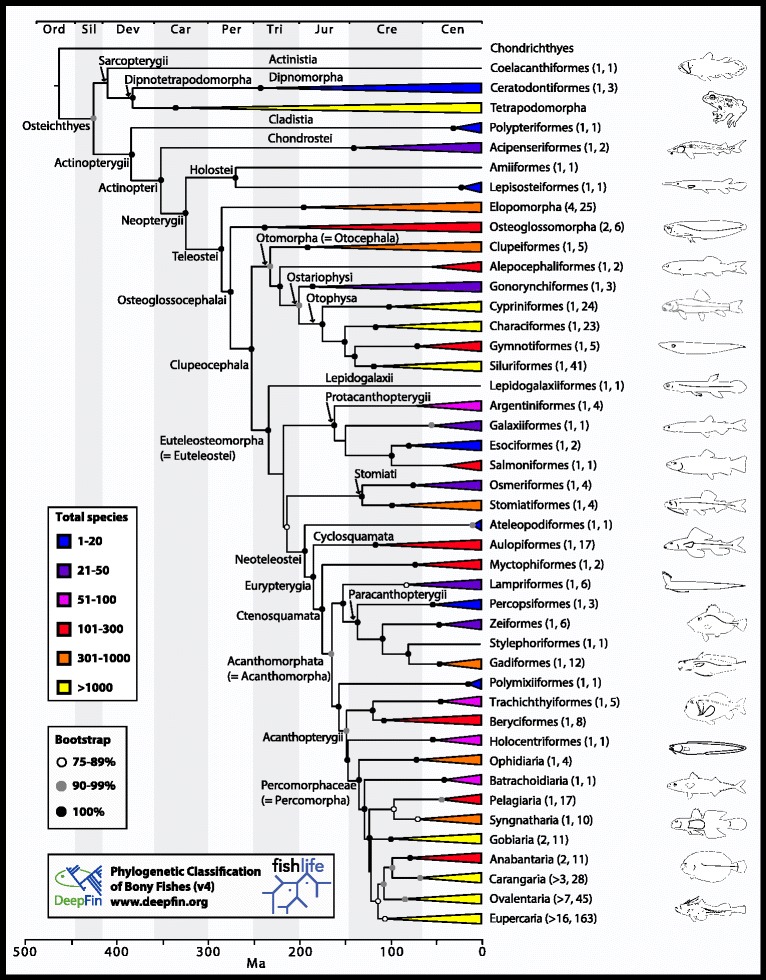
Time-calibrated Fish Tree of Life with collapsed clades that highlight the relationships of major groups (ordinal or supraordinal taxa). The backbone tree is from R Betancur-R., G Orti and AR Pyron [27], with four taxonomically-dense clades grafted (see details under “Construction and content”). The complete tree is based on 1990 species of bony fishes (see Fig. 2). Numbers in parenthesis indicate number of orders and families included in each major clade, respectively. Please see Additional file 5 for high resolution image

**Fig. 2:**
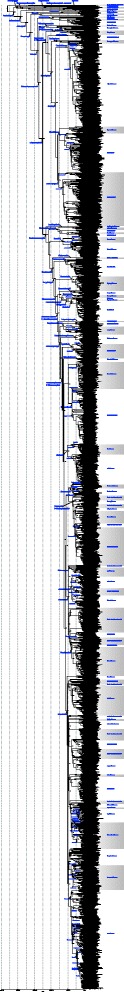
Complete time-calibrated phylogeny including 1990 species of bony fishes. Taxon labels at the tips indicate family, species name, and specimen code (Family_Genus_species_Code). The backbone tree is from R Betancur-R., G Orti and AR Pyron [27], with four taxonomically-dense clades grafted: cypriniforms [102], non-cypriniform otophysans (i.e., Characiformes, Siluriformes and Gymnotiformes; [101]), percomorphs [92], and syngnatharians [103]. Taxonomic annotations for suborders, orders and higher taxonomic groups are shown in blue. Some non-monophyletic suborders are not annotated (e.g., within Aulopiformes). Nodal numbers indicate bootstrap support values (not available for Cypriniformes or Syngnatharia, but see [102] and [103], respectively). To see details either zoom in (article PDF) or download the figure online. Please see Additional file 6 for high resolution image

The complete time tree includes 1990 species of extant bony fishes and two chondrichthyian outgroups (Figs. 1 and 2). This revision preserves names and taxonomic composition of groups presented in previous versions as much as possible; however, adjustments have been made to recognize well-supported clades, many of which have been obtained by other recent studies. Criteria for recognizing and naming clades, as in previous versions, include measures of support (bootstrap) and consistent resolution obtained by independent studies (indicated in each case). For stability purposes, we adopt some names proposed in the most recent edition of *Fishes of the World* [42] when they do not contradict our phylogeny (Fig. 2). Examples include classification of suborders in Osmeriformes, Zeiformes and Beryciformes, validation of Trachichthyiformes and recognition of Acanthopterygii. A complete list of 29 changes made in accordance with JS Nelson, T Grande and MVH Wilson [42] is presented in Additional file 3B. Many of the groups classified by JS Nelson, T Grande and MVH Wilson [42], however, are incongruent with our phylogeny and are thus not recognized. Examples of non-monophyletic taxa, as circumscribed by JS Nelson, T Grande and MVH Wilson [42], but not recognized here include Osmeromorpha, Zoroteleostei and Moroniformes. Others are recognized here, but have considerably different circumscriptions (e.g., Scombriformes, Perciformes). Tables 1 and 2 provide an exhaustive comparison of ordinal and supraordinal taxa and families that differ between this classification and JS Nelson, T Grande and MVH Wilson [42], respectively. Table 2 also lists differences with families recognized by R Van Der Laan, WN Eschmeyer and R Fricke [62].

A total of 72 orders and 79 suborders of bony fishes are classified in this version (compared to only 66 orders in version 1). For each order/suborder we list all families examined as well as the unexamined families whose inclusion is expected on the basis of traditional classifications or other phylogenetic evidence. Order-level or supraordinal taxa are herein endorsed based on well-supported clades (>90% bootstrap values) or based on clades featuring lower support in the current tree, which are otherwise consistently obtained by other studies. In some cases, order-level taxa that are not monophyletic in our analysis are also validated, provided the incongruence is not substantially rejected by our results (i.e., incongruent clades that are poorly supported in our phylogeny). The classification is presented in phylogenetic order up to the subordinal rank (following the branching order in our tree), but families within orders and suborders are listed alphabetically (including hyperlinks to FishBase; Additional file 3A only).

Family names are largely based on R Van Der Laan, WN Eschmeyer and R Fricke [62] and WN Eschmeyer and JD Fong [113], but with several exceptions (Table 2). These studies should be consulted for authorship of family names. A total of 514 families of bony fishes are now recognized (excluding tetrapods), of which 410 (~80%) are included in our large-scale phylogenetic tree (Fig. 2). The list of 104 unexamined families can be obtained from Additional file 4 (spreadsheet) that also contains the complete classification, and is intended as a resource to stimulate future phylogenetic studies. To minimize the number of non-monophyletic taxa, we have changed the membership of some traditionally recognized families whose validity is strongly challenged by phylogenetic evidence. For instance, we no longer recognize families such as Carapidae, Scaridae, Caesionidae, and Microdesmidae (lumped with Ophidiidae, Labridae, Lutjanidae, and Gobiidae, respectively). Five lineages currently recognized as separate family-level entities (“Cyclopsettidae”, “Percalatidae”, “Percophidae”, “Rivulidae” and “Pantanodontidae”) await formal nomenclatural description in compliance with the International Code of Zoological Nomenclature (ICZN). The ordinal status of 30 percomorph families (vs. 50 in version 1) included in the Series Carangaria, Ovalentaria, or Eupercaria remains uncertain due to either poor phylogenetic resolution or data unavailability. We therefore list these families as *incertae sedis* within each of these groups (Carangaria, Ovalentaria, or Eupercaria) awaiting new phylogenetic evidence to clarify their ordinal status. Twenty-three non-monophyletic families according to the framework phylogeny (Fig. 2) are recognized in this version (vs. 40 in version 1): Acropomatidae, Alepocephalidae, Bathydraconidae, Bathymasteridae, Chaenopsidae, Cheilodactylidae, Chlorophthalmidae, Clupeidae, Gempylidae, Grammatidae, Hemiramphidae, Ipnopidae, Labrisomidae, Nototheniidae, Paralepididae, Phosichthyidae, Scombridae, Scopelarchidae, Scorpaenidae, Stichaeidae, Synodontidae, Trachichthyidae, and Zenarchopteridae (see details below). Non-monophyly in these cases may be the result of poor resolution. These families are validated for stability purposes until additional evidence elucidating their status becomes available.

We cite sources for morphological synapomorphies for clades we have found in the literature. But there are caveats: (i) the original author/s may have polarized their characters using outgroups that are different than those appearing in this classification; (ii) we note that some suites of synapomorphies were meant for a more inclusive group than we recognize due to exclusion of one or more members of the previously recognized clade; and (iii) in some cases there is obvious conflict between morphological and molecular analyses. Our purpose is not to confirm these synapomorphies or to reject morphological conclusions that differ from our results. Rather, we seek to call attention to previously accomplished morphological analyses and to point out, where we can, conflicts and consilience between morphological and molecular studies, indicating groups that lack morphological support. See Additional file 3A for an indented and comment-free version of the classification.

**Table 1:** Remarkable differences for ordinal or supraordinal taxa between JS Nelson, T Grande and MVH Wilson's (NGW [42]) classification and the update proposed herein﻿.﻿ The circumscription of other orders may also differ due to variations in family validation (see Table 2) or due to inclusion of fossil taxa in NGW. Differences in taxonomic ranks and endings are considered minor and thus are not listed herein. NEL: [41]

Taxon (order-level or above)	Differences with NGW	Justification/Remarks
Teleostomi, Ginglymodi, Halecomorphi and Teleosteomorpha/Teleocephala	Not classified herein	Redundant with Osteichthyes, Amiiformes, Lepisosteiformes, and Teleostei, respectively, when only extant taxa are considered.
Dipnotetrapodomorpha	Not classified by NGW	Only shown in one of NGW’s cladograms but not formally classified therein.
Actinopteri	Not classified by NGW	Non-polypteriform actinopterygiians; a robust clade.
Elopocephalai	Not classified by NGW	Not a major difference; it is redundant with Elopomorpha.
Anguilliformes, Gadiformes	Classified into suborders in NGW but not herein	Phylogenetic incongruence with most subordinal classifications.
Cypriniformes	Classified into suborders herein but not in NGW	Following [102].
Cetopsoidei	Not classified herein	The subordinal classification for Siluriformes follows [198].
Protacanthopterygii	Includes four orders herein and only two in NGW	Differences are in part due to phylogenetic uncertainty. We classify this taxon as *sedis mutabilis*.
Zoroteleostei	Classified by NGW only	Circumscription of this taxon is in conflict with Protacanthopterygii. See comments in text.
Osmeromorpha	Classified by NGW only	Circumscription of this taxon is incongruent with all recent higher-level phylogenetic analyses of fishes. See comments in text.
Stomiati	Not classified by NGW	The circumscription of Stomiati herein is in conflict with NGW’s Osmeromorpha. See comments above and in text.
Stomiiformes/Stomiatiformes	Spelling	Stomiatiformes *sensu* [43]; Stomiiformes *sensu* [207].
Stomiatoidei/Phosichthyoidei	Phosichthyoidei *sensu* NGW; Stomiatoidei herein	Based on Stomiidae.
Paracanthopterygii	Includes Polymixiiformes in NGW but not here	*Polymixia* has a rogue placement among early acanthomorphs. Our classification is robust to phylogenetic uncertainty.
Zeiogadaria	Not classified by NGW	Denotes a robust clade including Zeiformes + (Stylephoriformes + Gadiformes); this taxon has been recognized before (i.e., Zeiogadiformes *sensu* [80]).
Berycimorphaceae/Berycida	Berycida *sensu* NGW is similar to Berycimorphaceae as classified herein, but the former includes Holocentriformes	Holocentridae is sometimes recovered as the sister taxon of percomophs, which may render Berycida *sensu* NGW non-monophyletic.
Anoplogastroidei	Not classified herein	Not monophyletic.
Trachichthyoidei	Not classified herein	Not monophyletic.
Holocentrimorphaceae	Not classified by NGW	Included in Berycida *sensu* NGW. See comments above.
Pelagiaria	Not classified by NGW	A robust clade (series) including 17 families in the order Scombriformes, as classified herein.
Scombriformes	Includes 17 families herein and only nine in NGW	Scombriformes *sensu* NGW is paraphyletic considering all higher-level molecular phylogenies of percomorphs.
Scombroidei and Stromateoidei	Not classified herein	Interfamilial resolution in Scombriformes is tenuous; classification of scombriform families into suborders requires further work.
Icosteiformes	Not classified herein	Icosteidae, the sole family in this order, is part of Pelagiaria (Scombriformes) herein.
Scombrolabraciformes	Not classified herein	Scombrolabracidae, the sole family in this order, is part of Pelagiaria (Scombriformes) herein.
Trachiniformes	Not classified herein (similar to Uranoscopiformes)	Trachiniformes *sensu* NGW is polyphyletic. It includes families placed in Pelagiaria and Eupercaria.
Syngnatharia	Not classified by NGW	A robust clade (series) including 10 families in the order Syngnathiformes, as classified herein.
Syngnathiformes	Includes 10 families herein and eight in NGW	Exclusion of Mullidae and Callionymoidei renders Syngnathiformes paraphyletic.
Aulostomoidei	Not classified herein	Not monophyletic.
Callionymiformes	Suborder (Callionymoidei) of Syngnathiformes herein	Recognition of Callionymiformes as a separate order renders Syngnathiformes paraphyletic.
Anabantaria	Not classified by NGW	A robust clade (series) including the orders Synbranchiformes (including Indostomidae) and Anabantiformes.
Indostomoidei	Not classified by NGW	Indostomidae is not included in Synbranchiformes by NGW; exclusion of this family renders the order (and component suborders) non-monophyletic.
Nandoidei	Not classified by NGW	The order Anabantiformes in classified in three suborders herein. This scheme is robust to phylogenetic uncertainity.
Carangaria	Not classified by NGW	A robust clade (series) including the orders Istiophoriformes, Carangiformes, Pleuronectiformes and several families listed as order-level *incertae sedis*.
Istiophoriformes	Includes two families herein and three in NGW	Inclusion of Sphyraenidae renders Istiophoriformes non-monophyletic.
Belonoidei/Exocoetoidei	Exocoetoidei *sensu* NGW; Belonoidei herein	Belonoidei is the name-bearer.
Eupercaria	Not classified by NGW	A robust clade (series) including at least 17 orders and 161 families. The order Perciformes belongs in this clade.
Gerreiformes	Not classified by NGW	Includes the family Gerreidae, which is sister to all other eupercarians (listed under Perciformes in NGW).
Acanthuriformes	Includes three families herein and five in NGW	Inclusion of Emmelichthyidae and Sciaenidae renders Acanthuriformes non-monophyletic.
Acanthuroidei and Sciaenoidei	Not classified herein	See comment under Acanthuriformes above.
Moroniformes/Ephippiformes	Moroniformes in NGW (three families) and Ephippiformes herein (two families)	Our results do not support a close relationship between Moronidae and Drepaneidae + Ephippidae.
Spariformes	Includes three families herein and six in NGW	Inclusion of Callanthiidae, Lobotidae (including Datnioididae) and Sillaginidae renders Spariformes non-monophyletic.
Chaetodontiformes	Not classified by NGW	A robust clade (order) including the families Chaetodontidae and Leiognathidae.
Lobotiformes	Not classified by NGW	A robust clade (order) including the families Hapalogenyidae, Datnioididae and Lobotidae (listed in Spariformes or Perciformes in NGW).
Lutjaniformes	Not classified by NGW	A robust clade (order) including the families Lutjanidae and Haemulidae (listed under Perciformes in NGW).
Priacanthiformes	Not classified by NGW	A robust clade (order) including the families Priacanthidae and Cepolidae (listed under Perciformes in NGW).
Uranoscopiformes	Not classified by NGW	A robust clade (order) including the families Ammodytidae, Cheimarrichthyidae, Pinguipedidae and Uranoscopidae (listed under Trachiniformes in NGW).
Moloidei	Not classified by NGW	Placement of Molidae in Tetraodontoidei often results in the non-monophyly of this suborder. The subordinal classification for Tetraodontiformes is robust to phylogenetic uncertainty
Triacanthodoidei	Not classified by NGW	Placement of Triacanthodidae in Triacanthoidei often results in the non-monophyly of this suborder. The subordinal classification for Tetraodontiformes is robust to phylogenetic uncertainty
Ostracioidea/Ostracioidei	Spelling	NGW recognize the “Suborder Ostracioidea”, but the appropriate ending for the rank suborder is “-iodei.”
Pempheriformes	Not classified by NGW	A robust clade (order) including 17 families (most listed under Perciformes in NGW).
Centrarchiformes	Not classified by NGW	A robust clade (order) including five suborders and 18 families (most listed under Perciformes in NGW).
Perciformes	Includes 61 families herein and 62 in NGW (but with very different circumscriptions)	Our definition of Perciformes is monophyletic; NGW maintain the *status quo* by treating Perciformes as a taxonomic waste basket (polyphyletic).
Percoidei	Includes three families herein and 46 in NGW	Our definition of Percoidei is monophyletic; NGW maintain the *status quo* by treating Percoidei as a taxonomic waste basket (polyphyletic).
Serranoidei	Not classified by NGW	Includes Serranidae.
Bembropoidei	Not classified by NGW	Includes Bembropidae.
Notothenioidei	Includes nine families herein and eight in NGW	The family Percophidae is a member of Notothenioidei herein [following 242], whereas in NGW it belongs in Trachiniformes.
Scorpaeniformes	Includes several families in NGW that are part of four different perciform suborders herein	Recognition of Scorpaeniformes as a separate order renders Perciformes non-monophyletic.
Gasterosteoidei/Gasterosteales	Gasterosteoidei (suborder of Scorpaeniformes) in NGW and Gasterosteales (suborder of Perciformes) herein	Gasterosteales herein is similar to Gasterosteoidei *sensu* NGW, except that the former excludes Indostomidae (classified under Synbranchiformes herein).
Ceratodontoidei	Not classified by NGW	Classified by NEL.
Lepidosirenoidei	Not classified by NGW	Highlights sister-group relationship between African and South American lungfishes (see also NEL).

**Table 2:** Differences in the recognition of families between JS Nelson, T Grande and MVH Wilson's (NGW [42]) and R Van Der Laan, WN Eschmeyer and R Fricke's(vdLE [62]) classifications and the update proposed herein. Taxa are listed in alphabetic order. NEL: [41]

Family	Differences with NGW	Differences with vdLE	Justification/Remarks
“Cyclopsettidae”	Provisionally recognized as “Cyclopsettidae” herein	Provisionally recognized as “Cyclopsettidae” herein	Awaits formal description; see [259]
“Pantanodontidae”	Provisionally recognized as “Pantanodontidae” herein	Provisionally recognized as “Pantanodontidae” herein	Awaits formal description; see [283]
“Percalatidae”	Provisionally recognized as “Percalatidae” herein	Provisionally recognized as “Percalatidae” herein	Awaits formal description; see text
“Percophidae”	Provisionally recognized as “Percophidae” herein	Provisionally recognized as “Percophidae” herein	Awaits formal description; lineage in Pempheriformes not related to Percophidae (Perciformes); see also [242]
“Rivulidae”	Provisionally recognized as “Rivulidae” herein; Rivulidae in NGW	_	The name Rivulidae is preoccupied in Lepidoptera (see vdLE)
Abyssocottidae	_	Synonym of Cottidae herein	Following [345]
Acheilognathidae	Subfamily of Cyprinidae in NGW	Subfamily of Cyprinidae in vdLE	Following [102]
Achiropsettidae	Synonym of Rhombosoleidae herein	Synonym of Rhombosoleidae herein	Lumped due to phylogenetic nestedness [e.g., 259]
Anotopteridae	Synonym of Paralepididae in NGW	_	Following [216]
Aphyonidae	Synonym of Bythitidae herein	Synonym of Bythitidae herein	Following [231]
Apistidae	Subfamily of Scorpaenidae in NGW	_	Following vdLE and [231]
Arapaimidae	**_**	Synomym of Osteoglossidae herein	Following [161]
Atherionidae	Synonym (subfamily) of Atherinopsidae herein	_	Following [274]
Bathygadidae	_	Synonym (subfamily) of Macrouridae in vdLE	Following [223]
Bathylaconidae	Synonym of Alepocephalidae herein	_	Following vdLE
Bathylutichthyidae	Synonym of Psychrolutidae herein	Synonym of Psychrolutidae herein	Following [345]
Bathysauropsidae	Subfamily of Ipnopidae in NGW	_	Following [216]
Bedotiidae	Subfamily of Melanotaeniidae in NGW	_	Following *.* [274] and vdLE
Bembropidae	Subfamily of Percophidae in NGW	Subfamily of Percophidae in vdLE	Following [58]
Botiidae	_	Subfamily of Cobitidae in vdLE	Following [102, 185]
Bryconidae	Subfamily of Characidae in NGW	_	Following [83, 101] and vdLE
Butidae	_	Subfamily of Eleotridae in vdLE	Following [241, 242]
Caesionidae	Synonym of Lutjanidae herein	Synonym of Lutjanidae herein	Lumped due to phylogenetic nestedness
Carapidae	Synonym of Ophidiidae herein	Synonym of Ophidiidae herein	Lumped due to phylogenetic nestedness
Centracanthidae	_	Synonym of Sparidae herein	Following [92, 298]
Chalceidae	Omitted by NGW and NEL	_	Following [83, 101] and vdLE
Cheimarrichthyidae	Spelled Cheimarrhichthyidae in NGW	_	Following vdLE
Colocongridae	Synomym of Derichthyidae in NGW	Synomym of Derichthyidae herein	Following [158]
Comephoridae	_	Synonym of Cottidae herein	Following [345]
Congrogadidae	Subfamily of Pseudochromidae in NGW	Subfamily of Pseudochromidae in vdLE	Following our results and [268]
Cottocomephoridae	_	Subfamily of Cyprinidae in NGW	Following [345]
Danionidae	Subfamily of Cyprinidae in NGW	Subfamily of Cyprinidae in vdLE	Following [102]
Datnioididae	Synonym of Lobotidae in NGW	_	Following [92]
Dinematichthyidae	Synonym of Brosmophycinae, a subfamily of Bythitidae in NGW	_	Following [231]
Dussumieriidae	_	Synonym of Clupeidae herein	Following [171]
Elassomatidae	Subfamily of Centrarchidae in NGW	_	Following vdLE and our results
Ereuniidae	_	Synonym of Rhamphocottidae herein	Following [345]
Eulophiidae	_	Synonym of Zoarcidae in vdLE	Following [344]
Gaidropsaridae	Subfamily of Gadidae in NGW	Subfamily of Lotidae in vdLE	Formerly a subfamily of Lotidae; raised to family level in version 3
Gastromyzontidae	_	Subfamily of Balitoridae in vdLE	Following [102, 186]
Girellidae	Subfamily of Kyphosidae in NGW	Subfamily of Kyphosidae in vdLE	Following our results and several recent studies [186, 321, 324, 325, 327]
Gobionellidae	_	Junior synonym of Oxudercidae	See NGW
Gobionidae	Subfamily of Cyprinidae in NGW	Subfamily of Cyprinidae in vdLE	Following [102]
Hapalogenyidae	Spelled Hapalogeniidae in NGW	_	See vdLE and [92]
Hemerocoetidae	Subfamily of Percophidae in NGW	Subfamily of Percophidae in vdLE	Following [242]
Hemitripteridae	_	Synonym of Agonidae herein	Following [345]
Iguanodectidae	Subfamily of Characidae in NGW	_	Following [83, 101]
Jordaniidae	_	Subfamily of Cottidae in vdLE	Following [345]
Kraemeriidae	_	Synonym of Gobiidae herein	Following [241, 242]
Kryptoglanidae	Synonym of Siluridae in NGW	_	Following vdLE and [345]
Latidae	Synonym of Centropomidae herein	Synonym of Centropomidae herein	Following [82, 262]
Leptobarbidae	Subfamily of Cyprinidae in NGW	Subfamily of Cyprinidae in vdLE	Following [102]
Leptobramidae	Omitted by NGW; listed in erratum	_	_
Leptochilichthyidae	_	Synomym of Alepocephalidae herein	Following [173, 174]
Lestidiidae	_	Tribe of Paralepididae in vdLE	Following [217] and NGW
Leuciscidae	Subfamily of Cyprinidae in NGW	Subfamily of Cyprinidae in vdLE	Following [102]
Lotidae	_	Synonym of Gadidae herein	Following NGW
Macroramphosidae	Synonym (subfamily) of Centriscidae herein	_	Following vdLE
Macruronidae	_	Synonym of Merlucciinae in vdLE	Following [223]
Microdesmidae	_	Synonym of Gobiidae herein	Following [241, 242]
Microcanthidae	Subfamily of Kyphosidae in NGW	Subfamily of Kyphosidae in vdLE	Following several recent studies [186, 321, 324, 325, 327]
Milyeringidae	_	Subfamily of Eleotridae in vdLE	Following [241, 242]
Neosebastidae	Subfamily of Scorpaenidae in NGW	_	Following vdLE
Niphonidae	Tribe of Serranidae in NGW	Synonym of Serranidae in vdLE	Following [58]
Notocheiridae	Subfamily of Atherinopsidae herein	Subfamily of Atherinopsidae herein	Following [274]
Odacidae	Synonym of Labridae herein	Synonym of Labridae herein	Lumped due to phylogenetic nestedness
Olyridae	**_**	Synomym of Bagridae herein	Following [198]
Omosudidae	Synonym of Alepisauridae in NGW	_	Following [216]
Ostracoberycidae	Omitted by NGW; valid in NEL	_	Following vdLE
Oxudercidae	_	Subfamily of Gobiidae in vdLE	Following [241, 242]
Paedocyprididae	Synonym of Danioninae in NGW	Synonym of Danioninae in vdLE	Following [81, 102]
Parabembridae	Synonym of Bembridae in NGW	_	Following vdLE and [357]
Parabrotulidae	Synonym of Bythitidae herein	Synonym of Bythitidae herein	Following [232]
Paralichthodidae	_	Subfamily of Pleuronectidae in vdLE	Following [265, 266]
Parascorpididae	Omitted by NGW; subfamily of Kyphosidae in NEL	_	Following vdLE
Perciliidae	Synonym of Percichthyidae herein	Synonym of Percichthyidae herein	Lumped due to phylogenetic nestedness
Perryenidae	Not recognized by NGW; *Perryena* listed under Congiopodidae	_	Following [339]
Phractolaemidae	**_**	Synomym of Kneriidae herein	Following [21]
Phycidae	Subfamily of Gadidae in NGW	_	Following [223]
Plectrogeniidae	Subfamily of Scorpaenidae in NGW	_	Following [336] and vdLE
Poecilopsettidae	_	Subfamily of Pleuronectidae in vdLE	Following [265, 266]
Polynemidae	Omitted by NGW; listed in erratum	_	_
Prototroctidae	Synonym (subfamily) of Retropinnidae herein	_	Following vdLE
Pseudomugilidae	Subfamily of Melanotaeniidae in NGW	_	Following [274] and vdLE
Psilorhynchidae	_	Synonym of Labeoninae in vdLE	Following [102]
Ranicipitidae	_	Synonym (tribe) of Gadidae in vdLE	Following [223]
Rhombosoleidae	_	Subfamily of Pleuronectidae in vdLE	Following [265, 266]
Scaridae	Synonym of Labridae herein	Synonym of Labridae herein	Lumped due to phylogenetic nestedness (e.g., [267])
Schindleriidae	_	Synonym of Gobiidae herein	Following [241, 242]
Scomberesocidae	Synonym of Belonidae herein	Synonym of Belonidae herein	Lumped due to phylogenetic nestedness (e.g., [279])
Scorpaenichthyidae	_	Subfamily of Cottidae in vdLE	Following [345]
Scorpididae	Subfamily of Kyphosidae in NGW	Subfamily of Kyphosidae in vdLE	Following several recent studies [186, 321, 324, 325, 327]
Sebastidae	Subfamily of Scorpaenidae in NGW	_	Following vdLE
Setarchidae	Subfamily of Scorpaenidae in NGW	_	Following vdLE
Sinipercidae	_	Subfamily of Percichthyidae in vdLE	Following [317]
Steindachneriidae	_	Subfamily of Merlucciidae in vdLE	Following [223]
Sudidae	_	Synonym of Paralepididae in vdLE	Following [216]
Sundadanionidae	Synonym of Danioninae in NGW	Synonym of Danioninae in vdLE	Following [102]
Sundasalangidae	**_**	Synomym of Clupeidae herein	Following [171]
Symphysanodontidae	Omitted by NGW; valid in NEL	_	Following vdLE
Synanceiidae	Subfamily of Scorpaenidae in NGW	_	Following vdLE
Tanichthyidae	Synonym of Danioninae in NGW	Synonym of Xenocypridinae in vdLE	Following [102]
Telmatherinidae	Subfamily of Melanotaeniidae in NGW	_	Following [274] and vdLE
Tetrarogidae	Subfamily of Scorpaenidae in NGW	_	Following vdLE
Tincidae	Subfamily of Cyprinidae in NGW	Subfamily of Cyprinidae in vdLE	Following [102]
Trachyrincidae	_	Synonym (subfamily) of Macrouridae in vdLE	Following [223]
Triportheidae	Synonym of Iguanodectinae, a subfamily of Characidae in NGW	_	Following [83, 101]
Xenisthmidae	_	Synonym of Eleotridae herein	Following [241, 242]
Xenocyprididae	Subfamily of Cyprinidae in NGW	Subfamily of Cyprinidae in vdLE	Following [102]
Zanclorhynchidae	Synonym of Congiopodidae in NGW	_	Following vdLE
Zaniolepididae	_	Subfamily of Hexagrammidae in vdLE	Following [345]

**Megaclass Osteichthyes** (= extant Euteleostomi)

*Morphological synapomorphies:* see G Arratia and HP Schultze [114], P Janvier [115], P Ahlberg [116], M Zhu and HP Schultze [117], M Zhu, X Yu and P Janvier [118].

**Superclass Actinopterygii** (100%)

*Morphological synapomorphies:* see C Patterson [119], MI Coates [120], H-P Schultze and SL Cumbaa [121], R Cloutier and G Arratia [122], K Mickle [123].

**Class Cladistia** (100%)

*Morphological synapomorphies:* see E Jarvik [124], R Britz and P Bartsch [125], AB Ward and NJ Kley [126].

*Comments:* polypteriforms or bichirs present a combination of characters that have led to their former identification as members of the Sarcopterygii (placed within Brachyopterygii). This view has changed since the implementation of explicit phylogenetic analyses, demonstrating that bichirs belong in Actinopterygii (e.g., [127]). Recent molecular analyses using the taxa necessary to assess the placement of bichirs (e.g., chondrichthyans, sarcopterygians and actinopterygians) have confirmed this view [8, 9].

**Order Polypteriformes**

*Morphological synapomorphies:* same as Cladistia (extant taxa only).

- Polypteridae

**Class Actinopteri** (100%)

*Morphological synapomorphies:* few morphological studies provide support for this clade; e.g., R Lund and C Poplin [128] and G-H Xu, K-Q Gao and JA Finarelli [129]. Note, however, that R Lund and C Poplin [128] did not include in their study fossil and/or extant members of chondrosteans and neopterygians. Likewise, G-H Xu, K-Q Gao and JA Finarelli [129] used in their phylogenetic analyses the *Cheirolepis* as an outgroup, not as part of the ingroup, and their coding of *Polypterus* does not consider the homologization problems that polypteriforms versus other actinopterygians present, as highlighted by R Cloutier and G Arratia [122].

*Comment*: Although morphological studies on Actinopteri are scarce, the currently accepted branching of chondrosteans, holosteans and teleosts (Fig. 1) is supported by several molecular studies (e.g., [8, 10, 11, 88–90]).

**Subclass Chondrostei** (100%)

*Morphological synapomorphies:* see G Arratia and HP Schultze [114], L Grande and WE Bemis [130], WE Bemis, EK Findeis and L Grande [131].

**Order Acipenseriformes**

*Morphological synapomorphies:* same as Chondrostei (extant taxa only).

- Acipenseridae
- Polyodontidae

**Subclass Neopterygii** (100%)

*Morphological synapomorphies:* see C Patterson and DE Rosen [47], BG Jamieson [132], L Grande [133], A López-Arbarello [134].

**Infraclass Holostei** (100%)

*Morphological synapomorphies:* L Grande [133].

*Comment*: Holostei was readopted by L Grande [133], after several decades of dismissal in ichthyology. Monophyly of Holostei has been also confirmed by several molecular studies (e.g., [8–10, 135]).

**Order Amiiformes** (= extant Halecomorphi).

*Morphological synapomorphies:* see L Grande and WE Bemis [16], G Arratia [136], G Arratia [137].

*Comment*: it should be noted that the three synapomorphies proposed by L Grande and WE Bemis [16] for amiiforms become homoplasies when other primitive teleosts, such as Triassic pholidophorids, are included in the phylogenetic analysis (see [136, 137]).

- Amiidae

**Order Lepisosteiformes** (= extant Ginglymodi) (100%)

*Morphological synapomorphies:* see EO Wiley [138], L Grande [133].

- Lepisosteidae

**Infraclass Teleostei** (100%)

*Morphological synapomorphies:* see G Arratia [17], G Arratia [136], G Arratia [46], G Arratia [137]. See also EO Wiley and GD Johnson [57].

*Comment*: Teleosteomorpha (or total group teleost including stem members), apomorphy-based Teleostei, and crown group Teleocephala in MCC de Pinna [139] are treated here as synonyms when only extant taxa are considered. However, we are aware that these three groups are diagnosed by different sets of synapomorphies (see G Arratia [46], G Arratia [137]). R Britz [140] criticism of the use of Teleosteomorpha and Teleocephala in his book review of *Fishes of the World* [42] lacks solid ground because no paleontologist or neoicthyologist is confused with the meaning of one name or the other, particularly when the concept followed is being explained. Nevertheless, we agree that the presentation of Teleocephala in JS Nelson, T Grande and MVH Wilson [42] is confusing and that the list of synapomorphies presented to support Teleocephala *sensu* MCC de Pinna [139] is a combination of three concepts.

**Megacohort Elopocephalai** *sensu* G Arratia [17] (100%).

*Morphological synapomorphies:* see G Arratia [17].

**Cohort Elopomorpha** (100%)

*Morphological synapomorphies:* see G Arratia [17], G Arratia [136]; see also comments below.

*Comments*: while divergence of Elopomorpha at the base of teleosts is counter to the prevailing view that the Osteoglossomorpha represents the earliest branching teleost lineage [36, 40, 47, 141–143], substantial morphological [17, 25, 66, 136, 144–152] and molecular [8, 9, 153, 154] evidence favors elopomorphs as the first diverging teleosts. A more recent phylogenomic analysis based on 418 orthologous genes [155] obtained support for yet another topology – a sister-group relationship between elopomorphs and osteoglossomorphs. That study, however, has a limited taxonomic scope (12 taxa), with crucial lineages that bisect long branches missing (e.g., *Hiodon*, clupeiforms and *Lepidogalaxias*). Placement of Elopomorpha as sister to the remaining teleosts is herein maintained (i.e., it is congruent with the phylogeny presented in Figs. 1 and 2).

**Order Elopiformes** (100%)

*Morphological synapomorphies:* see PL Forey [156], GD Johnson and R Britz [157].

- Elopidae
- Megalopidae

**Order Albuliformes** (95%)

*Morphological synapomorphies:* see PL Forey [156].

- Albulidae

**Order Notacanthiformes** (92%)

*Morphological synapomorphies:* see PL Forey [156].

- Halosauridae
- Notacanthidae

**Order Anguilliformes** (100%)

*Morphological synapomorphies:* see PL Forey [156], GD Johnson, H Ida, J Sakaue, T Sado, T Asahida and M Miya [158], GD Johnson and R Britz [157].

*Comment*: suborders recognized in EO Wiley and GD Johnson [57] based on previous work cited therein are significantly incongruent with the clades obtained in this analysis; thus, no subordinal classification is proposed.

- Anguillidae
- Congridae
- Eurypharyngidae
- Muraenesocidae
- Muraenidae
- Nemichthyidae
- Ophichthidae
- Saccopharyngidae
- Serrivomeridae
- *Not examined*: Chlopsidae, Cyematidae, Derichthyidae (including Colocongridae [158]), Heterenchelyidae, Monognathidae, Moringuidae, Myrocongridae, Nettastomatidae, Protanguillidae, Synaphobranchidae.

**Megacohort Osteoglossocephalai** (= Osteoglossocephala *sensu* G Arratia [17]) (100%).

*Morphological synapomorphies*: see G Arratia [17], EJ Hilton [159], J-Y Zhang [160], MVH Wilson and AM Murray [161].

**Supercohort Osteoglossomorpha** *sensu* G Arratia [17]

*Morphological synapomorphies*: see G Arratia [17], G Arratia [46], G Arratia [137]; see also comments under Elopomorpha above.

*Comments*: previous versions of the classification validated the supercohort Osteoglossocephala as well as the cohort Osteoglossomorpha, which were redundant in content. For simplicity and to avoid confusion —Osteoglossocephala *sensu* G Arratia [17] is the same as Osteoglossocephalai here and in previous versions, but not the same as Osteoglossocephala in previous versions — we now name this supercohort Osteoglossomorpha, but this change also means that the endings for the ranks cohort and supercohort are interchangeable.

**Order Hiodontiformes** (100%)

*Morphological synapomorphies:* see EJ Hilton [159], J-Y Zhang [160], MVH Wilson and AM Murray [161].

- Hiodontidae

**Order Osteoglossiformes** (42%)

*Morphological synapomorphies*: see EJ Hilton [159], J-Y Zhang [160], MVH Wilson and AM Murray [161].

*Comment*: Osteoglossidae includes *Arapaima* and *Heterotis*, formerly in Arapaimidae [161].

- Gymnarchidae
- Mormyridae
- Notopteridae
- Osteoglossidae
- Pantodontidae

**Supercohort Clupeocephala** *sensu* G Arratia [48] (100%)

*Morphological synapomorphies*: see G Arratia [48].

**Cohort Otomorpha** (= Otocephala, Ostarioclupeomorpha) (92%).

*Morphological synapomorphies*: Morphological characters supporting Otomorpha (but excluding Alepocephalidae) can be found in G Arratia [45], G Arratia [17], G Arratia [48], EO Wiley and GD Johnson [57].

*Comments*: Morphological support exists for the cohort Otomorpha, including only the subcohorts Clupei and Ostariophysi. According to G Arratia [17, 45, 48], otomorphs (her ostarioclupeomorphs) are clupeocephalans in which primitively there is an ankyloses or fusion between the mesial extrascapula and parietal alone or parietal and supraoccipital; hypurals 1 and 2 not joined by cartilage in any stage of growth, and autopalatine ossified early in ontogeny. Additionally, the presence of a modified uroneural or pleurostyle was listed as a potential synapomorphy because a pleurostyle is found in all extant otomorphs, but is absent in some of the primitive fossils of Clupei and Denticipitidae. Further research [48] re-interpreted the early ossification of the autopalatine as a clupeocephalan character, and EO Wiley and GD Johnson [57] listed a few potential synapomorphies. R Britz [140] considered the support of Otomorpha as “meagre.” To disprove the first character mentioned above, he used the condition present in advanced gonorynchiforms, the paedomorphic kneriids *Cromeria* and *Grasseichthys*, which lack parietal bones and consequently this loss represents a further transformation of the otomorph synapomorphy within the clade. The second character is questioned based on a supposedly cartilaginous connection between hypurals 1 and 2 in early developmental stages of the characiform *Salminus*, a connection that is not mentioned in the publication, but whose presence is unclear considering the unsatisfactory quality of preparation of the illustrated specimens in SMT Mattox, R Britz and M Toledo-Piza [162]. Such connection has not been described (or illustrated) in larvae of other otomorphs (see for instance [147, 163–166]).

**Subcohort Clupei** (= Clupeomorpha) (100%)

*Morphological synapomorphies*: see L Grande [167].

**Order Clupeiformes** (100%)

*Morphological synapomorphies*: same as Clupei.

**Suborder Denticipitoidei**

*Morphological synapomorphies*: L Grande [167], F Di Dario and MCC de Pinna [168], MCC de Pinna and F Di Dario [169].

- Denticipitidae

**Suborder Clupeoidei** (98%)

*Morphological synapomorphies*: L Grande [167], F Di Dario and MCC de Pinna [168], MCC de Pinna and F Di Dario [169].

*Comment*: family-level groupings may require major revision; Pristigasteridae, Chirocentridae and Engraulidae are supported by other molecular studies, but not Clupeidae [170, 171]; five well-supported lineages may become new families [171]. The family Sundasalangidae is no longer recognized because *Sundasalanx* is nested within Clupeidae (see also [172]). Clupeidae also includes the round herrings (subfamily Dussumieriinae [171]), sometimes placed in the family Dussumieriidae [62].

- Chirocentridae
- Clupeidae (not monophyletic in Fig. 2).
- Engraulidae
- Pristigasteridae

**Subcohort Alepocephali** (37%)

*Morphological synapomorphies*: see GD Johnson and C Patterson [49].

*Comments*: To the best of our knowledge, no morphological study has tested the molecular hypotheses that include the Alepocephaliformes as sister of Ostariophysi. However, as pointed out by JY Poulsen, PR Møller, S Lavoué, SW Knudsen, M Nishida and M Miya [173] “prior to the major publication of Greenwood et al. (1966), the prevailing hypothesis placed the Alepocephaliformes (with or without the Bathylaconidae) and the Clupeiformes (named Clupeoidei at this time) close to each other, within a larger group including other so-called “basal” or “primitive” teleosts, i.e., the “Isospondyli” (Berg, 1940; Bertin and Arambourg, 1958; Gosline, 1960; Marshall, 1966). Greenwood et al. (1966) tentatively transferred the Alepocephaliformes within the order Salmoniformes, only because these authors could not find any character to separate them from the Salmoniformes. However, they admitted: “there is little critical anatomical information on the Alepocephalidae, and any decision concerning their position must therefore be considered tentative… much more research is needed before the status of the Alepocephaloidei is understood.””

**Order Alepocephaliformes**

*Morphological synapomorphies*: same as Alepocephali. *Comment*: Alepocephalidae includes *Bathylaco*, placed in Bathylaconidae by JS Nelson, T Grande and MVH Wilson [42], and the former Leptochilichthyidae [173, 174]. The position of alepocephaliforms as the sister group to Ostariophysi is contrary to their more traditional placement in Euteleostomorpha (e.g., [49]). Their current placement in Otomorpha has been consistently obtained by other molecular studies (e.g., [173, 174]).

- Alepocephalidae (not monophyletic in Fig. 2).
- Platytroctidae

**Subcohort Ostariophysi** (99%)

*Morphological synapomorphies*: see SV Fink and WL Fink [175], SV Fink and WL Fink [176].

**Section Anotophysa** (= Anotophysi) (100%)

*Morphological synapomorphies*: see SV Fink and WL Fink [175], FJ Poyato-Ariza, T Grande and R Diogo [177], T Grande and FJ Poyato-Ariza [178], MP Davis, G Arratia and TM Kaiser [21].

**Order Gonorynchiformes**

*Morphological synapomorphies*: same as Anotophysa.

*Comment*: suborders in Gonorynchiformes are no longer recognized. See also JS Nelson, T Grande and MVH Wilson [42]. The former Phractolaemidae is now listed as a subfamily in Kneriidae [21].

- Gonorynchidae
- Chanidae
- Kneriidae

**Section Otophysa** (= Otophysi) (100%)

*Morphological synapomorphies*: see SV Fink and WL Fink [175], SV Fink and WL Fink [176].

*Comment*: although most molecular studies (e.g., [72, 179]) are incongruent regarding otophysan interrelationships, our recent investigation of this question using genome-wide exon data coupled with a novel method for interrogating gene genealogies [101] provides overwhelming support for the null morphological hypothesis of SV Fink and WL Fink [175], which places the monophyletic characiforms sister to a clade including siluriforms and gymnotiforms. Three otophysan superorders (Cypriniphysae, Characiphysae and Siluriphysae) are now recognized. Their taxonomic composition is similar to that originally proposed by SV Fink and WL Fink [175], except that Characiphysae now contains a single order (Characiformes) following JS Nelson, T Grande and MVH Wilson [42].

**Superorder Cypriniphysae** (92%)

*Morphological synapomorphies*: see SV Fink and WL Fink [175], SV Fink and WL Fink [176], PM Mabee, EA Grey, G Arratia, N Bogutskaya, A Boron, MM Coburn, KW Conway, S He, A Naseka, N Rios, et al. [180], KW Conway [181].

**Order Cypriniformes**

*Morphological synapomorphies*: same as Cypriniphysae.

*Comments*: recognition of suborders and families in Cypriniformes follows CC Stout, M Tan, AR Lemmon, EM Lemmon and JW Armbruster [102], which builds on WJ Chen and RL Mayden [182]. Note that the phylogenomic results by CC Stout, M Tan, AR Lemmon, EM Lemmon and JW Armbruster [102] differ from those derived from the analysis of morphological data (e.g., KW Conway [181], R Britz, K Conway and L Ruber [183]), in that the latter obtain a “Cobitoidea” *sensu* *lato* clade (including *Gyrinocheilus*, Catostomidae, and Cobitoidei *sensu stricto*), but there are relatively few characters that support that grouping and clade support is weak. This subordinal classification, with three suborders for “Cobitoidea”, is robust to phylogenetic uncertainty. Nodal support values of suborders are from CC Stout, M Tan, AR Lemmon, EM Lemmon and JW Armbruster [102].

**Suborder Gyrinocheiloidei** (100%)

*Morphological synapomorphies*: see DJ Siebert [184], KW Conway [181], PM Mabee, EA Grey, G Arratia, N Bogutskaya, A Boron, MM Coburn, KW Conway, S He, A Naseka, N Rios, et al. [180], R Britz, K Conway and L Ruber [183].

- Gyrinocheilidae

**Suborder Catostomoidei** (100%)

*Morphological synapomorphies*: see DJ Siebert [184], KW Conway [181], PM Mabee, EA Grey, G Arratia, N Bogutskaya, A Boron, MM Coburn, KW Conway, S He, A Naseka, N Rios, et al. [180], KW Conway [181], R Britz, K Conway and L Ruber [183].

- Catostomidae

**Suborder Cobitoidei** (100%)

*Morphological synapomorphies*: see KW Conway [181], R Britz, K Conway and L Ruber [183].

*Comment*: recognition of Botiidae and Gastromyzontidae follows WJ Chen, V Lheknim and RL Mayden [185] and M Kottelat [186], respectively.

- Balitoridae
- Botiidae
- Cobitidae
- Gastromyzontidae
- Nemacheilidae
- Vaillantellidae
- *Not examined*: Barbuccidae, Ellopostomatidae, Serpenticobitidae.

**Suborder Cyprinoidei** (100%)

*Morphological synapomorphies*: see KW Conway [181], R Britz, K Conway and L Ruber [183].

*Comment*: Cyprinidae *sensu* *lato* (not monophyletic) is now split into multiple monophyletic families that are coherent with biogeography. The rogue placement of *Esomus* in molecular and morphological analyses (see [102]) suggest that this genus may represent a distinct cyprinoid lineage, which is provisionally retained within Danionidae [102]. Recognition of Xenocyprididae is based on L Yang, T Sado, M Vincent Hirt, E Pasco-Viel, M Arunachalam, J Li, X Wang, J Freyhof, K Saitoh, AM Simons, et al. [187] and CC Stout, M Tan, AR Lemmon, EM Lemmon and JW Armbruster [102].

- Acheilognathidae
- Cyprinidae
- Danionidae
- Gobionidae
- Leuciscidae
- Paedocyprididae
- Sundadanionidae
- Tanichthyidae
- Xenocyprididae
- *Not examined*: Leptobarbidae, Psilorhynchidae, Tincidae.

**Superorder Characiphysae** (= Characiphysi) (100%)

*Morphological synapomorphies*: those listed for Characiformes in SV Fink and WL Fink [175], SV Fink and WL Fink [176], RP Vari [188].

*Comment*: circumscription of Characiphysae here and in JS Nelson, T Grande and MVH Wilson [42] differs from that of SV Fink and WL Fink [175]; see comment under Otophysa above.

**Order Characiformes**

*Morphological synapomorphies*: same as Characiphysae.

*Comments*: although characifom monophyly has been elusive for most molecular studies (e.g., [72, 179, 189]), our recent phylogenomic study provides overwhelming support for the monophyly of the order [101]. Nodal support values of suborders are from D Arcila, G Ortí, RP Vari, JW Armbruster, MLJ Stiassny, K Ko, MH Sabaj, J Lundberg, LJ Revell and R Betancur-R. [101].

**Suborder Citharinoidei** (100%)

*Morphological synapomorphies*: see RP Vari [190], SV Fink and WL Fink [175], SV Fink and WL Fink [176], RP Vari [188].

- Citharinidae
- Distichodontidae

**Suborder Characoidei** (100%)

*Morphological synapomorphies*: see SV Fink and WL Fink [175], SV Fink and WL Fink [176], RP Vari [188].

- Acestrorhynchidae
- Alestidae
- Anostomidae
- Chalceidae
- Characidae
- Chilodontidae
- Crenuchidae
- Ctenoluciidae
- Curimatidae
- Cynodontidae
- Erythrinidae
- Gasteropelecidae
- Hemiodontidae
- Hepsetidae
- Iguanodectidae
- Lebiasinidae
- Parodontidae
- Prochilodontidae
- Serrasalmidae
- Triportheidae
- *Not examined*: Bryconidae.

**Superorder Siluriphysae** (= Siluriphysi) (100%)

*Morphological synapomorphies*: see SV Fink and WL Fink [175], SV Fink and WL Fink [176].

**Order Gymnotiformes** (100%)

*Morphological synapomorphies*: see SV Fink and WL Fink [175], SV Fink and WL Fink [176], VA Tagliacollo, MJ Bernt, JM Craig, C Oliveira and JS Albert [191].

*Comments*: VA Tagliacollo, MJ Bernt, JM Craig, C Oliveira and JS Albert [191] proposed a revised classification for Gymnotiformes based on the most comprehensive phylogenetic analyses of the order to date, using both multi-locus sequence data and morphological evidence. They obtained two major clades within Sternopygoidei, which they named Rhamphichthyoidea (Rhamphichthyidae + Hypopomidae) and Sinusoidea (Sternopygidae + Apteronotidae). Although ranks for these clades are not explicit in their classification scheme, the endings suggest that these are superfamilies. According to the ICZN (article 61.2.2) “when a nominal taxon in the family group… is raised or lowered in rank, or its name is used at more than one rank simultaneously, the name-bearing type remains the same [Arts. 36.2, 43.1, 46.2].” In other words, the proper superfamily name for the “Sinusoidea” clade should be Sternopygoidea (suborder Sternopygoidei), to reflect a name-bearing type. Aside from these nomenclatural points, a phylogenomic-based gymnotiform clade (with a limited taxonomic sampling) does not support the monophyly of “Sinusoidea” [101]. Nodal support values of suborders are from D Arcila, G Ortí, RP Vari, JW Armbruster, MLJ Stiassny, K Ko, MH Sabaj, J Lundberg, LJ Revell and R Betancur-R. [101].

**Suborder Gymnotoidei** (100%)

*Morphological synapomorphies*: see VA Tagliacollo, MJ Bernt, JM Craig, C Oliveira and JS Albert [191].

- Gymnotidae

**Suborder Sternopygoidei** (not monophyletic here but see [191])

*Morphological synapomorphies*: see VA Tagliacollo, MJ Bernt, JM Craig, C Oliveira and JS Albert [191].

- Apteronotidae
- Hypopomidae
- Rhamphichthyidae
- Sternopygidae

**Order Siluriformes** (100%)

*Morphological synapomorphies*: see SV Fink and WL Fink [175], SV Fink and WL Fink [176], G Arratia [192], T Mo [193], G Arratia [194], MCC de Pinna [195], MCC de Pinna [196], R Diogo [197]; see also JP Sullivan, JG Lundberg and M Hardman [198].

*Comments*: recognition of catfish families follows JP Sullivan, JG Lundberg and M Hardman [198] and JG Lundberg, JP Sullivan, R Rodiles-Hernández and DA Hendrickson [77], except for Ailiidae, Auchenoglanididae and Ritidae that are herein recognized following JS Nelson, T Grande and MVH Wilson [42], and Kryptoglanidae that follows R Britz, F Kakkassery and R Raghavan [199]. The subordinal classification is based on JP Sullivan, JG Lundberg and M Hardman [198]. Nodal support values of suborders are from D Arcila, G Ortí, RP Vari, JW Armbruster, MLJ Stiassny, K Ko, MH Sabaj, J Lundberg, LJ Revell and R Betancur-R. [101].

**Suborder Loricarioidei** (75%)

*Morphological synapomorphies*: see R Diogo [197].

- Astroblepidae
- Callichthyidae
- Loricariidae
- Nematogenyidae
- Trichomycteridae
- *Not examined*: Scoloplacidae.

**Suborder Diplomystoidei**

*Morphological synapomorphies*: see G Arratia [192], G Arratia [194], MCC de Pinna [195], MCC de Pinna [196], G Arratia and C Quezada-Romegialli [200].

- Diplomystidae

**Suborder Siluroidei** (100%)

*Morphological synapomorphies*: see R Diogo [197].

*Comment*: Bagridae includes taxa formerly in Olyridae (following JP Sullivan, JG Lundberg and M Hardman [198]).

- Ailiidae
- Aspredinidae
- Auchenipteridae
- Bagridae
- Cetopsidae
- Clariidae
- Claroteidae
- Doradidae
- Heptapteridae
- Ictaluridae
- Mochokidae
- Pangasiidae
- Pimelodidae
- Plotosidae
- Pseudopimelodidae
- Siluridae
- Sisoridae
- *Not examined*: Akysidae, Amblycipitidae, Amphiliidae, Anchariidae, Ariidae, Auchenoglanididae, Austroglanididae, Chacidae, Cranoglanididae, Erethistidae, Heteropneustidae, Horabagridae, Kryptoglanidae, Lacantuniidae, Malapteruridae, Ritidae, and Schilbeidae.

**Cohort Euteleosteomorpha** (= Euteleostei *sensu* GD Johnson and C Patterson [49]) (100%).

*Morphological synapomorphies*: see GD Johnson and C Patterson [49].

*Comments*: while relationships among major euteleost lineages are contentious (e.g., Protacanthopterygii; see below), many unexpected clades classified here are consistently obtained by other molecular studies. For instance, alepocephalids have affinities within Otomorpha, instead of Argentiformes as proposed by GD Johnson and C Patterson [49] (e.g., [173]; see also comments under Alepocephali above); *Lepidogalaxias* (order Lepidogalaxiiformes) lies at the base of the euteleost tree (e.g., [201]), rendering Galaxiidae *sensu* *lato* non-monophyletic; Osmeriformes (considered a suborder of Salmoniformes by EO Wiley and GD Johnson [57]) and Stomiatiformes are sister orders (see also [76]), placed here in the subcohort Stomiati.

**Subcohort Lepidogalaxii**

*Morphological synapomorphies*: see DE Rosen [202].

**Order Lepidogalaxiiformes**

*Morphological synapomorphies*: same as Lepidogalaxii.

- Lepidogalaxiidae

**Subcohort Protacanthopterygii** *sedis mutabilis* (100%)

*Comments*: Circumscription of Protacanthopterygii is controversial (hence *sedis mutabilis*). JS Nelson, T Grande and MVH Wilson [42] restricted Protacanthopterygii to the clade including Salmoniformes and Esociformes. They also placed the orders Galaxiiformes and Argentiniformes, along with Stomiatiformes and Osmeriformes, in a new taxon they named Osmeromorpha. Circumscription of Osmeromorpha follows the results of the molecular phylogeny of CP Burridge, RM McDowall, D Craw, MVH Wilson and JM Waters [203]. Note that Burridge et al.’s study was designed to address intrafamilial galaxiid relationships. Their selection of non-galaxiid outgroups was only for time-calibration purposes; they did not intend to assess supraordinal relationships among early euteleosts. In addition to Osmeromorpha, JS Nelson, T Grande and MVH Wilson [42] classified a purported clade including most euteleosts, except for Lepidogalaxiiformes, Salmoniformes and Esociformes, in an unranked taxon named Zoroteleostei by MVH Wilson and RG Williams [204]. Circumscriptions of Osmeromorpha and Zoroteleostei *sensu* JS Nelson, T Grande and MVH Wilson [42] are incongruent with all recent higher-level phylogenetic analyses of fishes (i.e., [8–10, 27]).

**Order Argentiniformes** (47%)

*Morphological synapomorphies*: see GD Johnson and C Patterson [49].

- Argentinidae
- Bathylagidae
- Microstomatidae
- Opisthoproctidae

**Order Galaxiiformes** (94%)

*Morphological synapomorphies*: lacking.

- Galaxiidae

**Order Salmoniformes** (62%)

*Morphological synapomorphies*: see CJ Sanford [205], GD Johnson and C Patterson [49], [206].

- Salmonidae

**Order Esociformes** (100%)

*Morphological synapomorphies*: see GD Johnson and C Patterson [49].

- Esocidae
- Umbridae

**Subcohort Stomiati** (100%)

*Morphological synapomorphies*: lacking

*Comments*: see comments under Protacanthopterygii above.

**Order Stomiatiformes** *sensu* DE Rosen [43] (= Stomiiformes *sensu* WL Fink and SH Weitzman [207]) (100%)

*Morphological synapomorphies*: see AS Harold and SH Weitzman [208], AS Harold [209].

*Comments*: suborders in Stomiatiformes are now recognized following JS Nelson, T Grande and MVH Wilson [42], except that their Phosichthyoidei is named Stomiatoidei herein (based on Stomiidae).

**Suborder Gonostomatoidei** (54%)

*Morphological synapomorphies*: see AS Harold [209].

*Comment*: Diplophidae is no longer recognized as a separate family; it is listed as subfamily of Gonostomatidae in R Van Der Laan, WN Eschmeyer and R Fricke [62] and JS Nelson, T Grande and MVH Wilson [42]. *Diplophos* is sister to all other gonostomatids in Fig. 2.

- Gonostomatidae

**Suborder Stomiatoidei** (= Phosichthyoidei) (61%)

*Morphological synapomorphies*: lacking.

- Phosichthyidae (not monophyletic in Fig. 2).
- Sternoptychidae
- Stomiidae

**Order Osmeriformes** (100%)

*Morphological synapomorphies*: Formal diagnosis of the present order is not established on synapomorphies. This concept conflicts with the morphological hypothesis of GD Johnson and C Patterson [49] who grouped retropinnids with galaxiids and lepidogalaxiids.

*Comments*: EO Wiley and GD Johnson [57], citing GD Johnson and C Patterson [49], placed Galaxiidae as sister to retropinnids within the suborder Osmeroidei (order Salmoniformes *sensu* EO Wiley and GD Johnson [57]). Suborders in Osmeriformes are now classified following JS Nelson, T Grande and MVH Wilson [42].

**Suborder Osmeroidei** (100%)

*Morphological synapomorphies*: GD Johnson and C Patterson [49].

- Osmeridae
- Plecoglossidae
- Salangidae

**Suborder Retropinnoidei** (100%)

*Morphological synapomorphies*: GD Johnson and C Patterson [49].

*Comment*: Retropinnidae includes the former Prototroctidae, following JS Nelson, T Grande and MVH Wilson [42].

- Retropinnidae

**Subcohort Neoteleostei** (100%)

*Morphological synapomorphies*: see DE Rosen [43], GD Johnson [210], EO Wiley and GD Johnson [57]. Note that previous classifications (e.g., [57]) included Stomiiformes in Neoteleostei.

**Infracohort Ateleopodia** (= Ateleopodomorpha) (98%).

*Morphological synapomorphies*: see DE Rosen [43], JE Olney, DG Johnson and CC Baldwin [211].

**Order Ateleopodiformes**

*Morphological synapomorphies*: same as Ateleopodia.

- Ateleopodidae

**Infracohort Eurypterygia** (= Eurypterygii) (96%)

*Morphological synapomorphies*: see GV Lauder and KF Liem [36], GD Johnson [210], MLJ Stiassny [212], VG Springer and DG Johnson [213].

**Section Cyclosquamata** (= Aulopa) (100%)

*Morphological synapomorphies*: see C Baldwin and GD Johnson [214], TP Satoh and T Nakabo [215], MP Davis [216].

*Comment*: We now recognize Cyclosquamata *sensu* Rosen following other recent classifications (e.g., [42, 216]; = Aulopa in EO Wiley and GD Johnson [57] and in previous versions of this classification).

**Order Aulopiformes** (100%)

*Morphological synapomorphies*: same as Cyclosquamata.

*Comment*: although not monophyletic herein, the monophyly of aulopiform suborders is supported by MP Davis [216]. Aulopiform families listed follow MP Davis [216] and other recent sources (see below).

**Suborder Aulopoidei** (not monophyletic in Fig. 2) (= Synodontoidei *sensu* C Baldwin and GD Johnson [214] and EO Wiley and GD Johnson [57]).

*Morphological synapomorphies*: see C Baldwin and GD Johnson [214], MP Davis [216].

- Aulopidae
- Pseudotrichonotidae
- Synodontidae (not monophyletic in Fig. 2).

**Suborder Paraulopoidei**

*Morphological synapomorphies*: see MP Davis [216].

- Paraulopidae

**Suborder Alepisauroidei** (not monophyletic in Fig. 2)

*Morphological synapomorphies*: see MP Davis [216].

*Comments*: Alepisauridae includes taxa previously listed in Omosudidae and Anotopteridae, following MP Davis [216]. Lestidiidae is now recognized following MJ Ghedotti, RW Barton, AM Simons and MP Davis [217] and JS Nelson, T Grande and MVH Wilson [42].

- Alepisauridae
- Bathysauridae
- Chlorophthalmidae (not monophyletic in Fig. 2)
- Evermannellidae
- Giganturidae
- Ipnopidae (not monophyletic in Fig. 2)
- Lestidiidae
- Notosudidae
- Paralepididae (not monophyletic in Fig. 2)
- Scopelarchidae (not monophyletic in Fig. 2)
- Sudidae (following [216])
- *Not examined*: Bathysauroididae, Bathysauropsidae *sensu* MP Davis [216].

**Section Ctenosquamata** *sensu* DE Rosen [43] (100%)

*Morphological synapomorphies*: see GD Johnson [210], MLJ Stiassny [212].

**Subsection Myctophata** (= Scopelomorpha) (100%)

*Morphological synapomorphies*: see MLJ Stiassny [212], VG Springer and DG Johnson [213].

**Order Myctophiformes**

*Morphological synapomorphies*: same as Myctophata.

- Myctophidae
- Neoscopelidae

**Subsection Acanthomorphata** (= Acanthomorpha) (96%)

*Morphological synapomorphies*: see MLJ Stiassny [218], GD Johnson and C Patterson [51], D Davesne, C Gallut, V Barriel, P Janvier, G Lecointre and O Otero [24].

**Division Lampripterygii** (= Lampridacea in previous versions; = Lamprimorpha in [42]) (82%).

*Morphological synapomorphies*: see JE Olney, DG Johnson and CC Baldwin [211] (but including *Stylephorus*, now in Stylephoriformes; see below), D Davesne, M Friedman, V Barriel, G Lecointre, P Janvier, C Gallut and O Otero [219].

*Comments*: Endings for the rank “division” have been changed to “-pterygii” (see comments under Acanthopterygii below).

**Order Lampriformes** (= Lampridiformes in previous versions, = Allotriognathi).

*Morphological synapomorphies*: same as Lampripterygii.

- Lampridae (= Lamprididae in previous versions).
- Lophotidae
- Regalecidae
- Trachipteridae
- *Not examined*: Radiicephalidae, Veliferidae.

**Division Paracanthopterygii** *sensu* M Miya, T Satoh and M Nishida [69], T Grande, WC Borden and WL Smith [220] (but excluding Polymixiidae; = Paracanthomorphacea in previous versions) (100%).

*Morphological synapomorphies*: see T Grande, WC Borden and WL Smith [220], but restricted to our concept of the clade (without *Polymixia*).

*Comments*: endings for the rank Division have been changed to “-pterygii” (see comments under Acanthopterygii below). Placement of Polymixiidae inside [69, 106, 220] or outside [8, 10, 11, 27] Paracanthopterygii is contentious. A restricted circumscription of Paracanthopterygii, including only the orders Percopsiformes, Zeiformes, Stylephoriformes, and Gadiformes is largely robust to phylogenetic uncertainty.

**Series Percopsaria** (100%)

*Morphological synapomorphies*: see VG Springer and DG Johnson [213], T Grande, WC Borden and WL Smith [220], D Davesne, C Gallut, V Barriel, P Janvier, G Lecointre and O Otero [24].

**Order Percopsiformes**

*Morphological synapomorphies*: same as Percopsaria.

- Amblyopsidae
- Aphredoderidae
- Percopsidae

**Series Zeiogadaria** (= Zeiogadiformes *sensu* B Li, A Dettai, C Cruaud, A Couloux, M Desoutter-Meniger and G Lecointre [80]) (100%)

*Morphological synapomorphies*: see D Davesne, C Gallut, V Barriel, P Janvier, G Lecointre and O Otero [24].

**Subseries Zeiariae** (100%)

*Morphological synapomorphies*: see GD Johnson and C Patterson [51], JC Tyler, B O’Toole and R Winterbottom [221], D Davesne, C Gallut, V Barriel, P Janvier, G Lecointre and O Otero [24].

**Order Zeiformes**

*Morphological synapomorphies*: same as Zeiariae.

*Comment*: Zeiform suborders are now classified following JC Tyler, B O’Toole and R Winterbottom [221] and JS Nelson, T Grande and MVH Wilson [42].

**Suborder Cyttoidei**

*Morphological synapomorphies*: see JC Tyler, B O’Toole and R Winterbottom [221].

- *Not examined*: Cyttidae.

**Suborder Zeiodei**

*Morphological synapomorphies*: see JC Tyler, B O’Toole and R Winterbottom [221].

- Parazenidae
- Zeidae
- Zeniontidae (= Zenionidae)
- *Not examined*: Grammicolepididae, Oreosomatidae.

**Subseries Gadariae** (100%)

*Morphological synapomorphies*: lacking; note that morphology unites Stylephoriformes with Zeiformes to the exclusion of Gadiformes [24].

**Order Stylephoriformes** *sensu* M Miya, NI Holcroft, TP Satoh, M Yamaguchi, M Nishida and EO Wiley [70].

*Morphological synapomorphies*: see JE Olney, DG Johnson and CC Baldwin [211].

*Comment*: Removal of Stylephoridae from Lampriformes is well supported by molecular evidence [8, 10, 70].

- Stylephoridae

**Order Gadiformes** (100%)

*Morphological synapomorphies*: see H Endo [222].

*Comments*: the classification of suborders and families in Gadiformes is controversial (see discussion in A Roa-Varon and G Orti ([223]: Fig. 6) and recent results by M Malmstrøm, M Matschiner, OK Tørresen, B Star, LG Snipen, TF Hansen, HT Baalsrud, AJ Nederbragt, R Hanel, W Salzburger, et al. [106]). Until further evidence for resolution of relationships among families becomes available, we refrain from classifying suborders and list all families alphabetically. The family Lotidae is no longer recognized here because it is not monophyletic (see also [106]); the three genera (*Brosme*, *Lota*, and *Molva*) formerly in Lotidae are now included in Gadidae (see also JS Nelson, T Grande and MVH Wilson [42]). The families Bathygadidae, Macruronidae, Ranicipitidae, and Trachyrincidae (not validated in previous versions) are now recognized following JS Nelson, T Grande and MVH Wilson [42].

- Bathygadidae
- Gadidae
- Gaidropsaridae
- Macrouridae
- Macruronidae
- Merlucciidae
- Moridae
- Muraenolepididae
- Phycidae
- Steindachneriidae
- *Not examined*: Bregmacerotidae, Euclichthyidae, Melanonidae, Ranicipitidae, Trachyrincidae.

**Division Polymixiipterygii** (100%)

*Morphological synapomorphies*: see MLJ Stiassny [218], D Davesne, C Gallut, V Barriel, P Janvier, G Lecointre and O Otero [24].

*Comments*: endings for the rank Division have been changed to “-pterygii” (see comments under Acanthopterygii below). We place Polymixiidae in its own division (as opposed to Paracanthopterygii as in previous studies [69, 106, 220]) to recognize its rogue placement among early acanthomorph lineages. See also comments above under Paracanthopterygii.

**Order Polymixiiformes**

*Morphological synapomorphies*: same as Polymixiipterygii.

- Polymixiidae

**Division Acanthopterygii** (= Euacanthomorphacea in previous versions) (95%)

*Morphological synapomorphies*: see MLJ Stiassny and JA Moore [52], GD Johnson and C Patterson [51], EO Wiley and GD Johnson [57] (but their circumscription of the group includes Zeiformes).

*Comment*: previous versions of this classification named this clade Euacanthomorphacea, a taxon recognized by GD Johnson and C Patterson [51] to include polymixiids, percopsids and crown acanthomorphs. Because polymixiids and percopsids are not members of this group, it seems reasonable to instead adopt Acanthopterygii, recognizing its extensive use in ichthyology. Note that Acanthopterygii was listed but not classified by EO Wiley and GD Johnson [57]. This change follows TJ Near, A Dornburg, RI Eytan, BP Keck, WL Smith, KL Kuhn, JA Moore, SA Price, FT Burbrink, M Friedman, et al. [11] and JS Nelson, T Grande and MVH Wilson [42]. For consistency, we also changed all Division suffixes to “-pterygii.”

**Subdivision Berycimorphaceae** (100%)

*Morphological synapomorphies*: lacking for the entire group. A subgroup comprised of berycoids, trachichthyiforms and holocentriforms, but excluding stephanoberycoids has been recognized by presence of the Jakubowski’s organ and the absence of pharyngobranchial 4 [51]. This subgroup plus the zeiforms was also united by GD Johnson and C Patterson [51] with Percomorphaceae based on three hypothesized synapomorphies. These authors also hypothesized a sister-group relationship between Beryciformes (minus stephanoberycoids) and Percomorphaceae (forming the Euacanthopterygii) based on five other synapomorphies (see [57]). More recently, D Davesne, C Gallut, V Barriel, P Janvier, G Lecointre and O Otero [24] recognized holocentriforms as the sister to Percomorphaceae, as proposed originally by MLJ Stiassny and JA Moore [52]. We conclude that no current diagnosis based on morphological synapomorphies exists for this clade.

*Comments*: Beryciformes *sensu* *lato* (as in previous versions) is now split into Beryciformes *sensu stricto* (including Berycoidei and Stephanoberycoidei) and Trachichthyiformes *sensu* JA Moore [53], following JS Nelson, T Grande and MVH Wilson [42].

**Order Beryciformes** (100%)

*Morphological synapomorphies*: lacking for current circumscription; see JA Moore [53] and MLJ Stiassny and JA Moore [52].

*Comment*: beryciform suborders are now classified following JS Nelson, T Grande and MVH Wilson [42].

**Suborder Berycoidei** (100%)

*Morphological synapomorphies*: lacking for current circumscription; see JA Moore [53] and MLJ Stiassny and JA Moore [52].

- Berycidae
- Melamphaidae

**Suborder Stephanoberycoidei** (78%)

*Morphological synapomorphies*: see GD Johnson and C Patterson [49], JA Moore [53]. Note that GD Johnson and C Patterson [49] did not consider stephanoberycoids closely related to other beryciforms and suggested that JA Moore [53]‘s synapomorphies were ambiguous.

- Barbourisiidae
- Cetomimidae
- Rondeletiidae
- Stephanoberycidae
- *Not examined*: Gibberichthyidae, Hispidoberycidae.

**Order Trachichthyiformes** *sensu* JA Moore [53] (100%)

*Morphological synapomorphies*: see JA Moore [53] and C Baldwin and GD Johnson [224].

*Comment*: The subordinal classification for Trachichthyiformes proposed by JS Nelson, T Grande and MVH Wilson [42] is incongruent with the phylogeny in Fig. 2 and is therefore not implemented herein.

- Anomalopidae
- Anoplogastridae
- Diretmidae
- Monocentridae
- Trachichthyidae (not monophyletic in Fig. 2)

**Subdivision Holocentrimorphaceae** (100%)

*Morphological synapomorphies*: MLJ Stiassny and JA Moore [52] and JA Moore [53] provided morphological evidence supporting a sister-group relationship between holocentrids and percomorphs, validating the placement of this family in its own order (but see [225, 226]). See also D Davesne, C Gallut, V Barriel, P Janvier, G Lecointre and O Otero [24].

**Order Holocentriformes**

*Morphological synapomorphies*: same as Holocentrimorphaceae.

- Holocentridae

**Subdivision Percomorphaceae** (= Percomorpha *sensu* M Miya, H Takeshima, H Endo, N Ishiguro, J Inoue, T Mukai, T Satoh, M Yamaguchi, A Kawaguchi, K Mabuchi, et al. [68], and M Miya, T Satoh and M Nishida [69]).

*Morphological synapomorphies*: see GD Johnson and C Patterson [49], EO Wiley and GD Johnson [57].

*Comments*: the diversity of percomorph fishes (>17,000 species) is grouped into nine well-supported series (supraordinal groups). See comments in the Introduction.

**Series Ophidiaria** (100%)

*Morphological synapomorphies*: a cranial ophidiiform synapomorphy was recently proposed by G Carnevale and D Johnson [227]. Although monophyly of this group is robust from a molecular perspective, evidence from other anatomical studies is rather weak (e.g., [35, 57, 228, 229]).

**Order Ophidiiformes**

*Morphological synapomorphies*: same as Ophidiaria.

**Suborder Ophidioidei** (100%)

*Morphological synapomorphies*: lacking; see JG Nielsen, Cohen, D. M., Markle, D. F. & Robins, C. R. [229], EO Wiley and GD Johnson [57].

Ophidiidae (includes the former Carapidae).

**Suborder Bythitoidei** (100%)

*Morphological synapomorphies*: see C Patterson and D Rosen [230] and JG Nielsen, Cohen, D. M., Markle, D. F. & Robins, C. R. [229].

*Comments*: Carapidae is now synonymized with Ophidiidae due to phylogenetic nestedness. Recognition of Dinematichthyidae follows PR Møller, SW Knudsen, W Schwarzhans and JG Nielsen [231]; raised from subfamily Dinematichthyinae (formerly Bythitidae). These authors also lumped Aphyonidae with Bythitidae; thus, Aphyonidae is no longer validated. Finally, Parabrotulidae is also now synonymized with Bythitidae based on recent results by MA Campbell, JG Nielsen, T Sado, C Shinzato, M Kanda, TP Satoh and M Miya [232].

- Dinematichthyidae
- Bythitidae (includes the former Aphyonidae and Parabrotulidae).

**Series Batrachoidaria** (100%)

*Morphological synapomorphies*: see DW Greenfield, R Winterbottom and BB Collette [233], EO Wiley and GD Johnson [57] (references therein).

**Order Batrachoidiformes**

*Morphological synapomorphies*: same as Batrachoidaria.

- Batrachoididae

**Series Pelagiaria** (= Stromateoidei *sensu* B Li, A Dettai, C Cruaud, A Couloux, M Desoutter-Meniger and G Lecointre [80]; = Pelagia *sensu* M Miya, M Friedman, TP Satoh, H Takeshima, T Sado, W Iwasaki, Y Yamanoue, M Nakatani, K Mabuchi, JG Inoue, et al. [234]) (99%).

*Morphological synapomorphies*: lacking. The diagnosis provided by EO Wiley and GD Johnson [57], based on GD Johnson [235], includes families placed outside this clade in Fig. 2 (e.g., Istiophoridae). The circumscription of Scombriformes presented here is also incongruent with that of BB Collette, T Potthoff, WJ Richards, S Ueyanagi, JL Russo and Y Nishikawa [236] and other studies cited by EO Wiley and GD Johnson [57]. No morphological diagnosis exists for pelagiarians, representing a case of significant incongruence between morphological and molecular data. Despite the disparate morphology among members of Scombriformes, most are offshore fishes that inhabit pelagic environments (hence the clade name).

**Order Scombriformes**

*Morphological synapomorphies*: same as Pelagiaria.

*Comment*: interfamilial resolution in Scombriformes is tenuous; classification of scombriform families into suborders (e.g., Scombroidei, Stromateoidei, Icosteoidei) or new orders requires further work. Our circumscription of Scombriformes includes taxa placed by JS Nelson, T Grande and MVH Wilson [42] in the orders Scombriformes, Trachiniformes in part, Icosteiformes and Scombrolabraciformes.

- Ariommatidae
- Arripidae
- Bramidae
- Caristiidae
- Centrolophidae
- Chiasmodontidae
- Gempylidae (not monophyletic in Fig. 2)
- Icosteidae
- Nomeidae
- Pomatomidae
- Scombridae (not monophyletic here, but see [234])
- Scombrolabracidae
- Stromateidae
- Trichiuridae
- *Not examined*: Amarsipidae, Scombropidae, Tetragonuridae (see [234, 237]).

**Series Syngnatharia** (84%)

*Morphological synapomorphies*: lacking; no morphological character seems to unite some disparate groups (e.g., mullids) with other members of this clade (e.g., syngnathids).

*Comment*: Nodal support values of suborders are from SJ Longo, BC Faircloth, A Meyer, MW Westneat, ME Alfaro and PC Wainwright [103].

**Order Syngnathiformes** (see also [103, 238])

*Morphological synapomorphies*: same as Syngnatharia.

**Suborder Syngnathoidei** (100%)

*Morphological synapomorphies*: EO Wiley and GD Johnson [57] diagnosis included the family Pegasidae, now placed in Dactylopteroidei.

- Aulostomidae
- Centriscidae (including taxa often placed in Macroramphosidae)
- Fistulariidae
- Solenostomidae
- Syngnathidae

**Suborder Dactylopteroidei** (>92%; see [103])

*Morphological synapomorphies*: sea moths (pegasids) and flying gurnards (dactylopterids) share the condition of fused nasals in adults [57] – a possible synapomorphy.

- Dactylopteridae
- Pegasidae

**Suborder Callionymoidei** (= Callionymiformes *sensu* JS Nelson, T Grande and MVH Wilson [42]) (100%)

*Morphological synapomorphies*: see EO Wiley and GD Johnson [57], citing WA Gosline [239], who grouped callionymoids with Gobiesocoidei in the order Gobiesociformes.

- Callionymidae
- *Not examined*: Draconettidae (assumed affinity with Callionymidae).

**Suborder Mulloidei** (100%)

*Morphological synapomorphies*: see B-J Kim [240].

- Mullidae

**Series Gobiaria** (= Gobiiformes *sensu* CE Thacker [241], and CE Thacker, TP Satoh, E Katayama, RC Harrington, RI Eytan and TJ Near [242]) (100%)

*Morphological synapomorphies*: CE Thacker [241], CE Thacker [241], and CE Thacker, TP Satoh, E Katayama, RC Harrington, RI Eytan and TJ Near [242].

**Order Kurtiformes** (= Apogonoidei *sensu* CE Thacker, TP Satoh, E Katayama, RC Harrington, RI Eytan and TJ Near [242]) (100%)

*Morphological synapomorphies*: GD Johnson [50] noted that the configuration of dorsal gill-arch elements and sensory papillae may be homologous in *Kurtus* and apogonids (see also [241]).

**Suborder Kurtoidei** (100%)

*Morphological synapomorphies*: see TM Berra [243].

- Kurtidae

**Suborder Apogonoidei** (100%)

*Morphological synapomorphies*: see C Baldwin and GD Johnson [244].

- Apogonidae

**Order Gobiiformes** (100%) (=Trichonotoidei *sensu* CE Thacker, TP Satoh, E Katayama, RC Harrington, RI Eytan and TJ Near [242])

*Morphological synapomorphies*: lacking for current circumscription, but see discussion in CE Thacker [241], and CE Thacker, TP Satoh, E Katayama, RC Harrington, RI Eytan and TJ Near [242].

*Comments*: The classification of suborders in Gobiiformes is now based on CE Thacker, TP Satoh, E Katayama, RC Harrington, RI Eytan and TJ Near [242], but with modifications. Our delimitation of Kurtiformes is the same as Apogonoidei in CE Thacker, TP Satoh, E Katayama, RC Harrington, RI Eytan and TJ Near [242]. We also place *Trichonotus* in its own suborder (Trichonotoidei) in Gobiiformes (see comments below); note that Trichonotoidei *sensu* CE Thacker, TP Satoh, E Katayama, RC Harrington, RI Eytan and TJ Near [242] is equivalent to Gobiiformes here. Finally, Odontobutoidei and Eleotroidei, validated in previous versions of the classification, are now considered synonyms of Gobioidei.

**Suborder Trichonotoidei**

*Morphological synapomorphies*: JS Nelson [245], DG Smith and GD Johnson [246].

*Comments*: DG Smith and GD Johnson [246] allied *Trichonotus* with two families we place in the Pempheriformes (Creediidae and Hemerocoetidae) as subfamilies of an expanded Trichonotidae on the basis of specialized configuration of the suspensorium (following JS Nelson [245]). Placement of Trichonotoide here is based on molecular evidence from CE Thacker, TP Satoh, E Katayama, RC Harrington, RI Eytan and TJ Near [242], who identified *Trichonotus* as the sister lineage of the gobies (rendering Trichonotidae *sensu* DG Smith and GD Johnson [246] polyphyletic).

- *Not examined*: Trichonotidae.

**Suborder Gobioidei** (100%)

*Morphological synapomorphies*: see R Winterbottom [247], GD Johnson and EB Brothers [248]; see also Gobiiformes in EO Wiley and GD Johnson [57].

*Comments*: recognition of Butidae, Oxudercidae and Milyeringidae follows CE Thacker [241] and CE Thacker, TP Satoh, E Katayama, RC Harrington, RI Eytan and TJ Near [242]. We now recognize Oxudercidae instead of Gobionellidae (Gobionellidae is a junior synonym). The former Microdesmidae, Kraemeriidae, Ptereleotridae, and Schindleriidae are now synonymized with Gobiidae [241, 242]. The former Xenisthmidae is now synonymized with Eleotridae [241]. Note that Schindleriidae was first recognized as a goby by GD Johnson and EB Brothers [248].

- Eleotridae
- Gobiidae
- Odontobutidae
- *Not examined*: Butidae, Milyeringidae, Oxudercidae (= Gobionellidae), Rhyacichthyidae, Thalasseleotrididae.

**Series Anabantaria** (= Anabantiformes *sensu* B Li, A Dettai, C Cruaud, A Couloux, M Desoutter-Meniger and G Lecointre [80]) (100%)

*Morphological synapomorphies*: lacking.

*Comments*: members of this group are mostly of freshwater origin and their geographic distribution is largely restricted to Africa and South East Asia (although some synbranchid species occur in Mexico and Central and South America). Most species occupy marginal, stagnant waters due to their capacity to tolerate anoxia and to obtain oxygen directly from the air.

**Order Synbranchiformes** (100%)

*Morphological synapomorphies*: lacking for current circumscription (with Indostomidae); for synapomorphies uniting Mastacembeloidei and Synbranchoidei, see RA Travers [249], GD Johnson and C Patterson [49], R Britz [250], EO Wiley and GD Johnson [57].

**Suborder Mastacembeloidei** (100%)

*Morphological synapomorphies*: see RA Travers [249], R Britz and M Kottelat [251].

- Mastacembelidae
- *Not examined*: Chaudhuriidae.

**Suborder Indostomoidei** (100%)

*Morphological synapomorphies*: R Britz and GD Johnson [252], but placed phylogenetically with Gasterosteales.

- Indostomidae

**Suborder Synbranchoidei**

*Morphological synapomorphies*: see DE Rosen and PH Greewood [253].

- Synbranchidae

**Order Anabantiformes** *sensu* R Britz [254] (= Labyrinthici) (100%)

*Morphological synapomorphies*: see R Britz [254], R Britz [255].

*Comment*: Affinities of Channidae with other anabantiform families vary among studies (e.g., [8, 11, 92]). The subordinal scheme presented with three suborders is robust to this ambiguity.

**Suborder Anabantoidei** (100%)

*Morphological synapomorphies*: see GV Lauder and KF Liem [36], R Britz [255], VG Springer and DG Johnson [213].

- Anabantidae
- Helostomatidae
- Osphronemidae

**Suborder Channoidei** (100%)

*Morphological synapomorphies*: see GV Lauder and KF Liem [36], EO Wiley and GD Johnson [57] and citations therein.

- Channidae

**Suborder Nandoidei** (91%)

*Morphological synapomorphies*: RA Collins, R Britz and L Rüber [256].

- Badidae
- Nandidae
- Pristolepididae

**Series Carangaria** (= Carangimorpha *sensu* B Li, A Dettai, C Cruaud, A Couloux, M Desoutter-Meniger and G Lecointre [80]; = Carangimorpharia in previous versions of this classification) (99%)

*Morphological synapomorphies*: in looking for possible anatomical synapomorphies uniting flatfishes, billfishes, and carangids, AG Little, SC Lougheed and CD Moyes [257] found that most taxa share a relatively low number of vertebrae, have multiple dorsal pterygiophores inserting before the second neural spine, and lack supraneurals. However, according to M Friedman [258], some of these characters are symplesiomorphies while others are absent in the remaining carangimorph groups. Despite the apparent lack of morphological synapomorphies for carangimorphs there is a strong molecular signal supporting their monophyly (e.g., [8, 11, 27, 80, 92, 100, 259–261]). Inclusion of the billfishes (Istiophoriformes) in this series represents a significant departure from previous work in morphology where most studies placed them within or sister to the scombriforms (among pelagiarians) (see [57]).

*Comment*: Centropomidae includes the former Latidae, following PH Greenwood [262] and C Li, R Betancur-R., WL Smith and G Orti [82].

**Order** ***-*** **level** ***incertae sedis*** **in Carangaria**

- Centropomidae
- Lactariidae
- Leptobramidae
- Menidae
- Polynemidae
- Sphyraenidae
- Toxotidae

**Order Istiophoriformes** (= superfamily Xiphiicae *sensu* I Nakamura [263]) (100%)

*Morphological synapomorphies*: see I Nakamura [263].

*Comment*: our tree (Fig. 2) does not support placement of Sphyraenidae in this order, as suggested by JS Nelson, T Grande and MVH Wilson [42].

- Istiophoridae
- Xiphiidae

**Order Carangiformes** (not monophyletic in Fig. 2)

*Morphological synapomorphies*: see GD Johnson [59], WF Smith-Vaniz [264].

*Comment*: monophyly of Carangiformes is not significantly rejected by the data [259].

- Carangidae
- Coryphaenidae
- Echeneidae
- Nematistiidae
- Rachycentridae

**Order Pleuronectiformes** (21%)

*Morphological synapomorphies*: see F Chapleau [265], TA Munroe [266].

*Comment*: although contentious (e.g., [261]), the monophyly of Pleuronectiformes is resolved by several molecular studies [92, 100, 259, 260].

**Suborder Psettodoidei** (100%)

*Morphological synapomorphies*: see F Chapleau [265], TA Munroe [266].

- Psettodidae

**Suborder Pleuronectoidei** (99%)

*Morphological synapomorphies*: see F Chapleau [265], TA Munroe [266].

*Comment*: Paralichthyidae is monophyletic if the *Cyclopsetta* group is included in its own family [259]. Formal description of a new family for *Cyclopsetta* is needed in compliance with the ICZN (hence “Cyclopsettidae”). Poecilopsettidae and Paralichthodidae are validated following previous work [265, 266]. Rhombosoleidae includes taxa formerly listed in Achiropsettidae [259, 265, 266].

- Achiridae
- Bothidae
- Citharidae
- Cynoglossidae
- “Cyclopsettidae” (see comments)
- Paralichthyidae
- Pleuronectidae
- Poecilopsettidae
- Rhombosoleidae
- Samaridae
- Scophthalmidae
- Soleidae
- *Not examined*: Paralichthodidae.

**Series Ovalentaria** *sensu* Smith and Near in [267] (= Stiassnyiformes *sensu* B Li, A Dettai, C Cruaud, A Couloux, M Desoutter-Meniger and G Lecointre [80]) (97%).

*Morphological synapomorphies*: lacking, but see diagnosis by Smith and Near in [267].

**Order-level** ***incertae sedis*** **in Ovalentaria**

*Comment*: Congrogadidae is validated following CM Godkin and R Winterbottom [268] (formerly a subfamily of Pseudochromidae).

- Ambassidae (= Chandidae)
- Congrogadidae
- Embiotocidae
- Grammatidae (= Grammidae; not monophyletic in Fig. 2, but see [269])
- Opistognathidae
- Plesiopidae
- Polycentridae
- Pomacentridae
- Pseudochromidae

**Superorder Cichlomorphae** (94%)

*Morphological synapomorphies*: lacking; but see PC Wainwright, WL Smith, SA Price, KL Tang, JS Sparks, LA Ferry, KL Kuhn, RI Eytan and TJ Near [267].

**Order Cichliformes**

*Morphological synapomorphies*: same as Cichlomorphae.

*Comment*: the circumscription of Cichliformes is expanded herein to include Pholidichthyidae (formerly Pholidichthyiformes [42]).

- Cichlidae
- Pholidichthyidae

**Superoder Atherinomorphae** (= Atherinomorpha *sensu* PH Greenwood, DE Rosen, SH Weitzman and GS Myers [6]) (100%)

*Morphological synapomorphies*: LR Parenti [270], VG Springer and TM Orrell [271], LR Parenti [272].

**Order Atheriniformes** (100%)

*Morphological synapomorphies*: see LR Parenti [270], BS Dyer and B Chernoff [273], LR Parenti [272].

**Suborder Atherinoidei** (100%)

*Morphological synapomorphies*: see BS Dyer and B Chernoff [273].

*Comment*: classification of suborders and families in Atheriniformes follows D Campanella, LC Hughes, PJ Unmack, DD Bloom, KR Piller and G Orti [274]; Notocheiridae is no longer recognized (subfamily of Atherinopsidae). These authors did not include *Cairnsichthys *in Melanotaeniidae, and recommend that it should be recognized as an independent lineage﻿ (potential new family).

- Atherinidae
- Bedotiidae
- Isonidae
- Melanotaeniidae
- Phallostethidae
- Pseudomugilidae
- Telmatherinidae
- *Not examined*: Atherionidae, Dentatherinidae.

**Suborder Atherinopsoidei** (100%)

*Morphological synapomorphies*: see BS Dyer and B Chernoff [273].

*Comment*: Atherinopsidae includes the subfamilies Atherinopsinae, Notocheirinae and Menidiinae. The circumscription of Atherinopsidae *sensu* JS Nelson, T Grande and MVH Wilson [42] includes only Menidiinae and Atherinopsinae, which renders Atherinopsidae non-monophyletic (Notocheirinae is nested within; see [274]).

- Atherinopsidae

**Order Beloniformes** (79%)

*Morphological synapomorphies*: see DE Rosen and LR Parenti [275], LR Parenti [272], LR Parenti [276].

**Suborder Adrianichthyoidei**

*Morphological synapomorphies*: see LR Parenti [276].

- Adrianichthyidae

**Suborder Belonoidei** (100%) (= Exocoetoidei *sensu* EO Wiley and GD Johnson [57], JS Nelson, T Grande and MVH Wilson [42]).

*Morphological synapomorphies*: see DE Rosen and LR Parenti [275], LR Parenti [277].

*Comment*: Belonidae includes the former Scomberesocidae [278, 279].

- Belonidae
- Exocoetidae
- Hemiramphidae (not monophyletic in Fig. 2)
- Zenarchopteridae (not monophyletic here, but see [279])

**Order Cyprinodontiformes** (100%)

*Morphological synapomorphies*: see DE Rosen and LR Parenti [275], LR Parenti [277].

**Suborder Aplocheiloidei**

*Morphological synapomorphies*: see LR Parenti [277], WJEM Costa [280].

*Comment*: according to R Van Der Laan, WN Eschmeyer and R Fricke [62] the name Rivulidae Myers 1925 is preoccupied by Rivulini Grote 1895 in Lepidoptera (hence "Rivulidae").

- Aplocheilidae
- *Not examined*: Nothobranchiidae, "Rivulidae" (see comments).

**Suborder Cyprinodontoidei** (100%)

*Morphological synapomorphies*: see LR Parenti [277], WJEM Costa [281].

*Comments*: Cyprinodontidae and Poeciliidae are monophyletic here, with reduced taxonomic sampling, but not in two other recent studies that included a much broader coverage [282, 283]. M Pohl, FC Milvertz, A Meyer and M Vences [283] identified a rogue placement for *Pantanodon* among cyprinodontiforms. The topology most often obtained by these authors included *Pantanodon* as sister to all cyprinodontoids. Formal description of a new family for *Pantanodon* is needed in compliance with the ICZN. The family Valenciidae is herein circumscribed to include the genus *Aphanius* (formerly in Cyprinodontidae), forming an Eurasian clade (following [283]). This revised circumscription renders Cyprinodontidae monophyletic.

- Cyprinodontidae
- Fundulidae
- Poeciliidae
- *Not examined*: Anablepidae, Goodeidae, Profundulidae, Valenciidae (includes *Aphanius*; see comments). Possibly included: “Pantanodontidae” (see comments).

**Superorder Mugilomorphae** (100%)

*Morphological synapomorphies*: see MLJ Stiassny [284], GD Johnson [50], GD Johnson and C Patterson [51].

**Order Mugiliformes**

*Morphological synapomorphies*: same as Mugilomorphae.

- Mugilidae

**Superorder Blenniimorphae** (90%)

*Morphological synapomorphies*: see H-C Lin and PA Hastings [285] (unnamed clade including gobiesocids and blennioids), VG Springer and TM Orrell [271].

*Comment*: see also PC Wainwright, WL Smith, SA Price, KL Tang, JS Sparks, LA Ferry, KL Kuhn, RI Eytan and TJ Near [267] for additional molecular evidence supporting the reciprocal monophyly of gobiesocoids and blennioids.

**Order Gobiesociformes** (100%) (= Gobiesocoidei in EO Wiley and GD Johnson [57])

*Morphological synapomorphies*: see WA Gosline [239], LR Parenti and J Song [286], EO Wiley and GD Johnson [57].

*Comment*: the order Gobiesociformes is now recognized following EO Wiley and GD Johnson [57] (but excluding Callionymoidei) and JS Nelson, T Grande and MVH Wilson [42].

- Gobiesocidae

**Order Blenniiformes** (39%) (= Blennioidei in V Springer [287])

*Morphological synapomorphies*: see V Springer [287], GD Johnson [50], RD Mooi and AC Gill [288], VG Springer and TM Orrell [271], EO Wiley and GD Johnson [57].

*Comments*: Circumscription of Blenniiformes follows H-C Lin and PA Hastings [285], based on V Springer [287] (Blennioidei). Our new tree (Fig. 2) resolves the blennioids as monophyletic, a result not obtained in our previous large-scale studies. According to H-C Lin and PA Hastings [285], Chaenopsidae is monophyletic if *Stathmonotus* is included in Labrisomidae.

- Blenniidae
- Chaenopsidae (not monophyletic in Fig. 2)
- Clinidae
- Dactyloscopidae
- Labrisomidae (not monophyletic in Fig. 2, but see [285])
- Tripterygiidae

**Series Eupercaria** (= Percomorpharia in previous versions of this classification) (83%)

*Morphological synapomorphies*: lacking.

*Comments*: with more than 6000 species arranged in 161 families and at least 17 orders (Fig. 1), Eupercaria is by far the largest series of percomorphs. Some of the most diverse orders (e.g., Perciformes, Labriformes, Lophiiformes, and Tetraodontiformes) and families (e.g., Labridae, Serranidae, and Scorpaenidae) of fishes are included in this group. Previous molecular studies obtained monophyletic groups with a combination of taxa here assigned to Eupercaria, but including far more limited sampling (e.g, [11, 58, 68, 69, 289]). Although most family-level and ordinal groups within this series receive high nodal support, interrelationships among them are largely unresolved – Eupercaria constitutes the “new bush at the top” [8]. The largest group within Eupercaria is the order Perciformes, as currently circumscribed.

**Order-level** ***incertae sedis*** **in Eupercaria.**

*Comment*: although we lack phylogenetic evidence, the family Parascorpididae, traditionally classified in “Perciformes”, is provisionally listed here; it is not placed in Perciformes, as currently circumscribed, given the long history of phylogenetic indistinctiveness between Percoidei, Perciformes, and Percomorpha [50, 51, 58]. While not examined, Dinolestidae and Dinopercidae are included here based on previous molecular work [58].

- Callanthiidae
- Centrogenyidae
- Emmelichthyidae
- Malacanthidae
- Monodactylidae
- Moronidae
- Pomacanthidae
- Scatophagidae
- Sciaenidae
- Siganidae
- Sillaginidae
- *Not examined*: Dinolestidae, Dinopercidae, Parascorpididae.

**Order Gerreiformes** (100%)

*Morphological synapomorphies*: lacking.

*Comment*: validation of Gerreiformes (Bleeker name; resurrected herein) reflects the consistent placement of Gerreidae as sister to all other eupercarians.

- Gerreidae

**Order Uranoscopiformes** (= Paratrachinoidei *sensu* B Li, A Dettai, C Cruaud, A Couloux, M Desoutter-Meniger and G Lecointre [80]) (98%).

*Morphological synapomorphies*: lacking, but see H Imamura and K Odani [290] for a review of hypotheses of relationships of the five families in this order to other members of the former suborder Trachinoidei. See also comments in EO Wiley and GD Johnson [57].

- Ammodytidae
- Cheimarrichthyidae (= Cheimarrhichthyidae)
- Pinguipedidae
- Uranoscopidae

**Order Labriformes** (100%)

*Morphological synapomorphies*: see M Stiassny and J Jensen [291] and GD Johnson [50].

Labridae (includes taxa previoulsy listed in Scaridae and Odacidae; see also [267]).

**Order Ephippiformes** (100%)

*Morphological synapomorphies*: lacking; however, PH Greenwood, DE Rosen, SH Weitzman and GS Myers [6] hypothesized a close affinity between *Drepane* and ephippids, and GD Johnson [50] cites an unpublished dissertation by Blum that provides additional morphological support.

*Comments*: JS Nelson, T Grande and MVH Wilson [42] named this clade Moroniformes, including Moronidae in addition to Drepaneidae and Ephippidae. Our results do not support the placement of Moronidae in this order.

- Drepaneidae
- Ephippidae

**Order Chaetodontiformes** (66%)

*Morphological synapomorphies*: lacking.

*Comment*: this clade has been consistently obtained by previous studies with higher nodal support than that reported here.

- Chaetodontidae
- Leiognathidae

**Order Acanthuriformes**, restricted circumscription (see also [292]) (100%)

*Morphological synapomorphies*: see JC Tyler, GD Johnson, I Nakamura and BB Collette [293].

*Comments*: JS Nelson, T Grande and MVH Wilson [42] included Emmelichthyidae and Sciaenidae in this order, in addition to Acanthuridae, Luvaridae and Zanclidae. Our results do not support the placement of Emmelichthyidae and Sciaenidae in Acanthuriformes.

- Acanthuridae
- Luvaridae
- Zanclidae

**Order Lutjaniformes,** new circumscription (59%)

*Morphological synapomorphies*: lacking.

*Comment*: the order Lutjaniformes (Bleeker name) is herein resurrected for the clade including lutjanids and haemulids. Although nodal support is low, this clade is often obtained in various large-scale studies.

- Haemulidae
- Lutjanidae (includes the former Caesionidae; e.g., [50, 294])

**Order Lobotiformes** (100%)

*Morphological synapomorphies*: lacking, but JM Leis and BM Carson-Ewart [295] suggested that *Lobotes*, *Datnioides*, and *Hapalogenys* share remarkable similarities in larval morphology. See discussion in MD Sanciangco, KE Carpenter and R Betancur-R. [92].

- Hapalogenyidae (= Hapalogeniidae)
- Datnioididae
- Lobotidae

**Order Spariformes** *sensu* M Akazaki [296] and GD Johnson [297] (87%)

*Morphological synapomorphies*: see M Akazaki [296], GD Johnson [50] and GD Johnson [297].

*Comments*: the family Centracanthidae is no longer recognized as valid; synonym of Sparidae following F Santini, G Carnevale and L Sorenson [298] and MD Sanciangco, KE Carpenter and R Betancur-R. [92]. JS Nelson, T Grande and MVH Wilson [42] also included in this order the families Callanthiidae, Lobotidae (including Datnioididae) and Sillaginidae. Our results do not support the placement of these three or four families in Spariformes.

- Lethrinidae
- Nemipteridae
- Sparidae (includes the former Centracanthidae)

**Order Priacanthiformes**, new circumscription (98%)

*Morphological synapomorphies*: lacking, but implied by unspecified larval similarities (discussed by JM Leis and BM Carson-Ewart [295]).

*Comments*: a sister-group relationship between cepolids and priacanthids is strongly supported by other molecular studies (see also [295]).

- Priacanthidae
- Cepolidae

**Order Caproiformes** (37%)

*Morphological synapomorphies*: lacking, but see F Santini and G Lecointre [299].

*Comment*: this order is herein recognized following JS Nelson, T Grande and MVH Wilson [42].

- Caproidae

**Order Lophiiformes** (100%)

*Morphological synapomorphies*: see TW Pietsch [300], TW Pietsch [301].

*Comments*: this order is the sister group of Tetraodontiformes (45% bootstrap). This relationship is also supported by anatomical evidence [302], larval characters [303], and previous molecular studies [68, 304].

**Suborder Lophioidei** (100%)

*Morphological synapomorphies*: see TW Pietsch [300].

- Lophiidae

**Suborder Antennarioidei** (100%)

*Morphological synapomorphies*: see TW Pietsch [300].

- Antennariidae

*Not examined*: Brachionichthyidae, Lophichthyidae, Tetrabrachiidae.

**Suborder Chaunacoidei** (100%)

*Morphological synapomorphies*: the suborder is unquestionably monophyletic, but a list of synapomorphies is lacking [57]; for a morphological diagnosis see TW Pietsch and DB Grobecker [305].

- Chaunacidae

**Suborder Ogcocephaloidei** (100%)

*Morphological synapomorphies*: the suborder is unquestionably monophyletic, but a list of synapomorphies is lacking [57]; for a morphological diagnosis see TW Pietsch and DB Grobecker [305].

- Ogcocephalidae

**Suborder Ceratioidei** (100%)

*Morphological synapomorphies*: T Pietsch and J Orr [306].

- Ceratiidae
- Gigantactinidae
- Himantolophidae
- Melanocetidae
- Oneirodidae
- *Not examined*: Caulophrynidae, Centrophrynidae, Diceratiidae, Linophrynidae, Neoceratiidae, Thaumatichthyidae.

**Order Tetraodontiformes** (100%)

*Morphological synapomorphies*: several studies by J. Tyler and colleagues (e.g., [307–310]). Morphological synapomorphies for suborders are implied in several recent phylogenetic analyses of fossils and extant taxa (e.g., [23, 310, 311]).

*Comments*: Although interrelationships of major tetraodontiform lineages is controversial, several clades are congruent across studies. The subordinal classification proposed here differs from that by F Santini and JC Tyler [310], with many more suborders now recognized. Our scheme is robust to phylogenetic uncertainty and has recently been adopted by AF Bannikov, JC Tyler, D Arcila and G Carnevale [311].

**Suborder Triodontoidei**

*Morphological synapomorphies*: implied in various phylogenetic analyses by J. Tyler and colleagues (e.g., [23, 310, 311]).

- Triodontidae

**Suborder Triacanthoidei**

*Morphological synapomorphies*: implied in various phylogenetic analyses by J. Tyler and colleagues (e.g., [23, 310, 311]).

- Triacanthidae

**Suborder Triacanthodoidei** (100%)

*Morphological synapomorphies*: implied in various phylogenetic analyses by J. Tyler and colleagues (e.g., [23, 310, 311]).

- Triacanthodidae

**Suborder Tetraodontoidei** (100%)

*Morphological synapomorphies*: implied in various phylogenetic analyses by J. Tyler and colleagues (e.g., [23, 310, 311]).

- Diodontidae
- Tetraodontidae

**Suborder Moloidei** (100%)

*Morphological synapomorphies*: implied in various phylogenetic analyses by J. Tyler and colleagues (e.g., [23, 310, 311]).

- Molidae

**Suborder Balistoidei** (100%)

*Morphological synapomorphies*: implied in various phylogenetic analyses by J. Tyler and colleagues (e.g., [23, 310, 311]).

- Balistidae
- Monacanthidae

**Suborder Ostracioidei** (100%)

*Morphological synapomorphies*: implied in various phylogenetic analyses by J. Tyler and colleagues (e.g., [23, 310, 311]).

- Aracanidae
- Ostraciidae

**Order Pempheriformes** (33%)

*Morphological synapomorphies*: lacking. Note that Y Tominaga [312] suggested that features of the cranium and swimbladder may be homologous in *Pempheris* and *Glaucosoma*; see also GD Johnson [50].

*Comments*: Although support for Pempheriformes is low, this clade has been obtained by several studies. Because *Percophis brasiliensis* (type species of Percophidae) is a Notothenioid [313], and the remaining “percophids” are in Pempheriformes, then the pempheriform “percophids” require family relocation. The subfamily Hemerocoetinae Kaup 1873 is now raised to the family level, following CE Thacker, TP Satoh, E Katayama, RC Harrington, RI Eytan and TJ Near [242]: “Additional proposed changes to the classification of Percomorpha include... recognition of Hemerocoetidae as a taxonomic family containing *Matsubaraea*, *Enigmapercis*, *Pteropsaron*, *Acanthaphritis*, and *Osopsaron* and the unsampled *Dactylopsaron*, *Hemerocoetes*, and *Squamicreedia.*” See comments under Order Gobiiformes for notes regarding the placement of Creediidae and Hemerocoetidae in Pempheriformes rather than Trachinoidei.

- Acropomatidae (not monophyletic in Fig. 2)
- Banjosidae
- Bathyclupeidae
- Champsodontidae
- Creediidae
- Epigonidae
- Glaucosomatidae
- Howellidae
- Lateolabracidae
- Ostracoberycidae
- Pempheridae
- Pentacerotidae
- “Percophidae” (see comments)
- Polyprionidae
- Symphysanodontidae
- *Not examined*: Hemerocoetidae, Leptoscopidae (assumed affinity with Creediidae; see [314]).

**Order Centrarchiformes** (98%)

*Morphological synapomorphies*: lacking.

*Comment*: although the family name Cirrithidae Macleay 1841 is older than Centrarchidae Bleeker 1859, we retain the name Centrarchiformes for this order (in agreement with previous usage) but expand its membership following recent proposals [11, 315, 316].

**Suborder Centrarchoidei** (93%)

*Morphological synapomorphies*: lacking.

*Comments*: inclusion of Enoploside in this suborder differs from the results obtained by S Lavoué, K Nakayama, DR Jerry, Y Yamanoue, N Yagishita, N Suzuki, M Nishida and M Miya [316]. Sinipercidae is recognized following C Li, G Orti and J Zhao [317] (formerly a synonym of Percichthyidae). As suggested by earlier classifications and confirmed by recent molecular studies (e.g., [318]), pygmy sunfishes (*Elassoma*) and sunfishes (centrarchids) are allied (placed in separate orders by EO Wiley and GD Johnson [57]).

- Centrarchidae
- Elassomatidae
- Enoplosidae
- Sinipercidae

**Suborder Cirrhitoidei** (similar to Cirrhitoidea *sensu* PH Greenwood [319], and CP Burridge and AJ Smolenski [320]; treated as Cirrhitiformes in previous versions of the classification) (97%).

*Morphological synapomorphies*: see PH Greenwood [319].

*Comment*: the families Latridae, Chironemidae and Aplodactylidae are nested within Cheilodactylidae, rendering the latter non-monophyletic [92].

- Aplodactylidae
- Cheilodactylidae (not monophyletic in Fig. 2)
- Chironemidae
- Cirrhitidae
- Latridae

**Suborder Percichthyoidei** (100%)

*Morphological synapomorphies*: GD Johnson [59], but with a different circumscription (a series of nested synapomorphies uniting all members except *Percalates*).

*Comment*: percichthyoids and Percichthyidae *sensu* GD Johnson [59] are not monophyletic: the Australian species *Percalates colonorum* and *Percalates novemaculeata* are not closely related to other members of Percichthyidae [8, 315, 316], so these species are herein placed in their own suborder [P. Unmack pers. comm.; 317]. *Percalates* is listed as a junior synonym of *Macquaria* by WN Eschmeyer [60], but the type species of *Macquaria* (*M. australasica*) is closely related to other species of *Macquaria* (*M. ambigua*) within Percichthyidae *sensu stricto*, thus both are valid genus names [P. Unmack pers. comm.; 317]. Percichthyidae *sensu stricto* includes *Percilia* (formerly placed in its own family, Perciliidae).

- Percichthyidae

**Suborder Percalatoidei** (100%)

*Morphological synapomorphies*: lacking.

*Comment*: formal description of a new family for *Percalates* is required to comply with the ICZN.

- “Percalatidae” (see comment)

**Suborder Terapontoidei** (= Clade “h2” of N Yagishita, M Miya, Y Yamanoue, SM Shirai, K Nakayama, N Suzuki, TP Satoh, K Mabuchi, M Nishida and T Nakabo [321]; = Terapontiformes in previous versions of the classification) (99%)

*Morphological synapomorphies*: lacking for current circumscription. GD Johnson and RA Fritzsche [322] cite nerve pattern evidence uniting all but one of the families listed here (Dichistiidae) plus other groups currently placed in Pelagiaria (Arripdidae and stromateoids).

*Comment*: The families Girellidae, Microcanthidae and Scorpididae are herein recognized following several recent studies [321, 323–327]; these are listed as subfamilies of Kyphosidae in R Van Der Laan, WN Eschmeyer and R Fricke [62] and JS Nelson, T Grande and MVH Wilson [42].

- Dichistiidae
- Girellidae
- Kuhliidae
- Kyphosidae
- Oplegnathidae
- Terapontidae
- *Not examined*: Microcanthidae, Scorpididae.

**Order Perciformes** (= Serraniformes *sensu* B Li, A Dettai, C Cruaud, A Couloux, M Desoutter-Meniger and G Lecointre [80], and A-C Lautredou, H Motomura, C Gallut, C Ozouf-Costaz, C Cruaud, G Lecointre and A Dettai [328]) (93%)

*Morphological synapomorphies*: lacking.

*Comments*: although Perciformes has been traditionally regarded as a “taxonomic waste basket” (e.g., [41, 42, 50, 51, 57–59]), the first version of this classification [8] proposed for the first time a monophyletic definition of the order based on robust molecular analyses. Compared to classification by other authors, the revised circumscription of Perciformes reduces significantly the number of included taxa, while retaining remarkable diversity that is now organized into several suborders and infraorders. Our definition comprises 61 perciform families, including species assigned by previous classifications to the orders Scorpaeniformes, Cottiformes, and Trachiniformes (no longer validated as orders herein).

**Suborder Bembropoidei**, new (100%)

*Morphological synapomorphies*: lacking.

*Comment*: This suborder is newly classified to accommodate the family Bembropidae. Bembropidae is recognized following WL Smith and MT Craig [58]; it is a synonym of Percophidae according to R Van Der Laan, WN Eschmeyer and R Fricke [62].

- Bembropidae

**Suborder Normanichthyoidei**

*Morphological synapomorphies*: see M Yabe, and T. Uyeno. [329].

*Comment*: this suborder is classified following R Van Der Laan, WN Eschmeyer and R Fricke [62] and JS Nelson, T Grande and MVH Wilson [42].

- *Not examined*: Normanichthyidae.

**Suborder Serranoidei** (64%)

*Morphological synapomorphies*: see GD Johnson [330], C Baldwin and GD Johnson [331], but with a different circumscription (including *Niphon*).

*Comments*: we do not recognize Epinephelidae as a separate family, following WL Smith and MT Craig [58] and KY Ma, MT Craig, JH Choat and L van Herwerden [332]. The main justification for such nomenclatural change was that Smith and Craig’s phylogenetic analysis failed to resolve the monophyly of serranids (including epinephelines, anthiines and serranines); however, they did not conduct a topology test to ask whether this null hypothesis is rejected by their data. Our large-scale analysis supports the monophyly of Serranidae (excluding *Niphon*; see comments under Percoidei below), albeit with low support. Also, while elevating Epinephelinae to family is a minor nomenclatural change, this rearrangement creates confusion for fish managers and conservation biologists given the commercial importance of groupers and the endangered status of many species.

- Serranidae

**Suborder Percoidei**, restricted circumscription (99%)

*Morphological synapomorphies*: lacking.

*Comments*: A-C Lautredou, H Motomura, C Gallut, C Ozouf-Costaz, C Cruaud, G Lecointre and A Dettai [328] obtained a clade uniting Percidae and Trachinidae with full support, based on the analysis of seven nuclear markers. Like with Perciformes, the restricted and monophyletic circumscription of Percoidei in this classification contrasts markedly with the long history of confusion regarding the limits and polyphyly of Percoidei (e.g., [50, 51, 58, 59]). Removal of *Niphon* from Serranidae (e.g., as in [330, 331]) and placement in its own family (Niphonidae) is consistent with several other studies (e.g., [58, 333]).

- Niphonidae
- Percidae
- *Not examined*: Trachinidae.

**Suborder Notothenioidei** (= Nototheniiformes in EO Wiley and GD Johnson [57]) (100%)

*Morphological synapomorphies*: see PA Hastings [334], EO Wiley and GD Johnson [57] (but with a different circumscription; see comment below).

*Comment*: Percophidae is herein placed in Notothenioidei following TJ Near, A Dornburg, RC Harrington, C Oliveira, TW Pietsch, CE Thacker, TP Satoh, E Katayama, PC Wainwright, JT Eastman, et al. [313]; see comments above under Pempheriformes.

- Artedidraconidae
- Bathydraconidae (not monophyletic here; but see [335])
- Bovichtidae
- Channichthyidae
- Eleginopsidae
- Harpagiferidae
- Nototheniidae (not monophyletic in Fig. 2)
- Pseudaphritidae
- Not ex﻿amined: Percophidae.

**Suborder Scorpaenoidei** (72%)

*Morphological synapomorphies*: lacking. Phylogenetic analysis on all or part of various scorpaenoid families (e.g., [336–338]) vary to a greater or lesser degree than the results presented here.

*Comment*: nine families now included in Scorpaenoidei were listed in previous versions of this classification as not examined under Perciformes. See also H Imamura [336].

- Scorpaenidae (not monophyletic in Fig. 2)
- Sebastidae
- Setarchidae
- Synanceiidae
- Tetrarogidae
- *Not examined*: Apistidae, Aploactinidae, Congiopodidae, Eschmeyeridae, Gnathanacanthidae, Neosebastidae, Pataecidae, Perryenidae (see [339]), Zanclorhynchidae.

**Suborder Platycephaloidei** (= Bembroidei in previous versions) (26%)

*Morphological synapomorphies*: lacking (see comments).

*Comment*: previous versions of this classification included Bembridae and Parabembridae in the suborder Bembroidei, which we now expand to also include Hoplichthyidae, Platycephalidae and Plectrogeniidae (previously listed as suborder-level *incertae sedis* in Perciformes) – a well-supported clade in our analysis (100% BS). We now name this taxon Platycephaloidei in accordance to other classifications (e.g., [336, 340]). Note that the family composition differs from that in other studies as Peristediidae and Triglidae are herein placed in a different suborder (Triglioidei).

- Bembridae
- Hoplichthyidae
- Parabembridae
- Platycephalidae
- *Not examined*: Plectrogeniidae (see [336]).

**Suborder Triglioidei** *sensu* DS Jordan [341] (100%)

*Morphological diagnosis*: SA Mandrytsa [338] presents synapomorphies; other results by H Imamura [336], and H Imamura [340] differ significantly from ours.

- Peristediidae
- Triglidae

**Suborder Cottoidei** (= Cottimorpha *sensu* Li, A Dettai, C Cruaud, A Couloux, M Desoutter-Meniger and G Lecointre [80]) (100%)

*Morphological synapomorphies*: H Imamura, S Shirai and M Yabe [342].

*Comment*: we have chosen to recognize clades within this suborder as infraorders, adopting the ending “–ales” for this rank. Gasterosteales and Zoarcales have been grouped in a clade named Zoarciformes by B Li, A Dettai, C Cruaud, A Couloux, M Desoutter-Meniger and G Lecointre [80].

**Infraorder Anoplopomatales** (= Anoplopomatoidei in previous classifications).

*Morphological synapomorphies*: H Imamura, S Shirai and M Yabe [342].

- Anoplopomatidae

**Infraorder Zoarcales** (= Zoarcoidei in previous classifications) (100%)

*Morphological synapomorphies*: ME Anderson [343], I Imamura and M Yabe [337].

- Anarhichadidae
- Bathymasteridae (not monophyletic in Fig. 2)
- Cryptacanthodidae
- Pholidae
- Stichaeidae (not monophyletic in Fig. 2).
- Zaproridae
- Zoarcidae
- *Not examined*: Eulophiidae [42, 344], Ptilichthyidae, Scytalinidae.

**Infraorder Gasterosteales** (similar to Gasterosteoidei in other classifications, but excluding Indostomidae) (100%)

*Morphological synapomorphies*: R Britz and GD Johnson [252] and EO Wiley and GD Johnson [57] provided synapomorphies for this clade but their diagnosis included Indostomidae, now placed in the series Anabantaria.

- Aulorhynchidae
- Gasterosteidae
- Hypoptychidae

**Infraorder Zaniolepidoales** (= Zaniolepidoidei *sensu* WL Smith and MS Busby [345]).

*Morphological synapomorphies*: WL Smith and MS Busby [345], H Imamura, S Shirai and M Yabe [342]

- Zaniolepididae (formerly a subfamily of Hexagrammidae [62, 345])

**Infraorder Hexagrammales** (100%) (= Hexagrammoidei in previous classifications)

*Morphological synapomorphies*: WL Smith and MS Busby [345].

*Comment*: Hexagrammidae as formerly defined is not monophyletic. We now split it into two families (formerly subfamilies): Hexagrammidae (*sensu stricto*) and Zaniolepididae [345–347]. As in previous cottoid classifications, these families are placed in their own infraorders (note that previous classifications use suborders instead of infraorders).

- Hexagrammidae (*sensu stricto*; following [345])

**Infraorder Cottales** (99%) (= Cottoidei *sensu* WL Smith and MS Busby [345])

*Morphological synapomorphies*: WL Smith and MS Busby [345].

*Comments*: WL Smith and MS Busby [345] changed the membership of Agonidae (now including the former Hemitripteridae), Cottidae (now including the former Abyssocottidae, Comephoridae, and Cottocomephoridae), and Psycholutridae (now including the former Bathylutichthyidae and many marine genera previously placed in Cottidae) to achieve monophyly of these families. Our phylogenetic results support their revised circumscription.

- Agonidae
- Cottidae
- Cyclopteridae
- Liparidae
- Psychrolutidae
- Scorpaenichthyidae
- Trichodontidae
- *Not examined*: Jordaniidae (following [345]) and Rhamphocottidae (includes the former Ereuniidae; see [345]).

**Superclass Sarcopterygii** (58%)

*Morphological synapomorphies*: see R Cloutier and P Ahlberg [348], HP Schultze and R Cloutier [349], M Zhu, X Yu and P Janvier [118].

*Comment*: Phylogenetic studies on sarcopterygians, based on morphological evidence, include both fossil and extant taxa. Some ranks below are thus redundant in content when only extant taxa are considered (e.g., Dipnomorpha, Ceratodontae and Ceratodontiformes).

**Class Coelacanthimorpha** (= Actinistia).

*Morphological synapomorphies*: see R Cloutier and P Ahlberg [348], H Dutel, JG Maisey, DR Schwimmer, P Janvier, M Herbin and G Clément [350] and G Arratia and HP Schultze [351] (extant taxa only).

**Order Coelacanthiformes**

*Morphological synapomorphies*: same as Coelacanthimorpha (extant taxa only).

- Latimeriidae

**Class Dipnotetrapodomorpha** (100%)

*Morphological synapomorphies*: R Cloutier and P Ahlberg [348].

*Comment*: recent genomic evidence supports the sister-group relationship between lungfishes and tetrapods [352, 353].

**Subclass Dipnomorpha**

*Morphological synapomorphies*: see HP Schultze and KSW Campbell [354], WE Bemis [355], R Cloutier and P Ahlberg [348], G Arratia, HP Schultze and J Casciotta [356] (extant taxa only).

**Superorder Ceratodontae** (= Dipnoi)

*Morphological synapomorphies*: same as Dipnomorpha (extant taxa only).

**Order Ceratodontiformes**

*Morphological synapomorphies*: same as Dipnomorpha (extant taxa only).

**Suborder Ceratodontoidei**

*Morphological synapomorphies*: see Cloutier and P Ahlberg [342].

- Neoceratodontidae

**Suborder Lepidosirenoidei** (100%)

*Morphological synapomorphies*: see Cloutier and P Ahlberg [342].

- Lepidosirenidae
- Protopteridae

**Subclass Tetrapodomorpha** (100%)

## Conclusions

This update of the phylogenetic classification of bony fishes is substantially improved, implementing over a hundred changes (Additional file 3B) relative to the first version published in 2013 [8]. The updated classification is based on a global phylogenetic tree assembled from four different phylogenetic studies that collectively resolve the placement for nearly 2000 species representing 410 families (~80% of the total) of fishes. Citations have been included to refer readers to morphological studies that provide evidence for the monophyly of specific groups, where available. A total of 514 families of bony fishes in 72 orders and 79 suborders are classified in the current version. Several families, however, remain unexamined or lack phylogenetic resolution. Comments are also included to support taxonomic decisions and an exhaustive comparison with conflicting taxonomic groups proposed by others is presented. In summary, rather than maintaining the taxonomic *status quo* that that has prevailed in ichthyology for decades, this classification uses an explicit and robust phylogenetic framework based on a large-scale phylogenetic backbone as well as on multiple recent, clade-specific studies that continue to improve our knowledge of the fish Tree of Life.

## Additional files

Additional file 1: R code (including newick file examples) used for grafting crown clades into the backbone tree. (ZIP 110 kb)

Additional file 2: Complete tree in newick format. See details under legend of Fig. 2. (TRE 125 kb)

Additional file 3: (A) Comment-free classification; (B) list of changes. (DOCX 104 kb)

Additional file 4: Table S1. Spreadsheet with the complete classification. (XLSX 130 kb)

Additional file 5: Figure S1. High resolution image of Figure 1. (PDF 1120 kb)

Additional file 6: Figure S2. High resolution image of Figure 2. (PDF 4040 kb)
