# Supplementary material for: Phylogenetic classification of bony fishes
Source: BMC Evol Biol. 2017 Jul 6;17:162. doi: 10.1186/s12862-017-0958-3 (PMC5501477; doi:10.1186/s12862-017-0958-3)
Supplement: Supplementary file 3 — (A) Comment-free classification; (B) list of changes. (DOCX 104 kb) [file 12862_2017_958_MOESM3_ESM.docx]

**Appendix 1**

**A. Comment-free classification.**

Megaclass Osteichthyes (= extant Euteleostomi)

Superclass Actinopterygii

Class Cladistia

**Order Polypteriformes**

[Polypteridae](http://www.fishbase.org/Summary/FamilySummary.php?ID=31)

Class Actinopteri

Subclass Chondrostei

**Order Acipenseriformes**

[Acipenseridae](http://www.fishbase.org/Summary/FamilySummary.php?ID=32)

[Polyodontidae](http://www.fishbase.org/Summary/FamilySummary.php?ID=33)

Subclass Neopterygii

Infraclass Holostei

**Order Amiiformes** (= extant Halecomorphi)

[Amiidae](http://www.fishbase.org/Summary/FamilySummary.php?ID=35)

**Order Lepisosteiformes** (= extant Ginglymodi)

[Lepisosteidae](http://www.fishbase.org/Summary/FamilySummary.php?ID=34)

Infraclass Teleostei

Megacohort Elopocephalai

Cohort Elopomorpha

**Order Elopiformes**

[Elopidae](http://www.fishbase.org/Summary/FamilySummary.php?ID=46)

[Megalopidae](http://www.fishbase.org/Summary/FamilySummary.php?ID=47)

**Order Albuliformes**

[Albulidae](http://www.fishbase.org/Summary/FamilySummary.php?ID=48)

**Order Notacanthiformes**

[Halosauridae](http://www.fishbase.org/Summary/FamilySummary.php?ID=71)

[Notacanthidae](http://www.fishbase.org/Summary/FamilySummary.php?ID=73)

**Order Anguilliformes**

[Anguillidae](http://www.fishbase.org/Summary/FamilySummary.php?ID=49)

[Chlopsidae](http://www.fishbase.org/Summary/FamilySummary.php?ID=54)

[Congridae](http://www.fishbase.org/Summary/FamilySummary.php?ID=62)

[Cyematidae](http://www.fishbase.org/Summary/FamilySummary.php?ID=53)

[Derichthyidae](http://www.fishbase.org/Summary/FamilySummary.php?ID=67)

[Eurypharyngidae](http://www.fishbase.org/Summary/FamilySummary.php?ID=69)

[Heterenchelyidae](http://www.fishbase.org/Summary/FamilySummary.php?ID=50)

[Monognathidae](http://www.fishbase.org/Summary/FamilySummary.php?ID=70)

[Moringuidae](http://www.fishbase.org/Summary/FamilySummary.php?ID=51)

[Muraenesocidae](http://www.fishbase.org/Summary/FamilySummary.php?ID=504)

[Muraenidae](http://www.fishbase.org/Summary/FamilySummary.php?ID=56)

[Myrocongridae](http://www.fishbase.org/Summary/FamilySummary.php?ID=55)

[Nemichthyidae](http://www.fishbase.org/Summary/FamilySummary.php?ID=52)

[Nettastomatidae](http://www.fishbase.org/Summary/FamilySummary.php?ID=64)

[Ophichthidae](http://www.fishbase.org/Summary/FamilySummary.php?ID=66)

[Protanguillidae](http://www.fishbase.org/Summary/FamilySummary.php?ID=677)

[Saccopharyngidae](http://www.fishbase.org/Summary/FamilySummary.php?ID=68)

[Serrivomeridae](http://www.fishbase.org/Summary/FamilySummary.php?ID=65)

[Synaphobranchidae](http://www.fishbase.org/Summary/FamilySummary.php?ID=57)

Megacohort Osteoglossocephalai (= Osteoglossocephala)

Supercohort Osteoglossomorpha

**Order Hiodontiformes**

[Hiodontidae](http://www.fishbase.org/Summary/FamilySummary.php?ID=36)

**Order Osteoglossiformes**

[Gymnarchidae](http://www.fishbase.org/Summary/FamilySummary.php?ID=41)

[Mormyridae](http://www.fishbase.org/Summary/FamilySummary.php?ID=40)

[Notopteridae](http://www.fishbase.org/Summary/FamilySummary.php?ID=37)

[Osteoglossidae](http://www.fishbase.org/Summary/FamilySummary.php?ID=38)

[Pantodontidae](http://www.fishbase.org/Summary/FamilySummary.php?ID=39)

Supercohort Clupeocephala

Cohort Otomorpha

Subcohort Clupei (= Clupeomorpha)

**Order Clupeiformes**

**Suborder Denticipitoidei**

[Denticipitidae](http://www.fishbase.org/Summary/FamilySummary.php?ID=42)

**Suborder Clupeoidei**

[Chirocentridae](http://www.fishbase.org/Summary/FamilySummary.php?ID=45)

[Clupeidae](http://www.fishbase.org/Summary/FamilySummary.php?ID=43)

[Engraulidae](http://www.fishbase.org/Summary/FamilySummary.php?ID=454)

[Pristigasteridae](http://www.fishbase.org/Summary/FamilySummary.php?ID=620)

Subcohort Alepocephali

**Order Alepocephaliformes**

[Alepocephalidae](http://www.fishbase.org/Summary/FamilySummary.php?ID=86)

[Platytroctidae](http://www.fishbase.org/Summary/FamilySummary.php?ID=87)

Subcohort Ostariophysi

Section Anotophysa (= Anotophysi)

**Order Gonorynchiformes**

[Gonorynchidae](http://www.fishbase.org/Summary/FamilySummary.php?ID=101)

[Chanidae](http://www.fishbase.org/Summary/FamilySummary.php?ID=98)

[Kneriidae](http://www.fishbase.org/Summary/FamilySummary.php?ID=99)

Section Otophysa (= Otophysi)

Superorder Cypriniphysae

**Order Cypriniformes**

**Suborder Gyrinocheiloidei**

[Gyrinocheilidae](http://www.fishbase.org/Summary/FamilySummary.php?ID=123)

**Suborder Catostomoidei**

[Catostomidae](http://www.fishbase.org/Summary/FamilySummary.php?ID=125)

**Suborder Cobitoidei**

[Balitoridae](http://www.fishbase.org/Summary/FamilySummary.php?ID=126)

[Barbuccidae](http://www.fishbase.org/Summary/FamilySummary.php?ID=697)

[Botiidae](http://www.fishbase.org/Summary/FamilySummary.php?ID=696)

[Cobitidae](http://www.fishbase.org/Summary/FamilySummary.php?ID=127)

[Ellopostomatidae](http://www.fishbase.org/Summary/FamilySummary.php?ID=702)

Gastromyzontidae

[Nemacheilidae](http://www.fishbase.org/Summary/FamilySummary.php?ID=692)

[Serpenticobitidae](http://www.fishbase.org/Summary/FamilySummary.php?ID=698)

[Vaillantellidae](http://www.fishbase.org/Summary/FamilySummary.php?ID=691)

**Suborder Cyprinoidei**

Acheilognathidae

[Cyprinidae](http://www.fishbase.org/Summary/FamilySummary.php?ID=122)

Danionidae

Gobionidae

Leuciscidae

Leptobarbidae

Paedocyprididae

[Psilorhynchidae](http://www.fishbase.org/Summary/FamilySummary.php?ID=124)

Sundadanionidae

Tanichthyidae

Tincidae

Xenocyprididae

Superorder Characiphysae (= Characiphysi)

**Order Characiformes**

**Suborder Citharinoidei**

[Citharinidae](http://www.fishbase.org/Summary/FamilySummary.php?ID=116)

[Distichodontidae](http://www.fishbase.org/Summary/FamilySummary.php?ID=671)

**Suborder Characoidei**

[Acestrorhynchidae](http://www.fishbase.org/Summary/FamilySummary.php?ID=593)

[Alestidae](http://www.fishbase.org/Summary/FamilySummary.php?ID=518)

[Anostomidae](http://www.fishbase.org/Summary/FamilySummary.php?ID=559)

[Bryconidae](http://www.fishbase.org/Summary/FamilySummary.php?ID=687)

[Chalceidae](http://www.fishbase.org/Summary/FamilySummary.php?ID=690)

[Characidae](http://www.fishbase.org/Summary/FamilySummary.php?ID=102)

[Chilodontidae](http://www.fishbase.org/Summary/FamilySummary.php?ID=602)

[Crenuchidae](http://www.fishbase.org/Summary/FamilySummary.php?ID=655)

[Ctenoluciidae](http://www.fishbase.org/Summary/FamilySummary.php?ID=104)

[Curimatidae](http://www.fishbase.org/Summary/FamilySummary.php?ID=111)

[Cynodontidae](http://www.fishbase.org/Summary/FamilySummary.php?ID=527)

[Erythrinidae](http://www.fishbase.org/Summary/FamilySummary.php?ID=103)

[Gasteropelecidae](http://www.fishbase.org/Summary/FamilySummary.php?ID=109)

[Hemiodontidae](http://www.fishbase.org/Summary/FamilySummary.php?ID=113)

[Hepsetidae](http://www.fishbase.org/Summary/FamilySummary.php?ID=105)

[Iguanodectidae](http://www.fishbase.org/Summary/FamilySummary.php?ID=689)

[Lebiasinidae](http://www.fishbase.org/Summary/FamilySummary.php?ID=107)

[Parodontidae](http://www.fishbase.org/Summary/FamilySummary.php?ID=603)

[Prochilodontidae](http://www.fishbase.org/Summary/FamilySummary.php?ID=598)

[Serrasalmidae](http://www.fishbase.org/Summary/FamilySummary.php?ID=686)

[Triportheidae](http://www.fishbase.org/Summary/FamilySummary.php?ID=688)

Superorder Siluriphysae (= Siluriphysi)

**Order Gymnotiformes**

**Suborder Gymnotoidei**

[Gymnotidae](http://www.fishbase.org/Summary/FamilySummary.php?ID=118)

**Suborder Sternopygoidei**

[Apteronotidae](http://www.fishbase.org/Summary/FamilySummary.php?ID=545)

[Hypopomidae](http://www.fishbase.org/Summary/FamilySummary.php?ID=546)

[Rhamphichthyidae](http://www.fishbase.org/Summary/FamilySummary.php?ID=547)

[Sternopygidae](http://www.fishbase.org/Summary/FamilySummary.php?ID=548)

**Order Siluriformes**

**Suborder Loricarioidei**

[Astroblepidae](http://www.fishbase.org/Summary/FamilySummary.php?ID=158)

[Callichthyidae](http://www.fishbase.org/Summary/FamilySummary.php?ID=156)

[Loricariidae](http://www.fishbase.org/Summary/FamilySummary.php?ID=157)

[Nematogenyidae](http://www.fishbase.org/Summary/FamilySummary.php?ID=613)

[Scoloplacidae](http://www.fishbase.org/Summary/FamilySummary.php?ID=539)

[Trichomycteridae](http://www.fishbase.org/Summary/FamilySummary.php?ID=155)

**Suborder Diplomystoidei**

[Diplomystidae](http://www.fishbase.org/Summary/FamilySummary.php?ID=128)

**Suborder Siluroidei**

Ailiidae

[Akysidae](http://www.fishbase.org/Summary/FamilySummary.php?ID=137)

[Amblycipitidae](http://www.fishbase.org/Summary/FamilySummary.php?ID=135)

[Amphiliidae](http://www.fishbase.org/Summary/FamilySummary.php?ID=136)

[Anchariidae](http://www.fishbase.org/Summary/FamilySummary.php?ID=660)

[Ariidae](http://www.fishbase.org/Summary/FamilySummary.php?ID=145)

[Aspredinidae](http://www.fishbase.org/Summary/FamilySummary.php?ID=148)

[Auchenipteridae](http://www.fishbase.org/Summary/FamilySummary.php?ID=147)

Auchenoglanididae

[Austroglanididae](http://www.fishbase.org/Summary/FamilySummary.php?ID=643)

[Bagridae](http://www.fishbase.org/Summary/FamilySummary.php?ID=130)

[Cetopsidae](http://www.fishbase.org/Summary/FamilySummary.php?ID=154)

[Chacidae](http://www.fishbase.org/Summary/FamilySummary.php?ID=141)

[Clariidae](http://www.fishbase.org/Summary/FamilySummary.php?ID=139)

[Claroteidae](http://www.fishbase.org/Summary/FamilySummary.php?ID=668)

[Cranoglanididae](http://www.fishbase.org/Summary/FamilySummary.php?ID=131)

[Doradidae](http://www.fishbase.org/Summary/FamilySummary.php?ID=146)

[Erethistidae](http://www.fishbase.org/Summary/FamilySummary.php?ID=592)

[Heptapteridae](http://www.fishbase.org/Summary/FamilySummary.php?ID=596)

[Heteropneustidae](http://www.fishbase.org/Summary/FamilySummary.php?ID=140)

[Horabagridae](http://www.fishbase.org/Summary/FamilySummary.php?ID=684)

[Ictaluridae](http://www.fishbase.org/Summary/FamilySummary.php?ID=129)

Kryptoglanidae

[Lacantuniidae](http://www.fishbase.org/Summary/FamilySummary.php?ID=637)

[Malapteruridae](http://www.fishbase.org/Summary/FamilySummary.php?ID=143)

[Mochokidae](http://www.fishbase.org/Summary/FamilySummary.php?ID=144)

[Pangasiidae](http://www.fishbase.org/Summary/FamilySummary.php?ID=134)

[Pimelodidae](http://www.fishbase.org/Summary/FamilySummary.php?ID=150)

[Plotosidae](http://www.fishbase.org/Summary/FamilySummary.php?ID=149)

[Pseudopimelodidae](http://www.fishbase.org/Summary/FamilySummary.php?ID=609)

Ritidae

[Schilbeidae](http://www.fishbase.org/Summary/FamilySummary.php?ID=133)

[Siluridae](http://www.fishbase.org/Summary/FamilySummary.php?ID=132)

[Sisoridae](http://www.fishbase.org/Summary/FamilySummary.php?ID=138)

(Supercohort Clupeocephala; continue)

Cohort Euteleosteomorpha (= Euteleostei)

Subcohort Lepidogalaxii

**Order Lepidogalaxiiformes**

[Lepidogalaxiidae](http://www.fishbase.org/Summary/FamilySummary.php?ID=466)

Subcohort Protacanthopterygii

**Order Argentiniformes**

[Argentinidae](http://www.fishbase.org/Summary/FamilySummary.php?ID=83)

[Bathylagidae](http://www.fishbase.org/Summary/FamilySummary.php?ID=84)

[Microstomatidae](http://www.fishbase.org/Summary/FamilySummary.php?ID=521)

[Opisthoproctidae](http://www.fishbase.org/Summary/FamilySummary.php?ID=85)

**Order Galaxiiformes**

[Galaxiidae](http://www.fishbase.org/Summary/FamilySummary.php?ID=79)

**Order Salmoniformes**

[Salmonidae](http://www.fishbase.org/Summary/FamilySummary.php?ID=76)

**Order Esociformes**

[Esocidae](http://www.fishbase.org/Summary/FamilySummary.php?ID=74)

[Umbridae](http://www.fishbase.org/summary/FamilySummary.php?ID=75)

Subcohort Stomiati

**Order Stomiatiformes**(= Stomiiformes)

**Suborder Gonostomatoidei**

[Gonostomatidae](http://www.fishbase.org/Summary/FamilySummary.php?ID=88)

**Suborder Stomiatoidei** (= Phosichthyoidei)

[Phosichthyidae](http://www.fishbase.org/Summary/FamilySummary.php?ID=468)

[Sternoptychidae](http://www.fishbase.org/Summary/FamilySummary.php?ID=89)

[Stomiidae](http://www.fishbase.org/Summary/FamilySummary.php?ID=91)

**Order Osmeriformes**

**Suborder Osmeroidei**

[Osmeridae](http://www.fishbase.org/Summary/FamilySummary.php?ID=80)

[Plecoglossidae](http://www.fishbase.org/Summary/FamilySummary.php?ID=81)

[Salangidae](http://www.fishbase.org/Summary/FamilySummary.php?ID=82)

**Suborder Retropinnoidei**

[Retropinnidae](http://www.fishbase.org/Summary/FamilySummary.php?ID=77)

Subcohort Neoteleostei

Infracohort Ateleopodia (= Ateleopodomorpha)

**Order Ateleopodiformes**

[Ateleopodidae](http://www.fishbase.org/Summary/FamilySummary.php?ID=228)

Infracohort Eurypterygia (= Eurypterygii)

Section Cyclosquamata (= Aulopa)

**Order Aulopiformes**

**Suborder Aulopoidei** (= Synodontoidei)

[Aulopidae](http://www.fishbase.org/Summary/FamilySummary.php?ID=159)

[Pseudotrichonotidae](http://www.fishbase.org/Summary/FamilySummary.php?ID=471)

[Synodontidae](http://www.fishbase.org/Summary/FamilySummary.php?ID=160)

**Suborder Paraulopoidei**

[Paraulopidae](http://www.fishbase.org/Summary/FamilySummary.php?ID=614)

**Suborder Alepisauroidei**

[Alepisauridae](http://www.fishbase.org/Summary/FamilySummary.php?ID=171)

[Bathysauridae](http://www.fishbase.org/Summary/FamilySummary.php?ID=654)

[Bathysauroididae](http://www.fishbase.org/Summary/FamilySummary.php?ID=626)

[Bathysauropsidae](http://www.fishbase.org/Summary/FamilySummary.php?ID=641)

[Chlorophthalmidae](http://www.fishbase.org/Summary/FamilySummary.php?ID=163)

[Evermannellidae](http://www.fishbase.org/Summary/FamilySummary.php?ID=173)

[Giganturidae](http://www.fishbase.org/Summary/FamilySummary.php?ID=96)

[Ipnopidae](http://www.fishbase.org/Summary/FamilySummary.php?ID=560)

Lestidiidae

[Notosudidae](http://www.fishbase.org/Summary/FamilySummary.php?ID=470)

[Paralepididae](http://www.fishbase.org/Summary/FamilySummary.php?ID=169)

[Scopelarchidae](http://www.fishbase.org/Summary/FamilySummary.php?ID=174)

[Sudidae](http://www.fishbase.org/Summary/FamilySummary.php?ID=665)

Section Ctenosquamata

Subsection Myctophata (= Scopelomorpha)

**Order Myctophiformes**

[Myctophidae](http://www.fishbase.org/Summary/FamilySummary.php?ID=167)

[Neoscopelidae](http://www.fishbase.org/Summary/FamilySummary.php?ID=168)

Subsection Acanthomorphata (= Acanthomorpha)

Division Lampripterygii (= Lampridacea; = Lamprimorpha)

**Order Lampriformes** (= Lampridiformes; = Allotriognathi)

[Lampridae](http://www.fishbase.org/Summary/FamilySummary.php?ID=222)

[Lophotidae](http://www.fishbase.org/Summary/FamilySummary.php?ID=224)

[Radiicephalidae](http://www.fishbase.org/Summary/FamilySummary.php?ID=477)

[Regalecidae](http://www.fishbase.org/Summary/FamilySummary.php?ID=226)

[Trachipteridae](http://www.fishbase.org/Summary/FamilySummary.php?ID=225)

[Veliferidae](http://www.fishbase.org/Summary/FamilySummary.php?ID=223)

Division Paracanthopterygii (= Paracanthomorphacea)

Series Percopsaria

**Order Percopsiformes**

[Amblyopsidae](http://www.fishbase.org/Summary/FamilySummary.php?ID=178)

[Aphredoderidae](http://www.fishbase.org/Summary/FamilySummary.php?ID=177)

[Percopsidae](http://www.fishbase.org/Summary/FamilySummary.php?ID=176)

Series Zeiogadaria (= Zeiogadiformes)

Subseries Zeiariae

**Order Zeiformes**

**Suborder Cyttoidei**

[Cyttidae](http://www.fishbase.org/Summary/FamilySummary.php?ID=629)

**Suborder Zeiodei**

[Parazenidae](http://www.fishbase.org/Summary/FamilySummary.php?ID=247)

[Grammicolepididae](http://www.fishbase.org/Summary/FamilySummary.php?ID=251)

[Oreosomatidae](http://www.fishbase.org/Summary/FamilySummary.php?ID=250)

[Zeidae](http://www.fishbase.org/Summary/FamilySummary.php?ID=249)

[Zeniontidae](http://www.fishbase.org/Summary/FamilySummary.php?ID=248) (= [Zenionidae](http://www.fishbase.org/Summary/FamilySummary.php?ID=248))

Subseries Gadariae

**Order Stylephoriformes**

[Stylephoridae](http://www.fishbase.org/Summary/FamilySummary.php?ID=227)

**Order Gadiformes**

Bathygadidae

[Bregmacerotidae](http://www.fishbase.org/Summary/FamilySummary.php?ID=182)

[Euclichthyidae](http://www.fishbase.org/Summary/FamilySummary.php?ID=537)

[Gadidae](http://www.fishbase.org/Summary/FamilySummary.php?ID=183)

Gaidropsaridae

[Macrouridae](http://www.fishbase.org/Summary/FamilySummary.php?ID=185)

Macruronidae

[Merlucciidae](http://www.fishbase.org/Summary/FamilySummary.php?ID=184)

[Melanonidae](http://www.fishbase.org/Summary/FamilySummary.php?ID=181)

[Moridae](http://www.fishbase.org/Summary/FamilySummary.php?ID=180)

[Muraenolepididae](http://www.fishbase.org/Summary/FamilySummary.php?ID=179)

[Phycidae](http://www.fishbase.org/Summary/FamilySummary.php?ID=506)

Ranicipitidae

[Steindachneriidae](http://www.fishbase.org/Summary/FamilySummary.php?ID=666)

Trachyrincidae

Division Polymixiipterygii

**Order Polymixiiformes**

[Polymixiidae](http://www.fishbase.org/Summary/FamilySummary.php?ID=238)

Division Acanthopterygii (= Euacanthomorphacea)

Subdivision Berycimorphaceae

**Order Beryciformes**

**Suborder Berycoidei**

[Berycidae](http://www.fishbase.org/Summary/FamilySummary.php?ID=240)

[Melamphaidae](http://www.fishbase.org/Summary/FamilySummary.php?ID=233)

**Suborder Stephanoberycoidei**

[Barbourisiidae](http://www.fishbase.org/Summary/FamilySummary.php?ID=675)

[Cetomimidae](http://www.fishbase.org/Summary/FamilySummary.php?ID=246)

[Gibberichthyidae](http://www.fishbase.org/Summary/FamilySummary.php?ID=234)

[Hispidoberycidae](http://www.fishbase.org/Summary/FamilySummary.php?ID=540)

[Rondeletiidae](http://www.fishbase.org/Summary/FamilySummary.php?ID=676)

[Stephanoberycidae](http://www.fishbase.org/Summary/FamilySummary.php?ID=232)

**Order Trachichthyiformes**

[Anomalopidae](http://www.fishbase.org/Summary/FamilySummary.php?ID=242)

[Anoplogastridae](http://www.fishbase.org/Summary/FamilySummary.php?ID=239)

[Diretmidae](http://www.fishbase.org/Summary/FamilySummary.php?ID=237)

[Monocentridae](http://www.fishbase.org/Summary/FamilySummary.php?ID=241)

[Trachichthyidae](http://www.fishbase.org/Summary/FamilySummary.php?ID=236)

Subdivision Holocentrimorphaceae

**Order Holocentriformes**

[Holocentridae](http://www.fishbase.org/Summary/FamilySummary.php?ID=243)

Subdivision Percomorphaceae (= Percomorpha)

(Subdivision Percomorphaceae; continue)

Series Ophidiaria

**Order Ophidiiformes**

**Suborder Ophidioidei**

[Ophidiidae](http://www.fishbase.org/Summary/FamilySummary.php?ID=186)

**Suborder Bythitoidei**

Dinematichthyidae

[Bythitidae](http://www.fishbase.org/Summary/FamilySummary.php?ID=472)

Series Batrachoidaria

**Order Batrachoidiformes**

[Batrachoididae](http://www.fishbase.org/Summary/FamilySummary.php?ID=189)

Series Pelagiaria (= Stromateoidei; = Pelagia)

**Order Scombriformes**

[Amarsipidae](http://www.fishbase.org/Summary/FamilySummary.php?ID=420)

[Ariommatidae](http://www.fishbase.org/Summary/FamilySummary.php?ID=423)

[Arripidae](http://www.fishbase.org/Summary/FamilySummary.php?ID=321)

[Bramidae](http://www.fishbase.org/Summary/FamilySummary.php?ID=319)

[Caristiidae](http://www.fishbase.org/Summary/FamilySummary.php?ID=320)

[Centrolophidae](http://www.fishbase.org/Summary/FamilySummary.php?ID=421)

[Chiasmodontidae](http://www.fishbase.org/Summary/FamilySummary.php?ID=368)

[Gempylidae](http://www.fishbase.org/Summary/FamilySummary.php?ID=414)

[Icosteidae](http://www.fishbase.org/Summary/FamilySummary.php?ID=400)

[Nomeidae](http://www.fishbase.org/Summary/FamilySummary.php?ID=422)

[Pomatomidae](http://www.fishbase.org/Summary/FamilySummary.php?ID=311)

[Scombridae](http://www.fishbase.org/Summary/FamilySummary.php?ID=416)

[Scombrolabracidae](http://www.fishbase.org/Summary/FamilySummary.php?ID=484)

[Scombropidae](http://www.fishbase.org/Summary/FamilySummary.php?ID=529)

[Stromateidae](http://www.fishbase.org/Summary/FamilySummary.php?ID=425)

[Tetragonuridae](http://www.fishbase.org/Summary/FamilySummary.php?ID=424)

[Trichiuridae](http://www.fishbase.org/Summary/FamilySummary.php?ID=415)

Series Syngnatharia

**Order Syngnathiformes**

**Suborder Syngnathoidei**

[Aulostomidae](http://www.fishbase.org/Summary/FamilySummary.php?ID=253)

[Centriscidae](http://www.fishbase.org/Summary/FamilySummary.php?ID=256)

[Fistulariidae](http://www.fishbase.org/Summary/FamilySummary.php?ID=254)

[Solenostomidae](http://www.fishbase.org/Summary/FamilySummary.php?ID=257)

[Syngnathidae](http://www.fishbase.org/Summary/FamilySummary.php?ID=258)

**Suborder Dactylopteroidei**

[Dactylopteridae](http://www.fishbase.org/Summary/FamilySummary.php?ID=285)

[Pegasidae](http://www.fishbase.org/Summary/FamilySummary.php?ID=286)

**Suborder Callionymoidei** (= Callionymiformes)

[Callionymidae](http://www.fishbase.org/Summary/FamilySummary.php?ID=435)

[Draconettidae](http://www.fishbase.org/Summary/FamilySummary.php?ID=436)

**Suborder Mulloidei**

[Mullidae](http://www.fishbase.org/Summary/FamilySummary.php?ID=332)

Series Gobiaria

**Order Kurtiformes**

**Suborder Kurtoidei**

[Kurtidae](http://www.fishbase.org/Summary/FamilySummary.php?ID=411)

**Suborder Apogonoidei**

[Apogonidae](http://www.fishbase.org/Summary/FamilySummary.php?ID=304)

**Order Gobiiformes**

**Suborder Trichonotoidei**

[Trichonotidae](http://www.fishbase.org/Summary/FamilySummary.php?ID=372)

**Suborder Gobioidei**

Butidae

[Eleotridae](http://www.fishbase.org/Summary/FamilySummary.php?ID=404)

[Gobiidae](http://www.fishbase.org/Summary/FamilySummary.php?ID=405)

Milyeringidae

[Odontobutidae](http://www.fishbase.org/Summary/FamilySummary.php?ID=549)

Oxudercidae

[Rhyacichthyidae](http://www.fishbase.org/Summary/FamilySummary.php?ID=406)

[Thalasseleotrididae](http://www.fishbase.org/Summary/FamilySummary.php?ID=681)

Series Anabantaria

**Order Synbranchiformes**

**Suborder Mastacembeloidei**

[Chaudhuriidae](http://www.fishbase.org/Summary/FamilySummary.php?ID=433)

[Mastacembelidae](http://www.fishbase.org/Summary/FamilySummary.php?ID=432)

**Suborder Indostomoidei**

[Indostomidae](http://www.fishbase.org/Summary/FamilySummary.php?ID=205)

**Suborder Synbranchoidei**

[Synbranchidae](http://www.fishbase.org/Summary/FamilySummary.php?ID=262)

**Order Anabantiformes** (= Labyrinthici)

**Suborder Anabantoidei**

[Anabantidae](http://www.fishbase.org/Summary/FamilySummary.php?ID=426)

[Helostomatidae](http://www.fishbase.org/Summary/FamilySummary.php?ID=428)

[Osphronemidae](http://www.fishbase.org/Summary/FamilySummary.php?ID=429)

**Suborder Channoidei**

[Channidae](http://www.fishbase.org/Summary/FamilySummary.php?ID=431)

**Suborder Nandoidei**

[Badidae](http://www.fishbase.org/Summary/FamilySummary.php?ID=619)

[Nandidae](http://www.fishbase.org/Summary/FamilySummary.php?ID=346)

[Pristolepididae](http://www.fishbase.org/Summary/FamilySummary.php?ID=693)

Series Carangaria (= Carangimorpha)

**Order*-*level *incertae sedis* in Carangaria**

[Centropomidae](http://www.fishbase.org/Summary/FamilySummary.php?ID=287)

[Lactariidae](http://www.fishbase.org/Summary/FamilySummary.php?ID=310)

[Leptobramidae](http://www.fishbase.org/Summary/FamilySummary.php?ID=335)

[Menidae](http://www.fishbase.org/Summary/FamilySummary.php?ID=317)

[Polynemidae](http://www.fishbase.org/Summary/FamilySummary.php?ID=361)

[Sphyraenidae](http://www.fishbase.org/Summary/FamilySummary.php?ID=360)

[Toxotidae](http://www.fishbase.org/Summary/FamilySummary.php?ID=337)

**Order Istiophoriformes** (= Xiphiicae)

[Istiophoridae](http://www.fishbase.org/Summary/FamilySummary.php?ID=419)

[Xiphiidae](http://www.fishbase.org/Summary/FamilySummary.php?ID=417)

**Order Carangiformes**

[Carangidae](http://www.fishbase.org/Summary/FamilySummary.php?ID=314)

[Coryphaenidae](http://www.fishbase.org/Summary/FamilySummary.php?ID=315)

[Echeneidae](http://www.fishbase.org/Summary/FamilySummary.php?ID=313)

[Nematistiidae](http://www.fishbase.org/Summary/FamilySummary.php?ID=478)

[Rachycentridae](http://www.fishbase.org/Summary/FamilySummary.php?ID=312)

**Order Pleuronectiformes**

**Suborder Psettodoidei**

[Psettodidae](http://www.fishbase.org/Summary/FamilySummary.php?ID=437)

**Suborder Pleuronectoidei**

[Achiridae](http://www.fishbase.org/Summary/FamilySummary.php?ID=516)

[Bothidae](http://www.fishbase.org/Summary/FamilySummary.php?ID=439)

[Citharidae](http://www.fishbase.org/Summary/FamilySummary.php?ID=438)

[Cynoglossidae](http://www.fishbase.org/Summary/FamilySummary.php?ID=442)

"Cyclopsettidae"

[Paralichthyidae](http://www.fishbase.org/Summary/FamilySummary.php?ID=514)

[Paralichthodidae](http://www.fishbase.org/Summary/FamilySummary.php?ID=678)

[Pleuronectidae](http://www.fishbase.org/Summary/FamilySummary.php?ID=440)

[Poecilopsettidae](http://www.fishbase.org/Summary/FamilySummary.php?ID=670)

[Rhombosoleidae](http://www.fishbase.org/Summary/FamilySummary.php?ID=704)

[Samaridae](http://www.fishbase.org/Summary/FamilySummary.php?ID=589)

[Scophthalmidae](http://www.fishbase.org/Summary/FamilySummary.php?ID=515)

[Soleidae](http://www.fishbase.org/Summary/FamilySummary.php?ID=441)

Series Ovalentaria (= Stiassnyiformes)

**Order-level *incertae sedis* in Ovalentaria**

[Ambassidae](http://www.fishbase.org/Summary/FamilySummary.php?ID=509)

Congrogadidae

[Embiotocidae](http://www.fishbase.org/Summary/FamilySummary.php?ID=348)

[Grammatidae](http://www.fishbase.org/Summary/FamilySummary.php?ID=293)

[Opistognathidae](http://www.fishbase.org/Summary/FamilySummary.php?ID=366)

[Plesiopidae](http://www.fishbase.org/Summary/FamilySummary.php?ID=294)

[Polycentridae](http://www.fishbase.org/Summary/FamilySummary.php?ID=618)

[Pomacentridae](http://www.fishbase.org/Summary/FamilySummary.php?ID=350)

[Pseudochromidae](http://www.fishbase.org/Summary/FamilySummary.php?ID=291)

Superorder Cichlomorphae

**Order Cichliformes**

[Cichlidae](http://www.fishbase.org/Summary/FamilySummary.php?ID=349)

[Pholidichthyidae](http://www.fishbase.org/Summary/FamilySummary.php?ID=481)

Superoder Atherinomorphae (= Atherinomorpha)

**Order Atheriniformes**

**Suborder Atherinoidei**

[Atherinidae](http://www.fishbase.org/Summary/FamilySummary.php?ID=218)

Atherionidae

[Bedotiidae](http://www.fishbase.org/Summary/FamilySummary.php?ID=563)

[Dentatherinidae](http://www.fishbase.org/Summary/FamilySummary.php?ID=568)

[Isonidae](http://www.fishbase.org/Summary/FamilySummary.php?ID=644)

[Melanotaeniidae](http://www.fishbase.org/Summary/FamilySummary.php?ID=564)

[Phallostethidae](http://www.fishbase.org/Summary/FamilySummary.php?ID=221)

[Pseudomugilidae](http://www.fishbase.org/Summary/FamilySummary.php?ID=565)

[Telmatherinidae](http://www.fishbase.org/Summary/FamilySummary.php?ID=567)

**Suborder Atherinopsoidei**

[Atherinopsidae](http://www.fishbase.org/Summary/FamilySummary.php?ID=623)

**Order Beloniformes**

**Suborder Adrianichthyoidei**

[Adrianichthyidae](http://www.fishbase.org/Summary/FamilySummary.php?ID=210)

**Suborder Belonoidei** (= Exocoetoidei)

[Belonidae](http://www.fishbase.org/Summary/FamilySummary.php?ID=207)

[Exocoetidae](http://www.fishbase.org/Summary/FamilySummary.php?ID=206)

[Hemiramphidae](http://www.fishbase.org/Summary/FamilySummary.php?ID=475)

[Zenarchopteridae](http://www.fishbase.org/Summary/FamilySummary.php?ID=694)

**Order Cyprinodontiformes**

**Suborder Aplocheiloidei**

[Aplocheilidae](http://www.fishbase.org/Summary/FamilySummary.php?ID=476)

[Nothobranchiidae](http://www.fishbase.org/Summary/FamilySummary.php?ID=642)

["Rivulidae"](http://www.fishbase.org/Summary/FamilySummary.php?ID=601)

**Suborder Cyprinodontoidei**

[Cyprinodontidae](http://www.fishbase.org/Summary/FamilySummary.php?ID=212)

[Fundulidae](http://www.fishbase.org/Summary/FamilySummary.php?ID=570)

[Poeciliidae](http://www.fishbase.org/Summary/FamilySummary.php?ID=216)

[Anablepidae](http://www.fishbase.org/Summary/FamilySummary.php?ID=214)

[Goodeidae](http://www.fishbase.org/Summary/FamilySummary.php?ID=213)

“Pantanodontidae”

[Profundulidae](http://www.fishbase.org/Summary/FamilySummary.php?ID=569)

[Valenciidae](http://www.fishbase.org/Summary/FamilySummary.php?ID=571)

Superorder Mugilomorphae

**Order Mugiliformes**

[Mugilidae](http://www.fishbase.org/Summary/FamilySummary.php?ID=359)

Superorder Blenniimorphae

**Order Gobiesociformes** (= Gobiesocoidei)

[Gobiesocidae](http://www.fishbase.org/Summary/FamilySummary.php?ID=434)

**Order Blenniiformes** (= Blennioidei)

[Blenniidae](http://www.fishbase.org/Summary/FamilySummary.php?ID=392)

[Chaenopsidae](http://www.fishbase.org/Summary/FamilySummary.php?ID=483)

[Clinidae](http://www.fishbase.org/Summary/FamilySummary.php?ID=391)

[Dactyloscopidae](http://www.fishbase.org/Summary/FamilySummary.php?ID=380)

[Labrisomidae](http://www.fishbase.org/Summary/FamilySummary.php?ID=482)

[Tripterygiidae](http://www.fishbase.org/Summary/FamilySummary.php?ID=390)

Series Eupercaria (= Percomorpharia)

**Order-level *incertae sedis* in Eupercaria**

[Callanthiidae](http://www.fishbase.org/Summary/FamilySummary.php?ID=511)

[Centrogenyidae](http://www.fishbase.org/Summary/FamilySummary.php?ID=530)

[Dinolestidae](http://www.fishbase.org/Summary/FamilySummary.php?ID=296)

[Dinopercidae](http://www.fishbase.org/Summary/FamilySummary.php?ID=522)

[Emmelichthyidae](http://www.fishbase.org/Summary/FamilySummary.php?ID=322)

[Malacanthidae](http://www.fishbase.org/Summary/FamilySummary.php?ID=308)

[Monodactylidae](http://www.fishbase.org/Summary/FamilySummary.php?ID=333)

[Moronidae](http://www.fishbase.org/Summary/FamilySummary.php?ID=487)

[Parascorpididae](http://www.fishbase.org/Summary/FamilySummary.php?ID=534)

[Pomacanthidae](http://www.fishbase.org/Summary/FamilySummary.php?ID=460)

[Scatophagidae](http://www.fishbase.org/Summary/FamilySummary.php?ID=341)

[Sciaenidae](http://www.fishbase.org/Summary/FamilySummary.php?ID=331)

[Siganidae](http://www.fishbase.org/Summary/FamilySummary.php?ID=413)

[Sillaginidae](http://www.fishbase.org/Summary/FamilySummary.php?ID=307)

**Order Gerreiformes**

[Gerreidae](http://www.fishbase.org/Summary/FamilySummary.php?ID=326)

**Order Uranoscopiformes** (= Paratrachinoidei)

[Ammodytidae](http://www.fishbase.org/Summary/FamilySummary.php?ID=402)

[Cheimarrichthyidae](http://www.fishbase.org/Summary/FamilySummary.php?ID=630)

[Pinguipedidae](http://www.fishbase.org/Summary/FamilySummary.php?ID=371)

[Uranoscopidae](http://www.fishbase.org/Summary/FamilySummary.php?ID=378)

**Order Labriformes**

[Labridae](http://www.fishbase.org/Summary/FamilySummary.php?ID=362)

**Order Ephippiformes**

[Drepaneidae](http://www.fishbase.org/Summary/FamilySummary.php?ID=510)

[Ephippidae](http://www.fishbase.org/Summary/FamilySummary.php?ID=340)

**Order Chaetodontiformes**

[Chaetodontidae](http://www.fishbase.org/Summary/FamilySummary.php?ID=343)

[Leiognathidae](http://www.fishbase.org/Summary/FamilySummary.php?ID=318)

**Order Acanthuriformes**

[Acanthuridae](http://www.fishbase.org/Summary/FamilySummary.php?ID=412)

[Luvaridae](http://www.fishbase.org/Summary/FamilySummary.php?ID=418)

[Zanclidae](http://www.fishbase.org/Summary/FamilySummary.php?ID=520)

**Order Lutjaniformes**

[Haemulidae](http://www.fishbase.org/Summary/FamilySummary.php?ID=327)

[Lutjanidae](http://www.fishbase.org/Summary/FamilySummary.php?ID=323)

**Order Lobotiformes**

[Hapalogenyidae](http://www.fishbase.org/Summary/FamilySummary.php?ID=656)

[Datnioididae](http://www.fishbase.org/Summary/FamilySummary.php?ID=586)

[Lobotidae](http://www.fishbase.org/Summary/FamilySummary.php?ID=325)

**Order Spariformes**

[Lethrinidae](http://www.fishbase.org/Summary/FamilySummary.php?ID=328)

[Nemipteridae](http://www.fishbase.org/Summary/FamilySummary.php?ID=324)

[Sparidae](http://www.fishbase.org/Summary/FamilySummary.php?ID=330)

**Order Priacanthiformes**

[Priacanthidae](http://www.fishbase.org/Summary/FamilySummary.php?ID=303)

[Cepolidae](http://www.fishbase.org/Summary/FamilySummary.php?ID=358)

**Order Caproiformes**

[Caproidae](http://www.fishbase.org/Summary/FamilySummary.php?ID=252)

**Order Lophiiformes**

**Suborder Lophioidei**

[Lophiidae](http://www.fishbase.org/Summary/FamilySummary.php?ID=190)

**Suborder Antennarioidei**

[Antennariidae](http://www.fishbase.org/Summary/FamilySummary.php?ID=192)

[Brachionichthyidae](http://www.fishbase.org/Summary/FamilySummary.php?ID=191)

[Lophichthyidae](http://www.fishbase.org/Summary/FamilySummary.php?ID=541)

[Tetrabrachiidae](http://www.fishbase.org/Summary/FamilySummary.php?ID=535)

**Suborder Chaunacoidei**

[Chaunacidae](http://www.fishbase.org/Summary/FamilySummary.php?ID=193)

**Suborder Ogcocephaloidei**

[Ogcocephalidae](http://www.fishbase.org/Summary/FamilySummary.php?ID=194)

**Suborder Ceratioidei**

[Caulophrynidae](http://www.fishbase.org/Summary/FamilySummary.php?ID=195)

[Centrophrynidae](http://www.fishbase.org/Summary/FamilySummary.php?ID=202)

[Ceratiidae](http://www.fishbase.org/Summary/FamilySummary.php?ID=203)

[Diceratiidae](http://www.fishbase.org/Summary/FamilySummary.php?ID=197)

[Gigantactinidae](http://www.fishbase.org/Summary/FamilySummary.php?ID=200)

[Himantolophidae](http://www.fishbase.org/Summary/FamilySummary.php?ID=198)

[Linophrynidae](http://www.fishbase.org/Summary/FamilySummary.php?ID=204)

[Melanocetidae](http://www.fishbase.org/Summary/FamilySummary.php?ID=196)

[Neoceratiidae](http://www.fishbase.org/Summary/FamilySummary.php?ID=201)

[Oneirodidae](http://www.fishbase.org/Summary/FamilySummary.php?ID=199)

[Thaumatichthyidae](http://www.fishbase.org/Summary/FamilySummary.php?ID=474)

**Order Tetraodontiformes**

**Suborder Triodontoidei**

[Triodontidae](http://www.fishbase.org/Summary/FamilySummary.php?ID=447)

**Suborder Triacanthoidei**

[Triacanthidae](http://www.fishbase.org/Summary/FamilySummary.php?ID=444)

**Suborder Triacanthodoidei**

[Triacanthodidae](http://www.fishbase.org/Summary/FamilySummary.php?ID=443)

**Suborder Tetraodontoidei**

[Diodontidae](http://www.fishbase.org/Summary/FamilySummary.php?ID=449)

[Tetraodontidae](http://www.fishbase.org/Summary/FamilySummary.php?ID=448)

**Suborder Moloidei**

[Molidae](http://www.fishbase.org/Summary/FamilySummary.php?ID=450)

**Suborder Balistoidei**

[Balistidae](http://www.fishbase.org/Summary/FamilySummary.php?ID=445)

[Monacanthidae](http://www.fishbase.org/Summary/FamilySummary.php?ID=517)

**Suborder Ostracioidei**

[Aracanidae](http://www.fishbase.org/Summary/FamilySummary.php?ID=633)

[Ostraciidae](http://www.fishbase.org/Summary/FamilySummary.php?ID=446)

**Order Pempheriformes**

[Acropomatidae](http://www.fishbase.org/Summary/FamilySummary.php?ID=512)

[Banjosidae](http://www.fishbase.org/Summary/FamilySummary.php?ID=300)

[Bathyclupeidae](http://www.fishbase.org/Summary/FamilySummary.php?ID=336)

[Champsodontidae](http://www.fishbase.org/Summary/FamilySummary.php?ID=367)

[Creediidae](http://www.fishbase.org/Summary/FamilySummary.php?ID=375)

[Epigonidae](http://www.fishbase.org/Summary/FamilySummary.php?ID=507)

[Glaucosomatidae](http://www.fishbase.org/Summary/FamilySummary.php?ID=298)

Hemerocoetidae

[Howellidae](http://www.fishbase.org/Summary/FamilySummary.php?ID=657)

[Lateolabracidae](http://www.fishbase.org/Summary/FamilySummary.php?ID=627)

[Leptoscopidae](http://www.fishbase.org/Summary/FamilySummary.php?ID=379)

[Ostracoberycidae](http://www.fishbase.org/Summary/FamilySummary.php?ID=543)

[Pempheridae](http://www.fishbase.org/Summary/FamilySummary.php?ID=334)

[Pentacerotidae](http://www.fishbase.org/Summary/FamilySummary.php?ID=345)

"Percophidae"

[Polyprionidae](http://www.fishbase.org/Summary/FamilySummary.php?ID=513)

[Symphysanodontidae](http://www.fishbase.org/Summary/FamilySummary.php?ID=526)

**Order Centrarchiformes**

**Suborder Centrarchoidei**

[Centrarchidae](http://www.fishbase.org/Summary/FamilySummary.php?ID=302)

[Elassomatidae](http://www.fishbase.org/Summary/FamilySummary.php?ID=508)

[Enoplosidae](http://www.fishbase.org/Summary/FamilySummary.php?ID=344)

[Sinipercidae](http://www.fishbase.org/Summary/FamilySummary.php?ID=667)

**Suborder Cirrhitoidei**

[Aplodactylidae](http://www.fishbase.org/Summary/FamilySummary.php?ID=354)

[Cheilodactylidae](http://www.fishbase.org/Summary/FamilySummary.php?ID=355)

[Chironemidae](http://www.fishbase.org/Summary/FamilySummary.php?ID=353)

[Cirrhitidae](http://www.fishbase.org/Summary/FamilySummary.php?ID=352)

[Latridae](http://www.fishbase.org/Summary/FamilySummary.php?ID=356)

**Suborder Percichthyoidei**

[Percichthyidae](http://www.fishbase.org/Summary/FamilySummary.php?ID=288)

**Suborder Percalatoidei**

"Percalatidae"

**Suborder Terapontoidei**

[Dichistiidae](http://www.fishbase.org/Summary/FamilySummary.php?ID=338)

[Girellidae](http://www.fishbase.org/Summary/FamilySummary.php?ID=700)

[Kuhliidae](http://www.fishbase.org/Summary/FamilySummary.php?ID=301)

[Kyphosidae](http://www.fishbase.org/Summary/FamilySummary.php?ID=339)

Microcanthidae

[Oplegnathidae](http://www.fishbase.org/Summary/FamilySummary.php?ID=347)

Scorpididae

[Terapontidae](http://www.fishbase.org/Summary/FamilySummary.php?ID=299)

**Order Perciformes** (= Serraniformes)

**Suborder Bembropoidei**

[Bembropidae](http://www.fishbase.org/Summary/FamilySummary.php?ID=695)

**Suborder Normanichthyoidei**

[Normanichthyidae](http://www.fishbase.org/Summary/FamilySummary.php?ID=280)

**Suborder Serranoidei**

[Serranidae](http://www.fishbase.org/Summary/FamilySummary.php?ID=289)

**Suborder Percoidei**

[Niphonidae](http://www.fishbase.org/Summary/FamilySummary.php?ID=682)

[Percidae](http://www.fishbase.org/Summary/FamilySummary.php?ID=306)

[Trachinidae](http://www.fishbase.org/Summary/FamilySummary.php?ID=377)

**Suborder Notothenioidei** (= Nototheniiformes)

[Artedidraconidae](http://www.fishbase.org/Summary/FamilySummary.php?ID=591)

[Bathydraconidae](http://www.fishbase.org/Summary/FamilySummary.php?ID=383)

[Bovichtidae](http://www.fishbase.org/Summary/FamilySummary.php?ID=381)

[Channichthyidae](http://www.fishbase.org/Summary/FamilySummary.php?ID=384)

[Eleginopsidae](http://www.fishbase.org/Summary/FamilySummary.php?ID=595)

[Harpagiferidae](http://www.fishbase.org/Summary/FamilySummary.php?ID=480)

[Nototheniidae](http://www.fishbase.org/Summary/FamilySummary.php?ID=382)

[Percophidae](http://www.fishbase.org/Summary/FamilySummary.php?ID=370)

[Pseudaphritidae](http://www.fishbase.org/Summary/FamilySummary.php?ID=594)

**Suborder Scorpaenoidei**

[Apistidae](http://www.fishbase.org/Summary/FamilySummary.php?ID=576)

[Aploactinidae](http://www.fishbase.org/Summary/FamilySummary.php?ID=268)

[Congiopodidae](http://www.fishbase.org/Summary/FamilySummary.php?ID=275)

[Eschmeyeridae](http://www.fishbase.org/Summary/FamilySummary.php?ID=625)

[Gnathanacanthidae](http://www.fishbase.org/Summary/FamilySummary.php?ID=531)

[Neosebastidae](http://www.fishbase.org/Summary/FamilySummary.php?ID=575)

[Pataecidae](http://www.fishbase.org/Summary/FamilySummary.php?ID=269)

[Perryenidae](http://www.fishbase.org/Summary/FamilySummary.php?ID=701)

[Scorpaenidae](http://www.fishbase.org/Summary/FamilySummary.php?ID=264)

[Sebastidae](http://www.fishbase.org/Summary/FamilySummary.php?ID=573)

[Setarchidae](http://www.fishbase.org/Summary/FamilySummary.php?ID=574)

[Synanceiidae](http://www.fishbase.org/Summary/FamilySummary.php?ID=578)

[Tetrarogidae](http://www.fishbase.org/Summary/FamilySummary.php?ID=577)

[Zanclorhynchidae](http://www.fishbase.org/Summary/FamilySummary.php?ID=636)

**Suborder Platycephaloidei** (= Bembroidei)

[Bembridae](http://www.fishbase.org/Summary/FamilySummary.php?ID=528)

[Hoplichthyidae](http://www.fishbase.org/Summary/FamilySummary.php?ID=274)

[Parabembridae](http://www.fishbase.org/Summary/FamilySummary.php?ID=590)

[Platycephalidae](http://www.fishbase.org/Summary/FamilySummary.php?ID=273)

[Plectrogeniidae](http://www.fishbase.org/Summary/FamilySummary.php?ID=579)

**Suborder Triglioidei**

[Peristediidae](http://www.fishbase.org/Summary/FamilySummary.php?ID=581)

[Triglidae](http://www.fishbase.org/Summary/FamilySummary.php?ID=266)

**Suborder Cottoidei** (= Cottimorpha)

Infraorder Anoplopomatales (= Anoplopomatoidei)

[Anoplopomatidae](http://www.fishbase.org/Summary/FamilySummary.php?ID=270)

Infraorder Zoarcales (= Zoarcoidei)

[Anarhichadidae](http://www.fishbase.org/Summary/FamilySummary.php?ID=396)

[Bathymasteridae](http://www.fishbase.org/Summary/FamilySummary.php?ID=369)

[Cryptacanthodidae](http://www.fishbase.org/Summary/FamilySummary.php?ID=394)

[Pholidae](http://www.fishbase.org/Summary/FamilySummary.php?ID=395)

[Stichaeidae](http://www.fishbase.org/Summary/FamilySummary.php?ID=393)

[Zaproridae](http://www.fishbase.org/Summary/FamilySummary.php?ID=398)

[Zoarcidae](http://www.fishbase.org/Summary/FamilySummary.php?ID=188)

Eulophiidae

[Ptilichthyidae](http://www.fishbase.org/Summary/FamilySummary.php?ID=397)

[Scytalinidae](http://www.fishbase.org/Summary/FamilySummary.php?ID=399)

Infraorder Gasterosteales

[Aulorhynchidae](http://www.fishbase.org/Summary/FamilySummary.php?ID=572)

[Gasterosteidae](http://www.fishbase.org/Summary/FamilySummary.php?ID=260)

[Hypoptychidae](http://www.fishbase.org/Summary/FamilySummary.php?ID=403)

Infraorder Zaniolepidoales (= Zaniolepidoidei)

Zaniolepididae

Infraorder Hexagrammales (= Hexagrammoidei)

[Hexagrammidae](http://www.fishbase.org/Summary/FamilySummary.php?ID=271)

Infraorder Cottales

[Agonidae](http://www.fishbase.org/Summary/FamilySummary.php?ID=283)

[Cottidae](http://www.fishbase.org/Summary/FamilySummary.php?ID=277)

[Cyclopteridae](http://www.fishbase.org/Summary/FamilySummary.php?ID=284)

Jordaniidae

[Liparidae](http://www.fishbase.org/Summary/FamilySummary.php?ID=615)

[Psychrolutidae](http://www.fishbase.org/Summary/FamilySummary.php?ID=282)

[Rhamphocottidae](http://www.fishbase.org/Summary/FamilySummary.php?ID=583)

Scorpaenichthyidae

[Trichodontidae](http://www.fishbase.org/Summary/FamilySummary.php?ID=365)

Superclass Sarcopterygi

Class Coelacanthimorpha (= Actinistia)

**Order Coelacanthiformes**

[Latimeriidae](http://www.fishbase.org/Summary/FamilySummary.php?ID=30)

Class Dipnotetrapodomorpha

Subclass Dipnomorpha

Superorder Ceratodontae (= Dipnoi)

**Order Ceratodontiformes**

**Suborder Ceratodontoidei**

[Neoceratodontidae](http://www.fishbase.org/Summary/FamilySummary.php?ID=27)

**Suborder Lepidosirenoidei**

[Lepidosirenidae](http://www.fishbase.org/Summary/FamilySummary.php?ID=28)

[Protopteridae](http://www.fishbase.org/Summary/FamilySummary.php?ID=552)

Subclass Tetrapodomorpha

**B. List of changes**

**List of all changes from**[**version 3**](https://sites.google.com/site/guilleorti/home/classification#Lundberg2007)**(31 July 2014) to version 4 (current). Asterisk (*) denotes changes that were made in accordance with [39].**

Supercohort Elopocephala is no longer classified.

Derichthyidae now includes the former Colocongridae*.

Osteoglossocephala is now named Osteoglossomorpha*.

Clupeidae now includes the round herrings (subfamily Dussumieriinae)*.

Alepocephalidae now includes the former Bathylaconidae and Leptochilichthyidae.

Suborders in Gonorynchiformes are no longer recognized*.

Three otophysan superorders (Cypriniphysae, Characiphysae and Siluriphysae) are now recognized*.

Suborders in Cypriniformes are now recognized.

The Cypriniformes now includes 24 families (vs. 12 in previous versions).

“Loricaroidei” has been corrected to “Loricarioidei” (previously misspelled)*.

The siluriform families Ailidae, Auchenoglanididae, and Ritidae are now recognized*.

Kryptoglanidae is now recognized.

Suborders in Osmeriformes are now classified*.

Retropinnidae now includes the former Prototroctidae.

Suborders in Stomiatiformes are now classified*.

Gonostomatidae now includes the former Diplophidae*.

Aulopa is now named Cyclosquamata*.

The family Lestidiidae is now recognized*.

Endings for the rank “division” have been changed to "-pterygii."

Lampridiformes and Lamprididae are now named Lampriformes and Lampridae, respectively*.

Zeariae has been corrected to Zeiariae (previously misspelled).

Suborders in Zeiformes are now classified*.

Suborders in Gadiformes are no longer classified*.

The family Lotidae is no longer recognized*.

Zenionidae is now named Zeniontidae*.

Euacanthomorphacea is now named Acanthopterygii*.

Beryciformes *sensu* lato is now split into Beryciformes *sensu stricto* (including suborders Berycoidei and Stephanoberycoidei) and Trachichthyifomes*.

Ophidiidae now includes the former Carapidae.

Dinematichthyidae is now recognized.

Aphyonidae and Parabrotulidae are no longer validated.

Suborders in Syngnathiformes have been reclassified based on a recent and robust phylogenomic analysis of the clade.

Classification of suborders in Gobiiformes has been modified: Trichonotus is placed in its own suborder (Trichonotoidei); Odontobutoidei and Eleotroidei are now synonyms of Gobioidei.

Gobiidae now includes the former Microdesmidae, Kraemeriidae, Ptereleotridae, and Schindleriidae.

Butidae, Oxudercidae (= Gobionellidae) and Milyeringidae are now validated.

Gobionellidae is now a junior synonym of Oxudercidae*.

Cichliformes is expanded herein to include Pholidichthyidae (formerly Pholidichthyiformes)*.

Rivulidae is now listed as “Rivulidae.”

“Pantanodontidae” is now listed as potential new family

Suborders in Atheriniformes are now classified.

Gobiesocidae is now placed in Gobiesociformes (formerly Blenniiformes)*.

Gerreiformes is now classified.

Labridae now includes the former Odacidae.

Centrogenyidae is listed again as order-level incertae sedis in Eupercaria (removed from Uranoscopiformes due to unstable placement).

Hapalogenyidae is now listed under Lobotiformes.

Sparidae now includes the former Centracanthidae*.

Priacanthiformes is now classified.

Lujaniformes is now classified.

Caproiformes is now classified*.

Bathyclupeidae, Champsodontidae and Symphysanodontidae are now listed under Pempheriformes.

Pempheriformes “percophids” are now listed as “Percophidae.”

Hemerocoetidae is now validated.

The families Microcanthidae and Scorpididae are now validated (listed subfamilies of Kyphosidae in other classifications).

“Cirrhitioidei” is now corrected to “Cirrhitoidei” (previously misspelled).

Dichistiidae is now listed under Terapontoidei.

Nine families now included in Scorpaenoidei were listed under Perciformes in previous versions of this classification*.

Bembroidei is now named Platycephaloidei and its circumscription is expanded*.

Bembropoidei is newly classified.

Eulophiidae is now recognized*.

Rhamphocottidae now includes the former Ereuniidae*.

Family membership for genera in Agonidae, Cottidae and Psychrolutidae is now modified.

**List of all changes from** [**version 2**](https://sites.google.com/site/guilleorti/home/classification-v-2) **(27 November 2013) to version 3 (31 July 2014)**

Name changes for Series in Percomorphaceae: new names are Ophidiaria, Batrachoidaria, Gobiaria, Syngnatharia, Pelagiaria, Anabantaria, Carangaria, Ovalentaria, and Eupercaria.

Definition of Centrarchiformes now includes the families Centrarchidae, Elassomatidae, Sinipercidae Jordan, Percichthyidae, Enoplosidae, Cirrithidae, Cheilodactylidae, Girellidae, Kuhliidae, Kyphosidae, Oplegnathidae, and Terapontidae. This change sinks the orders Terapontiformes and Cirrhitiformes (recognized in previous versions), since they are now recognized as suborders of Centrarichiformes together with Centrarchoidei and Percichthyoidei. The new ordinal circumscription is relatively well supported (bootstrap value of 77%). Since Percilia is deeply nested within Percichthyidae in our analyses as well as in other studies mentioned above, the family Percilidae is no longer recognized as valid.

Suborders of Anabantiformes now reflect well-supported monophyletic groups, correcting an error in Version 2 and defining a third suborder (Channoidei) for the family Channidae. Affinities of Channidae with other anabantiform families varies among studies. The new scheme with three subroders is robust to this ambiguity.

A new eupercarian order, Chaetodontiformes, is now recognized for the families Chaetodontidae and Leiognathidae. Although support for this clade is weak (50%) in our global analysis, it has been consistently obtained by previous studies with higher nodal support.

Family Arapaimidae no longer recognized (now included in Osteoglossidae).

Suborders in Beloniformes: Belonoidei replaces Exocoetoidei.

Notobranchidae, Rivulidae, and Aplocheilidae are removed from “not examined” in Cyprinodontoidei and listed as “not examined” in Aplocheiloidei.

Family Sundasalangidae no longer recognized because Sundasalanx is nested within Clupeidae.

Family Olyridae no longer recognized. *Olyra* is included in Bagridae.

Scoloplacidae is removed from “not examined” in Siluroidei and listed as “not examined” in Loricaroidei.

*Stathmonotus* is now listed within Labrisomidae. This change renders Chaenopsidae monophyletic.

*Microdesmus* is now listed within Gobiidae. Thus, Microdesmidae is no longer recognized.

Family Caesionidae (previously listed as *insertae sedis* in Eupercaria) no longer recognized as valid. It is now listed as synonym of Lutjanidae.

Family Scaridae is now included in Labridae, rendering Labridae monophyletic.

Family Achiropsettidae no longer recognized. It is considered a synonym of Rhombosoleidae.

Congrogadinae is now recognized as family Congrogadidae. As a consequence, Pseudochromidae is now monophyletic.

Centrogenyidae is removed from order-level *incertae sedis* in Eupercaria and listed as “possibly included” in Uranoscopiformes.

Gaidropsarinae is raised to family, so Lotidae is no longer paraphyletic.

Hexagrammidae is split it into two families (defined as subfamilies by previous studies): Hexagrammidae (*sensu stricto*) and Zaniolepididae. Both families are now placed in their own infraorders (Zaniolepidoales and Hexagrammales), following previous cottoid classifications. Note that previous classifications use suborder instead of infraorders, as presented here.

We now recognize the cottoid families Jordaniidae (not examined) and Scorpaenichthyidae.

**List of all changes from** [**version 1**](https://sites.google.com/site/guilleorti/home/classification-v-1) **(18 April 2013) to version 2 (27 November 2013)**

**Based on new taxa examined:**

Niphonidae: newly validated family; placed in the suborder Percoidei in Perciformes.

Phractolaemidae: now examined but no longer recognized as a family; listed as subfamily of Kneriidae.

Pegasidae: removed from “not examined” in Callionymoidei to examined in Syngnathoidei.

Zenionidae: now examined; same placement.

Carapidae: now examined; same placement.

Muraenolepididae: now examined; same placement.

Callanthiidae: now examined; listed as incertae sedis in Percomorpharia.

Cepolidae: now examined; listed as incertae sedis in Percomorpharia.

Badidae: now examined; same placement.

Hoplichthyidae: now examined; listed as incertae sedis in Perciformes.

Pentacerotidae: now examined; listed in the newly circumscribed Pempheriformes.

Banjosidae: now examined; listed in the newly circumscribed Pempheriformes.

Ostracoberycidae: now examined; listed in the newly circumscribed Pempheriformes.

Stephanoberycidae: now examined; same placement.

Champsodontidae: now examined; listed as *incertae sedis* in Percomorpharia.

Aphyonidae: now examined; same placement.

Bembridae: now examined in Perciformes; placed in the newly classified suborder Bembroidei.

Parabembridae: now examined in Perciformes; placed in the newly classified suborder Bembroidei.

Cheimarrichthyidae: now examined; same placement.

Perciliidae: now examined; listed as incertae sedis in Percomorpharia (note that validation of this family results in the paraphyly of Percichthyidae).

Pristolepididae: now examined, same placement.

Trichodontidae: now examined in Perciformes, infraorder Cottales.

Triodontidae: now examined; placed in the newly classified suborder Triodontoidei.

Anomalopidae: now examined; same placement.

Datnioididae: now examined, placed in the newly classified order Lobotiformes (with Lobotidae).

Phallostethidae: now examined; same placement.

**Other Changes:**

Creediidae is removed from Syngnathiformes and placed in Pempheriformes (Percomorpharia). This is the major topological change in the new tree. The previous version had only four gene sequences for *Limnichthys* (two of which were likely contaminated) and this species is now represented in eight markers.

The order Pempheriformes has a broader circumscription, including the families Acropomatidae, Banjosidae, Creediidae, Epigonidae, Glaucosomatidae, Howellidae, Lateolabracidae, Leptoscopidae, Ostracoberycidae, Pempheridae, Pentacerotidae, Percophidae, and Polyprionidae.

*Bembrops* is now placed in Bembropidae. Percophidae, as traditionally recognized (including Bembropinae), is polyphyletic in the new tree, with *Acanthaphristis* and *Bembrops* falling in different places.

*Girella* is now recognized in its own family, Girellidae. Inclusion of *Girella* in Kyphosidae renders this family non-monophyletic.

The families Girellidae, Kuhliidae, Kyphosidae, Oplegnathidae, and Terapontidae are classified in the new order Terapontiformes in Percomorpharia.

Solenostomidae is removed from “not examined” in Callionymoidei and listed as “not examined” in Syngnathoidei.

Fistulariidae is removed from “possibly included” in Syngnathoidei and listed as *incertae sedis* in Syngnathiformes.

Leptoscopidae is removed from “not examined” in Syngnathiformes (Syngnathimorpharia) and listed in the newly circumscribed Pempheriformes (Percomorpharia).

Pelagimorpharia was adopted to replace Scombrimorpharia. Pelagia was not adopted because it is already in use for a genus of scyphozoan jellyfish (Fam Pelagiidae).

Lactariidae is removed from “not examined” in Percomorphaceae and listed as “not examined” in Carangimorphariae.

Six families traditionally placed in “Perciformes”, and previously listed as incertae sedis in Percomorphaceae, are provisionally listed in Percomorpharia: Bathyclupeidae, Dichistiidae, Hapalogenyidae, Parascorpididae, Symphysanodontidae, Trichonotidae.

The superorder “Cyprinae” was changed to “Cypriniphysae”.

The monophyly of both Ceratioidei and Ceratiidae was challenged in version 1 due to misplacement of *Cryptosaras*. This is no longer the case in the phylogeny supporting version 2, where *Cryptosaras* and *Ceratias* are resolved as sister taxa with 98% bootstrap support.
