# Supplementary figures and images for: Phylogenetic classification of bony fishes
Source: BMC Evol Biol. 2017 Jul 6;17:162. doi: 10.1186/s12862-017-0958-3 (PMC5501477; doi:10.1186/s12862-017-0958-3)

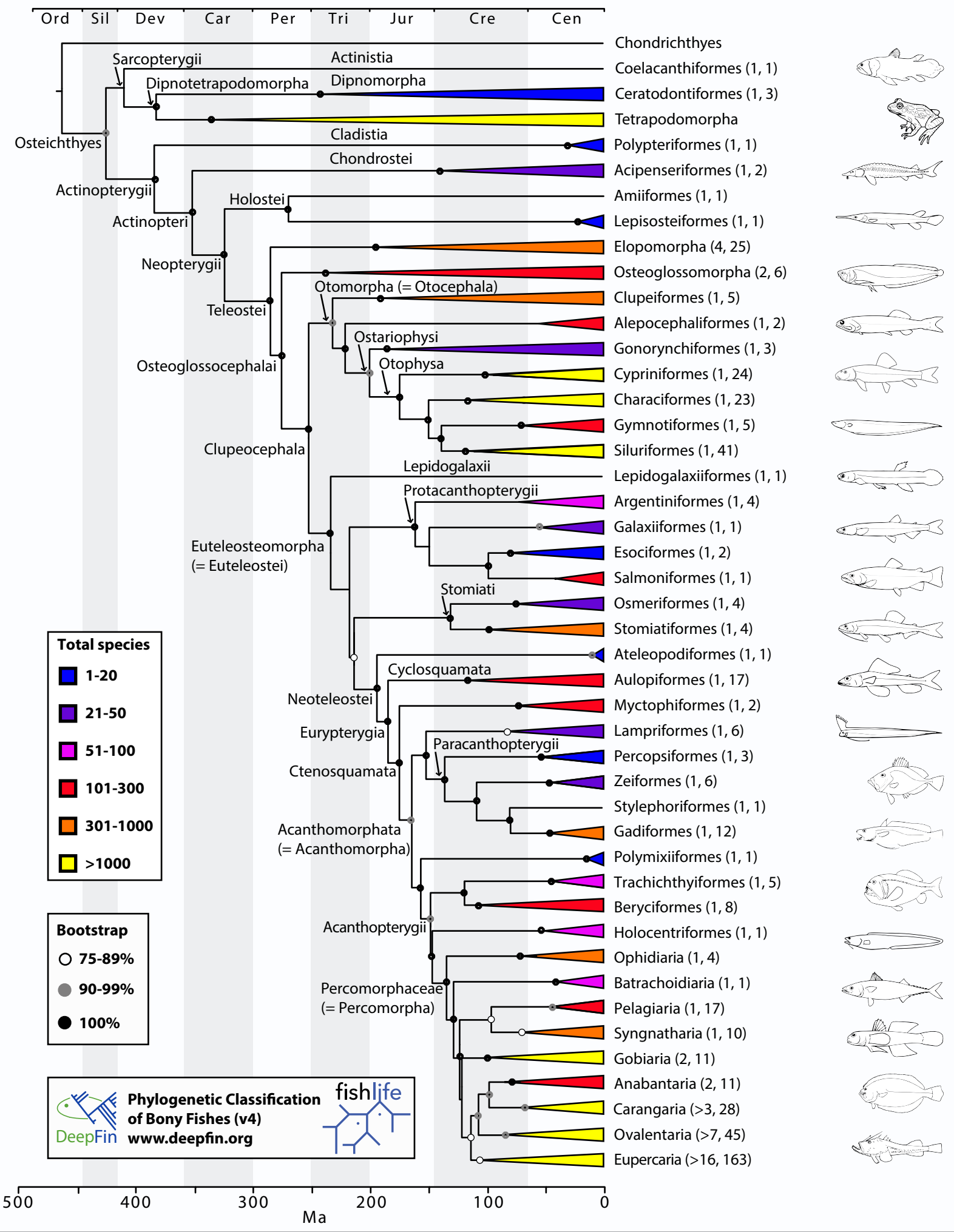

Supplement: Supplementary file 5 — High resolution image of Figure 1. (PDF 1120 kb) [file 12862_2017_958_MOESM5_ESM.pdf]
