# Supplementary material for: Phylogenetic classification of bony fishes
Source: BMC Evol Biol. 2017 Jul 6;17:162. doi: 10.1186/s12862-017-0958-3 (PMC5501477; doi:10.1186/s12862-017-0958-3)
Supplement: Supplementary file 6 — High resolution image of Figure 2. (PDF 4040 kb) [file 12862_2017_958_MOESM6_ESM.pdf]

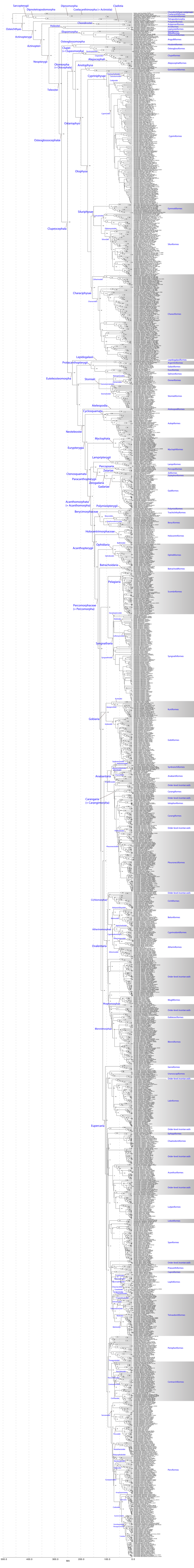

Sarcopterygii
Dipnotetrapodomorpha
Dipnomorpha
Coelacanthimorpha (= Actinistia)
Cladistia
Osteichthyes
Actinopterygii
Actinopteri
Chondrostei
Neopterygii
Holostei
Elopomorpha
Osteoglossomorpha
Clupei (= Clupeomorpha)
Otomorpha (= Otocephala)
Alepocephali
Anotophysa
Teleostei
Cypriniphysae
Osteoglossocephalai
Ostariophysi
Otophysa
Siluriphysae
Clupeocephala
Characiphysae
Lepidogalaxii
Protacanthopterygii
Euteleosteomorpha
Stomiati
Ateleopodia
Cyclosquamata
Neoteleostei
Myctophata
Eurypterygii
Lampripterygii
Percopsaria
Zeiariae
Ctenosquamata
Paracanthopterygii
Zeiogadaria
Gadariae
Acanthomorphata (= Acanthomorpha)
Polymixiipterygii
Berycimorphaceae
Holocentrimorphaceae
Ophidiaria
Acanthopterygii
Batrachoidaria
Pelagiaria
Percomorphaceae (= Percomorpha)
Syngnatharia
Gobiaria
Anabantaria
Carangaria (= Carangimorpha)
Cichlomorphae
Atherinomorphae
Ovalentaria
Mugilomorphae
Blenniimorphae
Eupercaria
Cypriniformes
Gymnotiformes
Siluriformes
Characiformes
Stomiatiformes
Aulopiformes
Myctophiformes
Gadiformes
Beryciformes
Holocentriformes
Ophidiiformes
Batrachoidiformes
Scombriformes
Syngnathiformes
Gobiiformes
Anabantiformes
Carangiformes
Pleuronectiformes
Cichliformes
Atheriniformes
Blenniiformes
Labriformes
Perciformes
500.0
400.0
300.0
200.0
100.0
0.0
MA
